# Supplementary material for: Inferring the mammal tree: Species-level sets of phylogenies for questions in ecology, evolution, and conservation
Source: PLoS Biol. 2019 Dec 4;17(12):e3000494. doi: 10.1371/journal.pbio.3000494 (PMC6892540; doi:10.1371/journal.pbio.3000494)
Supplement: S1 Table — Common authorities for mammals are MSW3 [85], IUCN [94], and the MDD [170]. IUCN, International Union for the Conservation of Nature; MDD, Mammal Diversity Database; MSW3, Mammal Species of the World, third edition. (DOCX) [file pbio.3000494.s016.docx]

S1 Table. The master taxonomy of this study vs. existing authoritative lists. Common authorities for mammals are Mammal Species of the World, vol. 3 [14] (MSW3), International Union for the Conservation of Nature [15] (IUCN), and the Mammal Diversity Database (MDD[11]).

| **Taxa** | **MSW3 2005** | **IUCN 2008** | **MDD v1 2018** | **Master taxonomy (this study)** |
| --- | --- | --- | --- | --- |
| Species |  |  |  |  |
| *Total* | 5,416 | 5,513 | 6,495 | 5,911 |
| *Extinct* | 1 | 77 | 96 | 107 |
| *Living* | 5,415 | 5,436 | 6,399 | 5,804 |
| *Living wild* | 5,415 | 5,436 | 6,382 | 5,791 |
| Genera | 1,230 | 1,226 | 1,314 | 1,283 |
| Families | 153 | 149 | 167 | 162 |
| Orders | 29 | 24 | 27 | 27 |
