# Supplementary material for: Inferring the mammal tree: Species-level sets of phylogenies for questions in ecology, evolution, and conservation
Source: PLoS Biol. 2019 Dec 4;17(12):e3000494. doi: 10.1371/journal.pbio.3000494 (PMC6892540; doi:10.1371/journal.pbio.3000494)
Supplement: S2 Table — Successive steps to parse results to unique NCBI species and subspecies names, match NCBI names to initially accepted names in the master taxonomy, and then manual addition (+) and removal (−) steps of error checks to yield per-gene final accepted species. excl., excluded; NCBI, National Center for Biotechnology Information. (DOCX) [file pbio.3000494.s017.docx]

S2 Table. Results from BLAST searches for each of the 31 gene fragments used in this study. Successive steps to parse results to unique NCBI species and subspecies names, match NCBI names to initially accepted names in the master taxonomy, and then manual addition (+) and removal (-) steps of error checks to yield per-gene final accepted species (excl. = excluded).

| **Gene** | **Region** | **(1) Unique BLAST hits** | | **(2) Unique NCBI taxa** | | **(3) Initial species** | | **(4a) Added manual (+)** | **(4b) Stop codons (-)** | **(4c) Rogue taxa (-)** | **(4d) Visual errors (-)** | **(5) Final species** | **Error-check excl.** |
| --- | --- | --- | --- | --- | --- | --- | --- | --- | --- | --- | --- | --- | --- |
| A2AB | exon | 792 | | 343 | | 323 | | 0 | 0 | 19 | 15 | 289 | 34 |
| ADORA3 | exon | 746 | | 500 | | 476 | | 1 | 0 | 46 | 3 | 428 | 48 |
| ADRB2 | exon | 662 | | 245 | | 235 | | 0 | 0 | 16 | 2 | 217 | 18 |
| APOB | exon | 1480 | | 681 | | 603 | | 4 | 1 | 17 | 3 | 586 | 17 |
| APP | NC | 1028 | | 472 | | 453 | | 0 | 0 | 39 | 0 | 414 | 39 |
| ATP7 | exon | 670 | | 506 | | 485 | | 0 | 0 | 17 | 0 | 468 | 17 |
| BCHE | exon | 462 | | 340 | | 324 | | 0 | 0 | 16 | 0 | 308 | 16 |
| BDNF | exon | 1241 | | 625 | | 590 | | 5 | 4 | 84 | 0 | 507 | 83 |
| BMI1 | NC | 400 | | 243 | | 232 | | 0 | 0 | 40 | 1 | 191 | 41 |
| BRCA1 | exon | 2350 | | 1,095 | | 992 | | 5 | 0 | 25 | 3 | 969 | 23 |
| BRCA2 | exon | 522 | | 326 | | 309 | | 0 | 0 | 3 | 0 | 306 | 3 |
| CNR1 | exon | 744 | | 399 | | 378 | | 0 | 0 | 23 | 0 | 355 | 23 |
| COI | mtDNA | 49,049 | | 2,326 | | 1,852 | | 0 | 0 | 158 | 9 | 1,685 | 167 |
| CREM | NC | 1889 | | 403 | | 385 | | 0 | 0 | 51 | 3 | 331 | 54 |
| CYTB | mtDNA | 89,218 | | 5,375 | | 3,787 | | 41 | 3 | 237 | 8 | 3,580 | 207 |
| DMP1 | exon | 684 | | 455 | | 434 | | 0 | 0 | 14 | 5 | 415 | 19 |
| EDG1 | exon | 655 | | 359 | | 341 | | 0 | 1 | 26 | 2 | 312 | 29 |
| ENAM | exon | 372 | | 262 | | 249 | | 0 | 0 | 2 | 0 | 247 | 2 |
| FBN1 | NC | 422 | | 345 | | 331 | | 0 | 0 | 26 | 4 | 301 | 30 |
| GHR | exon | 2113 | | 1,141 | | 1,071 | | 19 | 0 | 113 | 0 | 977 | 94 |
| IRBP | exon | 3361 | | 1,534 | | 1,412 | | 0 | 0 | 67 | 1 | 1,344 | 68 |
| ND1 | mtDNA | 32,483 | | 1,212 | | 1,007 | | 0 | 0 | 45 | 1 | 961 | 46 |
| ND2 | mtDNA | 32,902 | | 1,175 | | 1,014 | | 6 | 0 | 37 | 1 | 982 | 32 |
| PLCB4 | NC | 922 | | 554 | | 526 | | 0 | 0 | 48 | 0 | 478 | 48 |
| PNOC | exon | 701 | | 482 | | 460 | | 0 | 0 | 47 | 3 | 410 | 50 |
| RAG1a | exon | 1,234 | | 727 | | 682 | | 0 | 0 | 44 | 0 | 638 | 44 |
| RAG1b | exon | 1,871 | | 1,066 | | 983 | | 31 | 0 | 75 | 4 | 935 | 48 |
| RAG2 | exon | 2139 | | 1,108 | | 1,018 | | 4 | 0 | 131 | 3 | 888 | 130 |
| TTN | exon | 764 | | 375 | | 363 | | 0 | 0 | 19 | 0 | 344 | 19 |
| TYR1 | exon | 690 | | 380 | | 363 | | 0 | 0 | 28 | 0 | 335 | 28 |
| VWF | exon | 1276 | | 866 | | 826 | | 19 | 0 | 23 | 2 | 820 | 6 |
| **Total sequences** | | | 209,294 | 25,920 | 22,504 | | 135 | | 9 | 1,536 | 73 | 21,021 | 1,483 |
| **Total unique taxa** | | |  | 6,247 | 4,217 | | --- | | --- | --- | --- | 4,098 | 119 |
