# Supplementary material for: Inferring the mammal tree: Species-level sets of phylogenies for questions in ecology, evolution, and conservation
Source: PLoS Biol. 2019 Dec 4;17(12):e3000494. doi: 10.1371/journal.pbio.3000494 (PMC6892540; doi:10.1371/journal.pbio.3000494)
Supplement: S3 Table — Crown divergence mean Est and 95% CI (lower and upper) for each taxon listed, with the 27 extant mammal orders in capital letters. Ages in gray are order-level divergences estimated near the K-Pg extinction, with “near” defined as having 95% CI < 3 Ma of 66 Ma, whereas black ages have CIs >3 before 66 Ma. Our node-dated estimates are compared with global amino acid and DNA dates [60], best estimate dates [81], combined “14K+Mit” dates [61], rapid diversification posteriors [64], and fossil compendia [63,132]. Dates are missing if a node was not recovered or lacked taxon sampling. CI, confidence interval; Est, estimate; K-Pg, Cretaceous–Paleogene; Ma, million years ago. (DOCX) [file pbio.3000494.s018.docx]

**S3 Table.**

**Divergence times relative to prior studies.** Crown divergence mean estimates (Est) and 95% confidence intervals (CI, lower and upper) for each taxon listed, with the 27 extant mammal orders in capital letters. Ages in gray are order-level divergences estimated near the Cretaceous-Paleogene (K-Pg) extinction, with “near” defined as having 95% CI < 3 Ma of 66 Ma, while black ages have CIs >3 before 66 Ma. Our node-dated estimates are compared to global amino acid and DNA dates [20], best estimate dates [69], combined 14K+Mit dates [70], rapid diversification posteriors [71], and fossil compendia [65,72]. Dates are missing if a node was not recovered or lacked taxon sampling.

|  |  | **This study** | | | **Meredith et al. (2011)** | | | **Bininda-Emonds et al. (2007)** | | | **Dos Reis et al. (2012)** | | | **Ronquist et al. (2016)** | | | **Foley et al. (2016)** | | **PaleoBioDB** | |
| --- | --- | --- | --- | --- | --- | --- | --- | --- | --- | --- | --- | --- | --- | --- | --- | --- | --- | --- | --- | --- |
| **Taxon** | **N** | **Est** | **Low** | **Up** | **Est** | **Low** | **Up** | **Est** | **Low** | **Up** | **Est** | **Low** | **Up** | **Est** | **Low** | **Up** | **Fossil crown min** | **Fossil crown max** | **Fossil stem max** | **oldest genus/order** |
| Mammalia | 5911 | 188.4 | 166.7 | 210.9 | 217.8 | 203.3 | 238.2 | 166.2 | Fixed | Fixed | 184.7 | 174.6 | 191.9 | . | . | . | 164.9 | 227 | . | . |
| MONOTREMATA | 5 | 38.2 | 13.5 | 79.4 | 36.7 | 22.4 | 103.1 | 63.6 | 52.2 | 75 | 56.4 | 30.6 | 84.7 | . | . | . | 25 | 121 | 122.5 | *Kryoryctes* |
| Theria | 5906 | 159.6 | 156.3 | 166.2 | 190 | 167.2 | 215.3 | 147.4 | 141.8 | 153.1 | 172.8 | 168.5 | 177.9 | . | . | . | . | . | . | *.* |
| Marsupialia | 362 | 79.4 | 67.9 | 92.8 | 81.8 | 67.9 | 97.2 | 82.5 | 71.4 | 93.7 | 76.0 | 64.3 | 83.6 | . | . | . | 65.18 | 83.8 | . | . |
| PAUCITUBERCULATA | 7 | 16.4 | 12.3 | 20.3 | 11.7 | 7.2 | 16.2 | 33.2 | 26.3 | 40.1 | 27.8 | 11.0 | 47.0 | . | . | . | . | . | 59.0 | *Bardalestes* |
| DIDELPHIMORPHIA | 106 | 38.3 | 31.3 | 45.2 | 31.4 | 23 | 38.4 | 56.2 | 45.9 | 67.7 | 50.5 | 39.4 | 60.4 | . | . | . | 11.61 | 66 | 105.3 | *Pariadens* |
| Australidelphia | 100 | 63.4 | 54.1 | 74.8 | 64.2 | 53.7 | 75.5 | 66.8 | 63.8 | 74.2 | 70.2 | 58.7 | 76.6 | . | . | . | . | . | . | . |
| MICROBIOTHERIA | 1 | . | . | . | . | . | . | . | . | . | . | . | . | . | . | . | . | . | 66.0 | *Khasia* |
| NOTORYCTEMORPHIA | 2 | 6.1 | 3.6 | 9.0 | . | . | . | 9.3 | 8.3 | 10.2 | . | . | . | . | . | . | . | . | 23.0 | *Naraboryctes* |
| DASYUROMORPHIA | 78 | 34.1 | 27.7 | 40.9 | 30 | 22.1 | 41.7 | 31.3 | 16.2 | 55.8 | 46.0 | 35.6 | 54.9 | . | . | . | 15.97 | 54.65 | 58.7 | *Gaylordia* |
| PERAMELEMORPHIA | 22 | 29.5 | 24.1 | 35.2 | 28 | 21.1 | 37.1 | 36.2 | 30.6 | 41.8 | 40.9 | 30.8 | 51.4 | . | . | . | 4.36 | 54.65 | 28.4 | *Galadi* |
| DIPROTODONTIA | 146 | 48.7 | 41.1 | 57.5 | 52.8 | 42.4 | 64 | 54.1 | 52.4 | 55.8 | 57.5 | 46.5 | 64.9 | . | . | . | 24.7 | 54.65 | 33.9 | *Cercartetus* |
| Placentalia | 5544 | 91.8 | 77.4 | 105.0 | 101.3 | 92.1 | 116.8 | 98.5 | 93.2 | 108.1 | 89.2 | 87.9 | 90.4 | 85 | 76 | 93 | 65.2 | 131.5 | . | . |
| Xenarthra | 33 | 67.4 | 53.0 | 83.9 | 65.4 | 58.4 | 71.5 | 72.5 | 67.4 | 77.6 | 69.4 | 66.5 | 71.8 | 43 | 35 | 55 | 47.8 | 66 | . | . |
| CINGULATA | 21 | 37.6 | 27.7 | 47.9 | . | . | . | . | . | . | . | . | . | . | . | . | . | . | 66.0 | *Proeuphractus* |
| PILOSA | 12 | 59.8 | 46.8 | 75.3 | 56.4 | 49.2 | 62.9 | . | . | . | 62.7 | 57.5 | 66.9 | . | . | . | 31.5 | 66 | 58.7 | *Asiabradypus* |
| Afrotheria | 92 | 80.0 | 64.0 | 94.5 | 80.9 | 74.4 | 96.5 | 93.4 | 90.4 | 96.4 | 70.4 | 68.7 | 72.1 | 66 | 61 | 74 | . | . | . | . |
| Afroinsectiphilia | 75 | 77.5 | 62.4 | 92.3 | 78.6 | 71.8 | 95.2 | 93.2 | 90.2 | 96.2 | 64.7 | 61.2 | 67.9 | 56 | 45 | 66 | . | . | . | . |
| AFROSORICIDA | 55 | 70.4 | 56.5 | 85.8 | 68.2 | 56.8 | 88 | 85.2 | 81 | 89.3 | . | . | . | . | . | . | . | . | 63.3 | *Eudaemonema* |
| MACROSCELIDEA | 19 | 59.0 | 44.8 | 71.7 | 49.1 | 37.7 | 57.2 | 50.7 | 43.1 | 58.3 | 32.4 | 19.4 | 43.9 | . | . | . | 15.97 | 56 | 66.0 | *Cingulodon* |
| TUBULIDENTATA | 1 | . | . | . | . | . | . | . | . | . | . | . | . | . | . | . | . | . | 28.1 | *Orycteropus* |
| Paenungulata | 17 | 54.0 | 41.5 | 67.4 | 64.3 | 56 | 70.6 | 75.9 | 72.0 | 79.8 | 60.3 | 58.3 | 62.2 | 61 | 47 | 68 | 59.2 | 72.3 | . | . |
| HYRACOIDEA | 5 | 10.0 | 4.3 | 15.4 | 6.1 | 3.9 | 8.3 | 19.1 | 18.4 | 20 | 16.0 | 76 | 27.0 | . | . | . | 6.08 | 11.62 | 56.0 | *Megalohyrax* |
| PROBOSCIDEA | 7 | 10.1 | 4.8 | 16.2 | 5.3 | 1.8 | 8 | 19.5 | 7.6 | 31.4 | 8.4 | 3.4 | 15.9 | . | . | . | 6.8 | 11.62 | 59.2 | *Eritherium* |
| SIRENIA | 5 | 14.3 | 7.0 | 22.6 | 31.4 | 25 | 34.4 | 52.2 | 37.9 | 66.5 | 27.2 | 12.6 | 38.7 | . | . | . | 41.3 | 59.2 | 56.0 | *Prorastomus* |
| Boreoeutheria | 5419 | 83.5 | 73.1 | 94.8 | 92 | 82.9 | 107.6 | 96.1 | 92.9 | 98.4 | 83.8 | 82.8 | 84.9 | 81 | 74 | 90 | . | . | . | . |
| Laurasiatheria | 2456 | 75.1 | 66.3 | 84.1 | 84.6 | 78.5 | 93 | 87.8 | 85 | 90.5 | 76.0 | 74.9 | 77.0 | 75 | 68 | 82 | . | . | . | . |
| EULIPOTYPHLA | 491 | 74.3 | 65.4 | 83.6 | 77.3 | 70.7 | 85.8 | 82.5 | 79.8 | 85.3 | 61.5 | 60.9 | 62.0 | 56 | 44 | 69 | 61.6 | 83.8 | 61.7 | *Litolestes* |
| Erinaceidae | 24 | 46.7 | 39.1 | 53.7 | 38.9 | 29.0 | 48.9 | 65.5 | 60.9 | 70.1 | 48.2 | 43.1 | 52.7 | . | . | . | . | . | . | *.* |
| Solenodontidae | 3 | 10.9 | 0.2 | 39.6 | --- | --- | --- | 40.8 | 40.8 | 40.8 | --- | --- | --- | . | . | . | . | . | . | *.* |
| Soricidae | 420 | 47.2 | 39.5 | 55.2 | --- | --- | --- | 49.0 | 40.3 | 58 | 33.2 | 26.5 | 39.6 | . | . | . | . | . | . | *.* |
| Talpidae | 44 | 40.6 | 32.8 | 47.7 | --- | --- | --- | 61.8 | 52.3 | 71.3 | 34.5 | 29.5 | 39.4 | . | . | . | . | . | . | *.* |
| PHOLIDOTA | 8 | 27.1 | 17.9 | 38.2 | 25.3 | 16.9 | 35.7 | 19.1 | 7.3 | 46.9 | . | . | . | . | . | . | . | . | 48.6 | *Cryptomanis* |
| CARNIVORA | 298 | 40.2 | 33.9 | 47.9 | 54.7 | 47.4 | 60.6 | 63.4 | 59.8 | 67.1 | 54.2 | 52.3 | 56.0 | 44 | 35 | 61 | 38 | 66 | 66.0 | *Pappictidops* |
| Caniformes | 172 | 34.5 | 28.6 | 40.4 | 46.5 | 39.6 | 52.1 | 59.2 | 55.8 | 62.6 | 41.8 | 34.6 | 47.8 | . | . | . | 38 | 56 | . | . |
| Feliformes | 126 | 27.2 | 22.3 | 32.2 | 39.7 | 33.4 | 45.9 | 42.5 | 37.2 | 47.9 | 48.5 | 45.5 | 51.5 | . | . | . | . | . | . | . |
| PERISSODACTYLA | 24 | 38.7 | 32.6 | 45.0 | 56.8 | 55.1 | 61 | 55.8 | 51.1 | 61 | 52.6 | 41.8 | 61.0 | . | . | . | 55.5 | 61.6 | 59.2 | *Paschatherium* |
| ARTIODACTYLA | 348 | 58.4 | 50.3 | 67.1 | 65.4 | 62.3 | 68.5 | 70.7 | 67.6 | 73.7 | 61.4 | 60.7 | 62.3 | 54 | 47 | 60 | 52.5 | 66 | 66.0 | *Basilosaurus* |
| Ruminantia | 225 | 34.0 | 29.1 | 41.3 | 40.3 | 35.1 | 46.4 | 30.2 | 26.5 | 34.5 | 38.7 | 34.3 | 42.9 | . | . | . | . | . | . | . |
| Whippomorpha | 95 | 39.3 | 32.7 | 46.4 | 53.7 | 51.1 | 58.2 | 52.2 | 41.9 | 62.6 | 48.1 | 45.9 | 50.1 | . | . | . | 52.5 | 61.6 | . | . |
| Cetacea | 91 | 25.9 | 15.2 | 38.6 | 29.4 | 13.4 | 35.1 | 30.2 | 26.5 | 34.5 | 34.4 | 33.7 | 36 | . | . | . | 34 | 47.8 | . | . |
| CHIROPTERA | 1287 | 57.1 | 48.7 | 66.0 | 66.5 | 62.3 | 71.3 | 71.2 | 68 | 74.3 | 59.2 | 57.7 | 60.7 | 55 | 43 | 65 | 47.8 | 66 | 56.0 | *Ageina* |
| Yangochiroptera | 902 | 51.9 | 45.3 | 58.8 | 56.3 | 52.2 | 61.3 | . | . | . | 50.7 | 46.1 | 54.6 | . | . | . | 47.8 | 61.6 | . | . |
| Yinpterochiroptera | 385 | 50.5 | 41.4 | 60.3 | 62.9 | 58.6 | 66.8 | . | . | . | 53.4 | 49.2 | 56.5 | . | . | . | . | . | . | . |
| Euarchontoglires | 2963 | 77.0 | 67.8 | 87.4 | 83.3 | 74.1 | 97.8 | 91.8 | 90 | 93.8 | 75.7 | 74.8 | 76.7 | 76 | 69 | 85 | . | . | . | . |
| Primatomorpha | 480 | 70.4 | 61.7 | 79.8 | 82 | 73.7 | 97.4 | 88.5 | 85.9 | 91 | 74.1 | 73.2 | 75.1 | 65 | 58 | 73 | 65.2 | 83.8 | . | . |
| SCANDENTIA | 20 | 52.8 | 38.1 | 68.4 | 55.9 | 45 | 63.9 | 31.7 | 29.9 | 34.7 | . | . | . | . | . | . | 34 | 66 | 48.6 | *Eodendrogale* |
| DERMOPTERA | 2 | 9.6 | 3.3 | 16.5 | 7.4 | 4.5 | 13.2 | 15 | 10.2 | 19.9 | . | . | . | . | . | . | . | . | 63.3 | *Elpidophorus* |
| PRIMATES | 458 | 67.1 | 57.6 | 76.9 | 71.5 | 64.3 | 78.4 | 84.5 | 81.9 | 87.1 | 68.2 | 67.3 | 69.2 | 58 | 52 | 65 | 38 | 66 | 66.0 | *Pandemonium* |
| Catarrhini | 152 | 20.7 | 17.0 | 24.6 | 20.6 | 17.0 | 22.7 | 36.6 | 33.8 | 39.5 | 26.3 | 25.3 | 27.4 | . | . | . | 20.55 | 38 | . | . |
| Platyrrhini | 161 | 17.0 | 14.2 | 20.1 | 14.6 | 11.2 | 18.4 | 24.5 | 21.4 | 28.3 | 16.2 | 10.3 | 22.1 | . | . | . | 11.8 | 37.3 | . | . |
| Strepsirrhini | 135 | 49.6 | 42.2 | 57.0 | 55.1 | 50.2 | 58.7 | 75.5 | 71.3 | 79.6 | 55.1 | 53.4 | 56.8 | . | . | . | 38 | 56 | . | . |
| Glires | 2483 | 72.5 | 64.4 | 81.1 | 79.5 | 71.5 | 94.1 | 88.9 | 87.8 | 90.1 | 70.8 | 69.9 | 71.8 | 73 | 66 | 81 | . | . | . | . |
| LAGOMORPHA | 91 | 50.9 | 47.6 | 56.0 | 50.2 | 47.4 | 56.9 | 64.3 | 60 | 68.7 | 47.9 | 45.9 | 49.3 | . | . | . | 53.7 | 61.6 | 56.0 | *Aktashmys* |
| RODENTIA | 2392 | 67.9 | 60.5 | 75.2 | 69 | 64.1 | 74.8 | 82.8 | 80.2 | 85.4 | 64.4 | 63.5 | 65.3 | 67 | 61 | 73 | 56 | 66 | 58.7 | *Asiaparamys* |
| Guinea_pig-related | 304 | 61.7 | 53.0 | 69.3 | 61.1 | 56.1 | 68.3 | 56.5 | 52.0 | 68.5 | 32.3 | 19.5 | 43.2 | . | . | . | 40.94 | 56 | . | . |
| Mouse-related | 1768 | 65.0 | 58.4 | 71.6 | 65.1 | 60.7 | 70.0 | 81.0 | 78.4 | 84.5 | 60.0 | 55.1 | 56.7 | . | . | . | 54 | 66 | . | . |
| Squirrel-related | 320 | 49.8 | 42.1 | 57.4 | 60.5 | 55.5 | 64.2 | 80.1 | 78.2 | 81.1 | 52.8 | 42.7 | 58.4 | . | . | . | 47.8 | 61.6 | . | . |
