# Supplementary material for: Inferring the mammal tree: Species-level sets of phylogenies for questions in ecology, evolution, and conservation
Source: PLoS Biol. 2019 Dec 4;17(12):e3000494. doi: 10.1371/journal.pbio.3000494 (PMC6892540; doi:10.1371/journal.pbio.3000494)
Supplement: S4 Table — Fossil occs. per extant mammalian order were gathered from the Paleobiology Database. HPD, highest posterior density; occ., occurrences. (DOCX) [file pbio.3000494.s019.docx]

**S4 Table. Fossil maximum stratigraphic ages per order relative to stem ages from our node-dated phylogeny (95% HPD age of 10,000 trees).** Fossil occurrences (occs.) per extant mammalian order were gathered from the Paleobiology Database.

|  |  |  |  | **Phylogeny stem ages** | | |  |
| --- | --- | --- | --- | --- | --- | --- | --- |
| **Order** | **Fossil occs. per order** | **Oldest fossil genus** | **Fossil max age** | **Mean** | **Min** | **Max** | **Diff (tree max - fossil max)** |
| MONOTREMATA | 31 | *Kryoryctes* | 122.5 | 188.4 | 166.7 | 210.9 | 88.5 |
| **Marsupialia** |  |  |  |  |  |  |  |
| PAUCITUBERCULATA | 70 | *Bardalestes* | 59.0 | 79.4 | 67.9 | 92.8 | 33.8 |
| DIDELPHIMORPHIA | 281 | *Pariadens* | 105.3 | 74.4 | 63.2 | 87.1 | **-18.2** |
| MICROBIOTHERIA | 36 | *Khasia* | 66.0 | 63.4 | 54.1 | 74.8 | 8.8 |
| DIPROTODONTIA | 1011 | *Paljara* | 33.9 | 61.1 | 51.7 | 71.9 | 38.0 |
| NOTORYCTEMORPHIA | 2 | *Naraboryctes* | 23.0 | 60.0 | 50.6 | 70.6 | 47.5 |
| DASYUROMORPHIA | 202 | *Gaylordia* | 58.7 | 58.6 | 49.4 | 68.9 | 10.2 |
| PERAMELEMORPHIA | 79 | *Galadi* | 28.4 | 58.6 | 49.4 | 68.9 | 40.5 |
| **Placentalia** |  |  |  |  |  |  |  |
| Xenarthra |  |  |  |  |  |  |  |
| PILOSA (=Xenarthra) | 1184 | *Asiabradypus* | 58.7 | 67.4 | 53.0 | 83.8 | 25.1 |
| CINGULATA | 608 | *Proeuphractus* | 66.0 | 67.4 | 53.0 | 83.8 | 17.8 |
| Afrotheria |  |  |  |  |  |  |  |
| AFROSORICIDA | 192 | *Eudaemonema* | 63.3 | 74.8 | 61.0 | 90.8 | 27.5 |
| MACROSCELIDEA | 695 | *Cingulodon* | 66.0 | 74.8 | 61.0 | 90.8 | 24.8 |
| TUBULIDENTATA | 93 | *Orycteropus* | 28.1 | 77.5 | 62.4 | 92.3 | 64.2 |
| SIRENIA | 438 | *Prorastomus* | 56.0 | 54.0 | 41.5 | 67.3 | 11.3 |
| PROBOSCIDEA | 2665 | *Eritherium* | 59.2 | 50.2 | 38.5 | 63.8 | 4.6 |
| HYRACOIDEA | 176 | *Megalohyrax* | 56.0 | 50.2 | 38.5 | 63.8 | 7.8 |
| Laurasiatheria |  |  |  |  |  |  |  |
| EULIPOTYPHLA | 1442 | *Litolestes* | 61.7 | 75.1 | 66.3 | 84.1 | 22.4 |
| CHIROPTERA | 770 | *Ageina* | 56.0 | 70.0 | 61.7 | 79.3 | 23.3 |
| ARTIODACTYLA | 16374 | *Basilosaurus* | 66.0 | 61.9 | 53.1 | 69.6 | 3.6 |
| PERISSODACTYLA | 8948 | *Paschatherium* | 59.2 | 61.9 | 53.1 | 69.6 | 10.4 |
| CARNIVORA | 8877 | *Pappictidops* | 66.0 | 60.6 | 52.6 | 69.6 | 3.6 |
| PHOLIDOTA | 16 | *Cryptomanis* | 48.6 | 60.6 | 52.6 | 69.6 | 21.0 |
| Euarchontoglires |  |  |  |  |  |  |  |
| PRIMATES | 3181 | *Pandemonium* | 66.0 | 70.4 | 61.7 | 79.8 | 13.8 |
| DERMOPTERA | 29 | *Elpidophorus* | 63.3 | 60.9 | 50.1 | 72.4 | 9.1 |
| SCANDENTIA | 7 | *Eodendrogale* | 48.6 | 60.9 | 50.1 | 72.4 | 23.8 |
| LAGOMORPHA | 1971 | *Aktashmys* | 56.0 | 72.4 | 64.4 | 81.1 | 25.1 |
| RODENTIA | 15928 | *Asiaparamys* | 58.7 | 72.4 | 64.4 | 81.1 | 22.4 |
