# Supplementary material for: Inferring the mammal tree: Species-level sets of phylogenies for questions in ecology, evolution, and conservation
Source: PLoS Biol. 2019 Dec 4;17(12):e3000494. doi: 10.1371/journal.pbio.3000494 (PMC6892540; doi:10.1371/journal.pbio.3000494)
Supplement: S5 Table — Tip DR median, 95% confidence interval, and the skew in a given clade across 10,000 node-dated trees. Tests of the clade tip DR versus the (nonclade) background rate used the Mann–Whitney U statistic: greater (>, grayed), lesser (<), or NS. tip DR, tip-level pure-birth diversification rate; NS, not significant. (DOCX) [file pbio.3000494.s020.docx]

**S5 Table. Per clade summary of tip-level diversification rate (tip DR).** Tip DR median, 95% confidence interval, and the skew in a given clade across 10,000 node-dated trees. Tests of the clade tip DR versus the (non-clade) background rate used the Mann-Whitney U statistic: greater (>, grayed), lesser (<), or not significant (NS).

| **Taxon** | **Richness** | **Median** | **Low (2.5%)** | **High (97.5%)** | **Skew** | **Mann-Whitney U-test** | |
| --- | --- | --- | --- | --- | --- | --- | --- |
| Mammalia | 5804 | 0.211 | 0.056 | 0.480 | 0.145 | . |  |
| MONOTREMATA | 5 | 0.022 | 0.015 | 0.022 | -1.200 | *** | < |
| RODENTIA | 2354 | 0.208 | 0.061 | 0.482 | 1.193 | NS |  |
| Mouse-related | 1742 | 0.203 | 0.060 | 0.430 | 0.726 | * | < |
| Squirrel-related | 320 | 0.213 | 0.076 | 0.501 | 0.835 | NS |  |
| Guinea pig-related | 292 | 0.233 | 0.057 | 0.684 | 1.090 | ** | > |
| LAGOMORPHA | 90 | 0.265 | 0.113 | 0.543 | 0.595 | *** | > |
| CHIROPTERA | 1282 | 0.180 | 0.062 | 0.442 | 1.585 | *** | < |
| Yinpterochiroptera | 381 | 0.225 | 0.072 | 0.647 | 1.216 | ** | > |
| Yangochiroptera | 901 | 0.162 | 0.059 | 0.351 | 0.720 | *** | < |
| EULIPOTYPHLA | 484 | 0.229 | 0.053 | 0.486 | 0.753 | NS |  |
| Soricidae | 414 | 0.243 | 0.086 | 0.491 | 0.847 | *** | > |
| Talpidae | 44 | 0.128 | 0.043 | 0.221 | -0.003 | *** | < |
| Erinaceidae | 24 | 0.113 | 0.033 | 0.133 | -0.797 | *** | < |
| SCANDENTIA | 20 | 0.110 | 0.022 | 0.137 | -1.163 | *** | < |
| DERMOPTERA | 2 | . | . | . | . | . |  |
| PRIMATES | 450 | 0.326 | 0.093 | 0.480 | -0.431 | *** | > |
| Simiiformes | 309 | 0.345 | 0.183 | 0.500 | -0.127 | *** | > |
| Catarrhini | 149 | 0.346 | 0.174 | 0.511 | -0.013 | *** | > |
| Platyrrhini | 160 | 0.344 | 0.221 | 0.487 | -0.211 | *** | > |
| Strepsirrhini | 131 | 0.259 | 0.084 | 0.397 | -0.194 | ** | > |
| PERISSODACTYLA | 18 | 0.106 | 0.062 | 0.171 | 0.074 | *** | < |
| ARTIODACTYLA | 338 | 0.248 | 0.079 | 0.537 | 0.432 | *** | > |
| Ruminantia | 217 | 0.260 | 0.090 | 0.502 | 0.163 | *** | > |
| Whippomorpha | 93 | 0.247 | 0.075 | 0.602 | 0.599 | *** | > |
| PHOLIDOTA | 8 | 0.057 | 0.054 | 0.072 | 0.983 | *** | < |
| CARNIVORA | 286 | 0.238 | 0.084 | 0.458 | 0.305 | *** | > |
| Feliformes | 122 | 0.259 | 0.089 | 0.473 | 0.153 | *** | > |
| Caniformes | 164 | 0.232 | 0.084 | 0.426 | 0.398 | * | > |
| CINGULATA | 21 | 0.068 | 0.047 | 0.092 | -0.034 | *** | < |
| PILOSA | 10 | 0.047 | 0.024 | 0.058 | -0.742 | *** | < |
| AFROSORICIDA | 55 | 0.082 | 0.032 | 0.149 | 0.189 | *** | < |
| MACROSCELIDEA | 19 | 0.061 | 0.044 | 0.068 | -0.630 | *** | < |
| TUBULIDENTATA | 1 | . | . | . | . | . |  |
| PROBOSCIDEA | 2 | . | . | . | . | . |  |
| HYRACOIDEA | 5 | 0.063 | 0.056 | 0.063 | -0.408 | *** | < |
| SIRENIA | 4 | . | . | . | . | . |  |
| Marsupialia | 350 | 0.207 | 0.055 | 0.414 | 0.752 | NS |  |
| DIPROTODONTIA | 139 | 0.262 | 0.055 | 0.530 | 0.325 | *** | > |
| DIDELPHIMORPHIA | 105 | 0.207 | 0.075 | 0.336 | 0.008 | NS |  |
| DASYUROMORPHIA | 77 | 0.183 | 0.104 | 0.270 | 0.058 | ** | < |
| PERAMELEMORPHIA | 19 | 0.189 | 0.102 | 0.215 | -1.031 | NS |  |
| NOTORYCTEMORPHIA | 2 | . | . | . | . | . |  |
| PAUCITUBERCULATA | 7 | 0.084 | 0.070 | 0.099 | -0.155 | *** | < |
| MICROBIOTHERIA | 1 | . | . | . | . | . |  |

*** P < 0.001; ** P < 0.01; * P < 0.05
