# Supplementary material for: Inferring the mammal tree: Species-level sets of phylogenies for questions in ecology, evolution, and conservation
Source: PLoS Biol. 2019 Dec 4;17(12):e3000494. doi: 10.1371/journal.pbio.3000494 (PMC6892540; doi:10.1371/journal.pbio.3000494)

Supplementary Information for

Inferring the mammal tree: species-level sets of phylogenies for questions in ecology, evolution, and conservation

Nathan S. Upham, Jacob A. Esselstyn, and Walter Jetz

Nathan S. Upham
Email: nathan.upham@yale.edu

Walter Jetz
Email: walter.jetz@yale.edu

This PDF file includes:

- Table of Contents
- Supplementary Methods
- Supplementary Results
- S1 to S10 Figures
- S1 to S5 Tables
- References for SI reference citations

Other supplementary materials for this manuscript include the following:

- S1 to S5 Data

Table of Contents (click to navigate)

Supplementary Methods **1**

1. Experimental design **1**

S1 Fig. **1**

2. Taxonomic matchup of mammalian species names **2**

S2 Fig. **2**

S1 Table. **3**

3.1 DNA alignments and pseudogenes 3

3.2 Gene tree construction and rogue taxon identification 4

3.3 Visual error-checking of gene trees 4

3.4 Final gene trees 5

S3 Fig. **5**

S2 Table. **6**

4. Global genetic scaffold tree using maximum likelihood **7**

4.1 Supermatrix and PartitionFinder 7

5. Patch clade trees and PASTIS completion of missing species **7**

6. Fossil-dated backbone trees **8**

6.1 Node-dating backbone analyses 8

6.2 Tip-dating backbone analyses 9

7. Construction of full dated mammalian phylogenies **9**

Supplementary Results and Discussion **10**

8. Supplemental comparisons to previous mammal studies **10**

8.1 Backbone divergence times 10

Supplementary Acknowledgments **11**

9. Mammal artwork used in the manuscript **11**

Supplementary References **16**

S3-S5 Tables **21**

S4-S10 Figures **27**

Supplementary Methods

1. Experimental design

The decision to divide Mammalia into smaller clades for analyses – rather than perform tree-wide dated inference – is based on trade-offs in the following factors:

(i) computational limits to adding tips in phylogenetic analyses;
(ii) propagating uncertainty in inference; and
(iii) propagating uncertainty in models of evolution.

We found the maximum tree size for Bayesian inference in MrBayes v.3.2.6 [1] was ~800 species in our trial runs. Near this maximum, our largest patch (Muridae, 778 species) took 3.7 weeks to finish 33,330,000 generations in parallel on 16 BEAGLE-enabled compute nodes. We suggest a current hard limit of < 1000 species for convergence of Bayesian supermatrix analyses (matrix complexity was also a key determinant of run times, see section 5.1).

Our goal of including full uncertainty of divergence times and species relationships was best suited to Bayesian inference, where the likelihood of parameter estimates is reflected by their posterior frequency [2–5]. In contrast, maximum-likelihood and parsimony approaches to find the single 'best' point estimate effectively collapse tree uncertainty and can result in false confidence [2]. The ideal of using the multi-species coalescent to co-estimate genealogies and the species tree [6] in a Bayesian framework is unfortunately limited to ~25 species (e.g., *BEAST [7,8]). ML-based models (e.g., ASTRAL-II and ASTRAL-III [9,10]) have extended applications of the coalescent to larger trees, but analyzing the breadth of Mammalia in this context again only produces consensus trees, and requires subsequent steps of time calibration. Overall, we emphasize that the 'big tree' territory beyond ~1000 species requires new types of phylogenetic assumptions to overcome computational limits to co-estimation (S1 Fig).

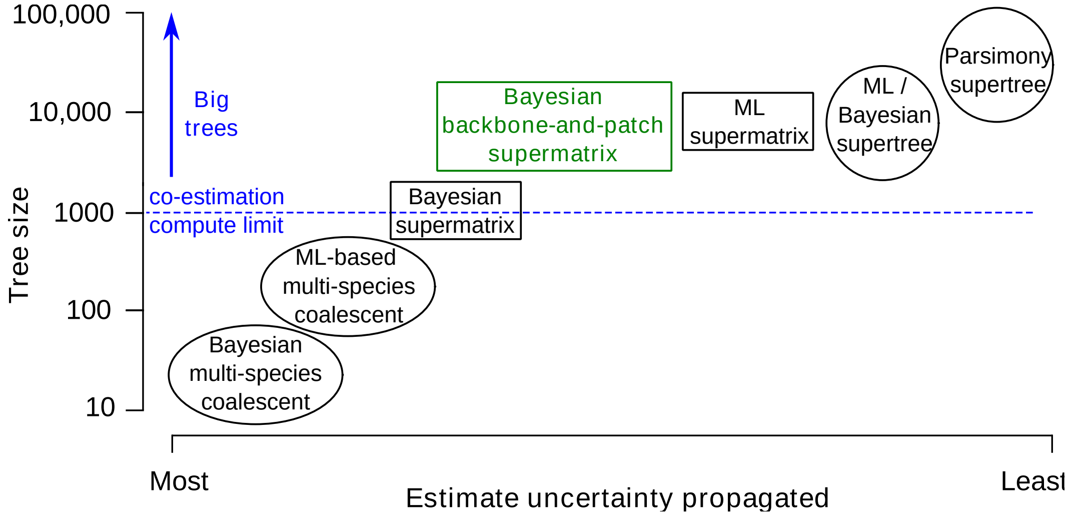

S1 Fig.
Trade-off between tree size and statistical uncertainty in the tree. Computational costs increase with tree size and the realism of the evolutionary models, resulting in reduced ability to propagate uncertainty in larger trees. We suggest a current upper limit for Bayesian co-estimation, beyond which performing supermatrix analyses requires our backbone-and-patch approach (green) to divide the tree into smaller sub-analyses.

2. Taxonomic matchup of mammalian species names

Our complex NCBI-to-IUCN taxonomic matchup (S2 Fig) produced a master taxonomy of 5911 mammalian species for this study. We retained 5501 species names from the 5513 species in the original IUCN list, synonymizing 12 species within existing IUCN names (“lumps”). We added 410 species to the IUCN base taxonomy, as follows: (i) 367 new species that were described since the ~2008 cutoff for most of the IUCN list (about 200 updates were made between 2009-2014); (ii) 13 domestic species; and (iii) 30 recently extinct species. The Mammal Diversity Database ([11,12]; https://mammaldiversity.org), which includes 6495 extant and recently extinct species as of 15 August 2017 (version 1.0), was an outgrowth of our project and continues to update mammalian taxonomy as new literature is published.

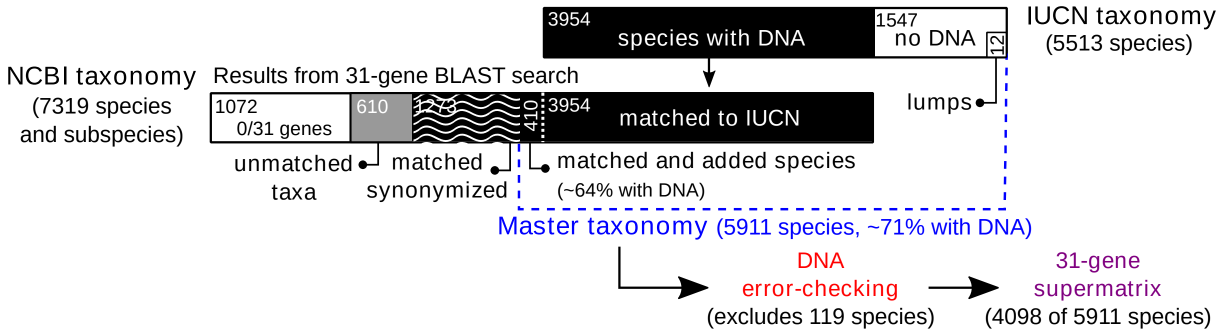

S2 Fig.
Our master taxonomy merges the mammal lists of NCBI (genetic data) and IUCN (name authority). Bars representing each taxonomic list are sized proportionately to the number of names in each list category. We conducted a baited BLAST search of NCBI using 31 genes, from which data was returned for ~85% of the NCBI names (6247 of 7319; list of 20 April 2015). Steps of synonym matching and taxon addition to the IUCN list resulted in our master taxonomy of 5911 species. Black corresponds to the 4217 species initially with DNA, including 410 species we added to the IUCN list (newly described species, domestic forms, or recently extinct), which was reduced to 4098 species after error-checking steps (see S1 and S2 Data).

For the new species, we integrated these names to the master taxonomy either as derived from portions of existing IUCN names (“splits”; 33 species), or else as new discoveries (“de novo”; 334 species). All splits had associated DNA sequences published with their taxonomic changes, while 56% of de novo species had DNA sampling for 1 or more of our targeted markers. Domestic species were in most cases sampled for all targeted loci (e.g., guinea pig, camel, sheep, goat) and therefore helped anchor their wild relatives in the phylogeny. Each of the recently extinct species that we added were sampled for at least 1 of our targeted loci and therefore were useful for dividing long branches in the tree (e.g., *Archeolemur*, *Mammut*, *Mylodon*). However, we did not attempt to add every Pleistocene extinct mammal (as did [13]). We also matched 17 of the 77 extinct species from the IUCN list (either extinct in the wild or overall) with 1 or more DNA sequences, yielding DNA for 47 of 107 extinct species in our list. We additionally followed recent taxonomic revisions to give new genus assignments to 76 species in the IUCN list (denoted as “name transfers” in S1 Data; see file for full accounts and references of taxonomic changes and the master taxonomy used in this study). Our master taxonomic list contains 5804 species considered extant out of 5911 species total, which is nearly 400 more than MSW and IUCN (S1 Table).

S1 Table.
The master taxonomy of this study vs. existing authoritative lists. Common authorities for mammals are Mammal Species of the World, vol. 3 [14] (MSW3), International Union for the Conservation of Nature [15] (IUCN), and the Mammal Diversity Database (MDD[11]).

**Taxa**	**MSW3 2005**	**IUCN 2008**	**MDD v1 2018**	**Master taxonomy (this study)**
Species				
Total	5,416	5,513	6,495	5,911
Extinct	1	77	96	107
Living	5,415	5,436	6,399	5,804
Living wild	5,415	5,436	6,382	5,791
Genera	1,230	1,226	1,314	1,283
Families	153	149	167	162
Orders	29	24	27	27

3. Error-checking via iterative DNA sequence alignment and gene tree construction

The abundance of annotation errors on GenBank (e.g., incorrect taxon or gene identifications; [16–18]) is well-known. To address this, we used a per-gene approach to iteratively clean DNA data via (i) alignment, (ii) trees, and (iii) error-checking.

3.1 DNA alignments and pseudogenes

For the 26 coding fragments (mtDNA and exons), we aligned each to the appropriate amino acid reading frame using MACSE v1.0 [19], aiming to minimize stop codons with reference to per-gene guide alignments (e.g., from Meredith et al. [20]). This procedure allowed us to identify 30 sequences with stop codons that we confirmed as nuclear mitochondrial copies (numts) or pseudogenes; of these, 21 were replaced with another sequence from GenBank while the remaining 9 had no appropriate replacement. Gene sequences of ENAM and IRBP containing known loss-of-function stop codons were retained for enamel-absent (e.g., baleen whales, xenarthrans) and blind (e.g., blind mole-rats) species of mammals, respectively. Additional sequence alignment was performed in MAFFT version 7.245 [21] with manual checking.

We aligned the 5 non-coding (NC) nuclear genes using PRANK v.140603 [22], an algorithm which is able to distinguish between insertions and deletion events in the alignment. Ambiguity in the resulting alignments was reduced using the “-gappyout” method in trimAl v1.2b [23] to exclude the most gap-rich columns of the alignment. We visually inspected all alignments for clear errors in homology, and in several cases excluded unaligned (entirely non-overlapping) sequences. The full list of excluded sequences is in S1 Data.

3.2 Gene tree construction and rogue taxon identification

We generated maximum-likelihood (ML) gene trees for several additional rounds of sequence verification. We used RAxML v.8.2.3 [24], the GTRCAT model of DNA evolution, and the "-f a" option to specify 1000 rapid bootstrap replicates for all gene trees. Outgroup selection was a key step of this process since sequences of the same outgroup taxon were not available across all 31 gene fragments. In fact, the analyses of Meredith et al. [20] suffered from the same problem: 10 of the 27 gene fragments in their concatenated analyses lacked a non-mammalian outgroup (3, 4, or 5 outgroup taxa were sampled for their remaining genes; S3 Table). When available (for 21 genes), we used the lizard *Anolis carolinensis* as the outgroup to mammals, since together they form the clade Synapsida [25]. Otherwise, for 4 genes we used a monotreme outgroup (*Ornithorhynchus anatinus*) to a therian ingroup, and for 6 genes a marsupial outgroup (*Monodelphis domestica*) to a placental ingroup (S3 Table). For those 10 genes, we pruned alignments to the ingroup and single outgroup for purposes of sequence validation, but in downstream analyses retained all mammals.

For each gene, we identified 'rogue taxa' [26,27] using RogueNaRok v1.0 [27] on the best-scoring ML tree and bootstrap sample of 1000 trees as input. Rogues are the taxa most variable in gene tree placement due to ambiguous phylogenetic signal in a particular sequence [28]. We expected the number of rogues in an alignment to be related to the site-by-taxon coverage pattern, and to corresponding metrics of fractional decisiveness used to measure a matrix's power to distinguish between terraces of phylogenetic trees with similar likelihood [29]. By pruning out rogues, we improved the overall information content of resulting gene trees as measured using the relative bipartition information criterion (RBIC) [27]. This procedure was therefore conservative. The number of rogues identified per gene varied from 2 (ENAM; 0.8% of input sequences) to 237 (CYTB; 6.2%), with a mean of 49.6 rogues per gene (6.8%). In total, we identified 1537 rogues that, when excluded from each alignment, improved mean per-gene RBIC by 0.014 (max: 0.025 in ADORA3).

To test whether excluding additional rogue sequences might further improve alignments, we built a second round of ML gene trees on the rogue-pruned alignments as input for RogueNaRok. We found that a mean RBIC improvement of 0.002 per gene could be obtained by excluding 354 more rogues (mean of 11 per gene). However, given this small change we decided instead to retain those DNA sequences to maximize taxon sampling.

3.3 Visual error-checking of gene trees

We identified an additional 73 erroneous sequences by visually examining each ML gene tree after rogue-taxon exclusion. We flagged sequences based on expectations of monophyly in established higher clades (e.g., orders), and then investigated each flagged sequence for errors in labeling or alignment. Of these sequences, 28 were NCBI author-based errors of sequence contamination or mislabeling (including four cases of *Rattus* contamination), as well as four additional pseudogene sequences. We also note that four sequences from Meredith et al. [20] appear to be mis-annotated: COI: *Sapajus apella* (Primates; JN380205) as identical to *Canis lupus* (Carnivora; KF661058); CREM: *Pecari tajacu* (Artiodactyla; JN633478) as highly similar to *Ailuropoda melanoleuca* (Carnivora); RAG2: *Cratogeomys castanops* (Geomyidae; JN633333) as more similar to *Octodontomys gliroides* (Octodontidae; JN633335) than other Geomyidae; and VWF: *Ctenomys boliviensis* (Ctenomyidae; JN415078) as more similar to *Proechimys* (Echimyidae) than other Ctenomyidae. All erroneous sequences were excluded from

downstream analyses (see S1 Data for the full details of pseudogenes, stop codons, rogue taxa, and contaminants detected, with accession numbers).

In total, our error-checking steps excluded 1618 sequences across all genes (i.e., 7.2% of the 22,504 individual DNA sequences; S2 Table). This corresponded to excluding 119 species from the 31-gene supermatrix due to unreliable data, yielding 4098 species with at least 1 gene fragment present and validated in the final supermatrix (S3 Fig for genetic sampling of genera and families).

3.4 Final gene trees

Final gene trees were calculated in RAxML with the GTRCAT model and 1000 rapid bootstrap replicates. These large phylogenies were plotted in the R programming language [30] using the function "splitplotTree" in the phytools package [31], and with nodal support values annotated using the phyloch package (final gene tree files and plotted versions in S2 Data).

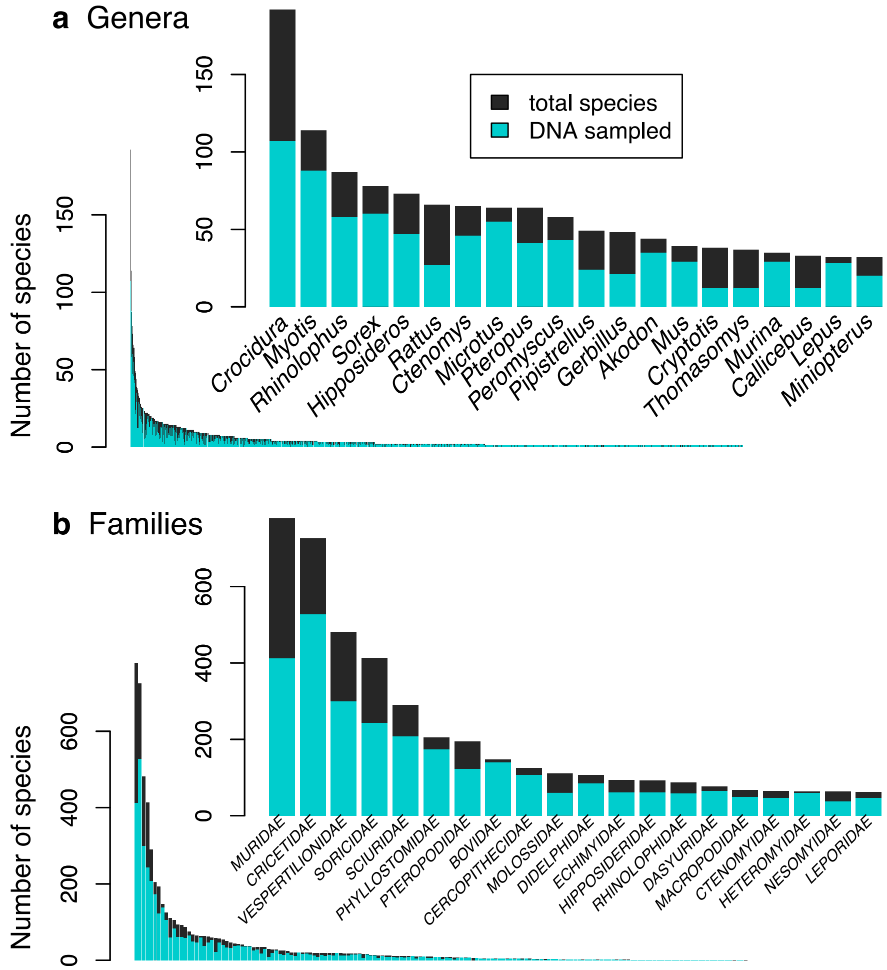

S3 Fig. Final genetic sampling of mammal species from NCBI relative to total diversity of genera and families. Species were considered sampled for DNA if one or more of our 31 genes were sampled in the final supermatrix after DNA cleaning, error-checking, and taxonomic reconciliation. Inset are detailed views of the genetic sampling within the 20 most speciose genera (of 1283 total) and families (of 127 total). Much additional DNA sequencing is needed to move from the 4,098 species with genetic sampling to the >6,000 species of modern mammals.

S2 Table.

Results from BLAST searches for each of the 31 gene fragments used in this study. Successive steps to parse results to unique NCBI species and subspecies names, match NCBI names to initially accepted names in the master taxonomy, and then manual addition (+) and removal (-) steps of error checks to yield per-gene final accepted species (excl. = excluded).

Gene	Region	(1) Unique BLAST hits	(2) Unique NCBI taxa	(3) Initial species	(4a) Added manual (+)	(4b) Stop codons (-)	(4c) Rogue taxa (-)	(4d) Visual errors (-)	(5) Final species	Error-check excl.
A2AB	exon	792	343	323	0	0	19	15	289	34
ADORA3	exon	746	500	476	1	0	46	3	428	48
ADRB2	exon	662	245	235	0	0	16	2	217	18
APOB	exon	1480	681	603	4	1	17	3	586	17
APP	NC	1028	472	453	0	0	39	0	414	39
ATP7	exon	670	506	485	0	0	17	0	468	17
BCHE	exon	462	340	324	0	0	16	0	308	16
BDNF	exon	1241	625	590	5	4	84	0	507	83
BMI1	NC	400	243	232	0	0	40	1	191	41
BRCA1	exon	2350	1,095	992	5	0	25	3	969	23
BRCA2	exon	522	326	309	0	0	3	0	306	3
CNR1	exon	744	399	378	0	0	23	0	355	23
COI	mtDNA	49,049	2,326	1,852	0	0	158	9	1,685	167
CREM	NC	1889	403	385	0	0	51	3	331	54
CYTB	mtDNA	89,218	5,375	3,787	41	3	237	8	3,580	207
DMP1	exon	684	455	434	0	0	14	5	415	19
EDG1	exon	655	359	341	0	1	26	2	312	29
ENAM	exon	372	262	249	0	0	2	0	247	2
FBN1	NC	422	345	331	0	0	26	4	301	30
GHR	exon	2113	1,141	1,071	19	0	113	0	977	94
IRBP	exon	3361	1,534	1,412	0	0	67	1	1,344	68
ND1	mtDNA	32,483	1,212	1,007	0	0	45	1	961	46
ND2	mtDNA	32,902	1,175	1,014	6	0	37	1	982	32
PLCB4	NC	922	554	526	0	0	48	0	478	48
PNOC	exon	701	482	460	0	0	47	3	410	50
RAG1a	exon	1,234	727	682	0	0	44	0	638	44
RAG1b	exon	1,871	1,066	983	31	0	75	4	935	48
RAG2	exon	2139	1,108	1,018	4	0	131	3	888	130
TTN	exon	764	375	363	0	0	19	0	344	19
TYR1	exon	690	380	363	0	0	28	0	335	28
VWF	exon	1276	866	826	19	0	23	2	820	6
**Total sequences**		209,294	25,920	22,504	135	9	1,536	73	21,021	1,483
**Total unique taxa**			6,247	4,217	---	---	---	---	4,098	119

4. Global genetic scaffold tree using maximum likelihood

4.1 Supermatrix and PartitionFinder

Concatenation of the 31 gene fragments was performed in Geneious v.9.1 [32], resulting in a sites-by-taxon supermatrix of 39,099 base pairs (bp) and 4098 species (11.9% complete for ungapped sites). We evaluated partitioning schemes for the supermatrix using PartitionFinder v.1.1.1 [33] and three *a priori* sets of partitions: (i) 83 partitions, one for each coding gene by codon position (26 × 3) plus the five non-coding genes; (ii) 31 partitions, one for each gene; and (iii) 11 partitions, one for each nuclear and mtDNA coding region by codon position (2 × 3) plus the 5 non-coding genes. We ran PartitionFinder using the full concatenated alignment, relaxed cluster searching (top 10% of schemes; [34]), linked branch lengths, and BIC model selection, finding that those partition sets could be reduced to 38, 19, and 9 partitions, respectively.

The best BIC score was for the simplest 9-partition model (6,790,459.3; the 38- and 19-partition models had BIC scores of 6,844,238.7 and 6,933,022.3). This model has a combined partition for APP, CREM, and FBN1, and then 1 partition each for BMI1; PLCB4; and first, second and third codon partitions for nDNA exons as well as for mtDNA fragments. We subsequently used the 9-partition model for all ensuing phylogenetic analyses.

5. Patch clade trees and PASTIS completion of missing species

For each patch clade, we performed four parallel runs of MrBayes on XSEDE, each run consisting of four chains of Markov chain Monte Carlo (MCMC; 3 heated and 1 cold), and sampled every 10,000 steps for 33,330,000 generations. Here is an example:

```
begin MRBAYES;
<define charsets of 9 partitions>

partition matrices = 9: nDNA_pos1, nDNA_pos2, nDNA_pos3, mtDNA_pos1,
mtDNA_pos2, mtDNA_pos3, APP_CREM_FBN1, BMI1, PLCB4;
set partition = matrices;

lset applyto = (all) nst=6 rates=gamma;          [GTR+G model]
unlink statefreq=(all) revmat=(all) shape=(all); [model unlink by partition]
prset applyto=(all) ratepr=variable;             [partition-specific rates]

<define topology constraints>
prset topologypr=constraints(<list constraint names>)

prset brlenspr = clock:birthdeath;               [clock trees, no fossils]
prset speciationpr = exp(10);                    [net diversification rate]
prset extinctionpr = beta(1,1);                  [relative extinction rate]
prset sampleprob = 1.0;                          [taxon sampling complete]
prset clockvarpr=igr;                            [independent gamma rates]
prset igrvarpr=exponential(10);                  [prior on IGR variance]
prset clockratepr = Fixed(1.0);                  [default: time unit is number
                                                  of expected
                                                  substitutions/site]
mcmcp ngen= 33330000 nruns=4 nchains=4
samplefreq=10000 printfreq=10000 savebrlens=yes; [mcmc settings]
propset ParsSPRClock(Tau{all},V{all})$warp=0.01; [parsimony-based topology
                                                  proposals to speed up
                                                  searches]
mcmc;                                            [start mcmc]
```

6. Fossil-dated backbone trees

6.1 Node-dating backbone analyses

Fossil calibrations for the constraint of mammalian node ages were selected from the compendium of Benton et al. [35], as augmented by Philips [36], and using the following criteria [37]: (i) confidence in placement, especially in cladistic analyses; (ii) confidence in the monophyly of the age-constrained node, which was a requirement of MrBayes; and (iii) lack of conflict in age priors, determined both in proximity of node constraints and empirically with trial runs in MrBayes. In total, we decided upon 17 optimal age constraints (also S5 Data):

1. **Mammalia**: 164.9 Ma minimum age to 201.5 Ma maximum age (set by Jurassic-Triassic boundary). Minimum defined by *Ambondro mahabo*, member of the monotreme clade Australosphenida from the middle Jurassic (Bathonian) of Madagascar [35,38–40].
2. **Monotremata**: 15.97 to 113.0 Ma (lower Albian, early Cretaceous). *Obdurodon dicksoni*, stem member of Ornithorhynchidae from the early Miocene (Burdigalian) of northwestern Queensland, Australia [36,41,42].
3. **Marsupialia**: 47.6 to 131.3 Ma (lower Barremian, early Cretaceous). *Djarthia murgonensis*, crown marsupial from the early Eocene (Ypresian) of Murgon, Australia [35,43].
4. **Macropodoidea**: 15.97 to 54.65 Ma (earliest Eocene). *Ganguroo bilamina*, stem or crown member of Macropodidae (to the exclusion of Potoroidae from the early Miocene (Burdigalian) of northwestern Queensland, Australia [36,44].
5. **Theria**: 156.3 to 169.6 Ma (lower Bathonian, middle Jurassic). *Juramaia sinensis*, stem eutherian from the late Jurassic (Oxfordian) of the Northeastern China [35,40].
6. **Placentalia**: 61.6 to 164.6 Ma (lower Oxfordian, late Jurassic). *Ravenictis krausei*, stem carnivoran from the early Paleocene (Danian) of Saskatchewan, Canada [35,45].
7. **Afrotheria**: 56.0 to 164.6 Ma (lower Oxfordian, late Jurassic). *Eritherium azzouzorum*, stem proboscidean from the upper Paleocene (early Thanetian) of Morocco [35,46].
8. **Xenarthra**: 47.6 to 164.6 Ma (lower Oxfordian, late Jurassic). *Riostegotherium yanei*, stem cingulate from the early Eocene (Ypresian) of Itaboraí, Brazil [35,47].
9. **Chiroptera**: 45.0 to 58.9 Ma (lower Thanetian, Paleocene). *Tanzanycteris mannardi*, stem member of Rhinolophoidea within Yinpterochiroptera from the middle Eocene (Lutetian) of Tanzania [36,48].
10. **Lagomorpha**: 47.6 to 66.0 Ma (earliest Paleocene). Unnamed stem leporid fossils from the early Eocene (Ypresian) of West-Central India [35,49].
11. **Rodentia**: 56.0 to 66.0 Ma (earliest Paleocene). *Paramys atavus*, stem member of Sciuromorpha from the late Paleocene (Thanetian) of Montana, USA that is nested well within crown Rodentia [35,50].
12. **Caviomorpha-Phiomorpha**: 40.94 to 56.0 Ma (upper Thanetian, latest Paleocene). *Cachiyacuy contamanensis*, stem caviomorph rodent from the middle Eocene (Lutetian) of Yahuarango, Peru [36,51].
13. **Primates**: 56.0 to 66.0 Ma (earliest Paleocene). *Altiatlasius koulchii*, stem anthropoid from the late Paleocene (Thanetian) of Morocco [35,52].
14. **Strepsirhini**: 33.9 to 56.0 Ma (upper Thanetian, latest Paleocene). *Karanisia clarki*, stem lorisiform or a crown strepsirrhine of uncertain affinities from the late Eocene (Priabonian) of Fayûm, Egypt [35,53,54].
15. **Anthropoidea**: 33.9 to 66.0 Ma (earliest Paleocene). *Catopithecus browni*, crown Catarrhini from late Eocene (Priabonian) Fayûm, Egypt [35,54].

16. **Artiodactyla**: 52.4 to 66.0 Ma (earliest Paleocene). *Himalayacetus subathuensis*, stem member of Cetacea from the early Eocene (Ypresian) of India [35,55,56].
17. **Carnivora**: 37.3 to 66.0 Ma (earliest Paleocene). *Hesperocyon gregarius*, stem caniform from the middle Eocene (Bartonian) of Saskatchewan, Canada [35,57].

Monophyly constraints were enforced on each calibrated node plus the root, which we calibrated with a uniform distribution of 318.0-332.9 Ma based on the stem diapsid *Hylonomus lyelli* (divergence between Eureptilia including Diapsida and Synapsida [35]). Three additional calibrations in Benton et al. [35] were ultimately excluded due to conflicts among node age priors during initial MrBayes runs: (i) Eulipotyphla: *Adunator ladae*, 61.6-164.6 Ma; (ii) Euarchontoglires: extinct primate sister taxa such as plesiadapids (e.g., *Paromomys farrandi*), 61.6-164.6 Ma; and (iii) Glires: *Mimotona lii*, 56-164.6 Ma. It appears those calibrations produced conflict with adjacent calibrations due to their extended maximum ages, which were set to the earliest eutherian *Juramaia* [40] given the associated stratocladistic uncertainty [35].

6.2 Tip-dating backbone analyses

For morphological data, we focused on the study of Zhou et al. [58], which consists of cladistic characters for extant and fossil mammals relevant to their interrelationships throughout the Mesozoic (66–252 million years, Ma). Previous studies have also re-analyzed this data set [59,60], and so served as comparisons for our work. Zhou et al. [58] originally had 84 fossil and 26 extant taxa that we trimmed to 76 fossil and 22 extant taxa. We removed 7 fossils allied to crown placentals (*Aspanlestes*, *Cimolestes*, *Eoungulatum*, *Glyptotherium*, *Gypsonictops*, *Leptictis*, and *Protungulatum*) and 1 taxon of unknown stratigraphic provenance (*Eleuterodon*). Removing crown fossils avoided complications with the subsequent union of backbone and patch clades (e.g., nesting *Glyptotherium* in the Xenarthra patch clade). However, we did retain *Obdurodon* within the monotreme crown given its uncontroversial placement. We also removed four extant taxa in Dasypodidae (*Chaetophractus*, *Euphractus*), Didelphidae (*Marmosa*), and Pseudocheiridae (*Pseudocheirus*) since those families were already sampled by other extant genera. We gathered stratigraphic ranges for sampled fossils from the Paleobiology Database (https://paleobiodb.org/; downloaded on 9 February 2016). This direct download of fossil age information provided a variety of stratigraphic sources and so usually gave wider age intervals than previously used (e.g., *Morganucodon* 190.8–208.5 Ma and *Juramaia* 150.8–167.7 Ma versus 199–203 Ma and fixed at 160 Ma, respectively, in Slater [59]). See S5 Data for full details of the age ranges and taxon sampling used in FBD analyses. Note that by following the topology of Zhou et al. [58] exactly, we constrained two shuotheriids (*Shuotherium* and *Pseudotribos*) to be paraphyletic due to an apparent error their matrix.

7. Construction of full dated mammalian phylogenies

There were three exceptions to the rescale-and-graft procedure described in the main text *Methods* section. First, the patch clades for Monotremata, Marsupialia, and Lagomorpha were re-scaled and attached at their inferred crown ages on the backbone (rather than the stem divergences based on their outgroups). This was because their outgroup relationships with *Rattus* (used as a genetically well-sampled representative of therians, placental, and rodents, respectively) were apparently too distantly related (>75 Ma) for accurate estimation of the stem branch length. Attaching via backbone crown ages was accomplished by keeping three extra taxa (a monotreme, marsupial, and rabbit) on the backbone during step '(ii)' above, using a relative scale of 1.0 for those three patches, and then pruning off the three extra taxa at the end.

Second, uncertainty in the sister relationships of Noctilionoidea, Vespertilionoidea, and Emballonuroidea (see above section on "Patch clade delimitation") required us to use the same distant outgroup for all three clades (*Pteropus alecto*, Pteropodidae). As a result, we added a step before '(iv) d' that determined which three-taxon topology was recovered in each of the 10,000 backbone trees, and then used that information to re-scale and attach those patch clades. While using the same outgroup is not ideal, it is a realistic outcome of backbone taxonomic uncertainty among major lineages of bats [20,61,62].

Third, uncertainty in the sister relationships of several other patch clades (e.g., Nesomyidae, Muridae), coupled with node age variation on the backbone, resulted in a small number of trees where the absolute scale of the patch's stem edge was longer (older) than the backbone tip edge for grafting. In these cases, which represented < 10% of trees, we used an arbitrarily short branch of length 0.000001 to graft those clades onto the backbone. The same solution was used in amphibians [63] and birds [64], where uncertainty of outgroup choice was more common and patch clades more numerous (84 and 129 patch clades, respectively). Because confidence in patch-clade and sister-taxon delimitation requires confidence in the backbone relationships of any major radiation, we expect these limits to this backbone-and-patch strategy of tree-building to persist, at least until disputed backbone nodes can be resolved or computational limits on patch clade size lifted.

Supplementary Results and Discussion

8. Supplemental comparisons to previous mammal studies

8.1 Backbone divergence times

Most node ages are statistically equivalent between our node- and tip-dated backbones, overlapping in their 95% highest posterior density (HPD) intervals (S4-S6 Figs). However, the substantially older root age for crown Mammalia in the tip-dated backbone (245.9 Ma, 222.1–268.3), and implausible overlap of this estimate with the Permian-Triassic extinction event [58], was one of several reasons that we chose to focus on results from the node-dated distribution of trees in our primary analyses. Furthermore, a few nodes are also significantly younger in the tip- than node-dated backbones: in rodents, we find that Muroidea is ~15 Ma younger (e.g., nodes 52, 53, and 54 in S6 Fig); and in yinpterochiropteran bats, node 39 is similarly young.

To examine how well our node-dating results compare to the fossil record, we synthesized information on the oldest fossil genera per extant mammalian order (S4 Table; data were gathered from the Paleobiology Database [65]). Fossil preservation waiting times (e.g., figure 1 in Brown et al. [66]), led us to expect phylogeny-based stem ages to be consistently older than the oldest fossil assigned to each extant order. We found broad agreement with this expectation, with maximum fossil ages overlapping the 95% HPD interval of stem age in all but one case (S4 Table). Our stem age for Didelphimorphia was ~18 Ma younger than the fossil *Pariadens* (93.5–105.3 Ma), which was assigned to "Family? Stagodontidae" by Cifelli [67] with no reference to Didelphimorphia. Stagodontidae was later added to Didelphimorphia by Davis [68] without reference to *Pariadens*, suggesting that it may instead attach to stem Marsupialia. *Eodelphis* would then be the oldest stem didelphimorphian (Stagodontidae; 66–72.1 Ma [68]), in line with our expectations of it being younger than our inferred stem age of 74.4 Ma (63.2, 87.1).

Supplementary Acknowledgments

9. Mammal artwork used in the manuscript

Figure 1

Monotremata	*Ornithorhynchus*	Web Dog; AussieIcons is licensed as Freeware, which means it is 100% free for both personal use and commercial use (https://www.fontspace.com/web-dog/aussieicons).
Paucituberculata	*Caenolestes*	Sarah Werning [phylopic.org, CC-BY4]
Didelphimorphia	*Didelphis*	Sarah Werning [phylopic.org, CC-BY4]
Microbiotheria	*Dromiciops*	Sarah Werning [phylopic.org, CC-BY4]
Notoryctemorphia	*Notoryctes*	Sarah Werning [phylopic.org, CC-BY4]
Dasyuromorphia	*Sminthopsis*	Sarah Werning [phylopic.org, CC-BY4]
Peramelemorphia	*Isoodon*	Steven Traver [phylopic.org, CC-BY4]
Diprodontia	*Phascolarctos*	Gavin Prideaux [phylopic.org, CC-BY4]
Diprodontia	*Macropus*	Web Dog; AussieIcons is licensed as Freeware, which means it is 100% free for both personal use and commercial use (https://www.fontspace.com/web-dog/aussieicons).
Cingulata	*Dasypus*	Steven Traver [phylopic.org, CC-BY4]
Pilosa	*Bradypus*	Sarah Werning [phylopic.org, CC-BY4]
Afrosoricida	*Tenrec*	Yan Wong [phylopic.org, CC-BY4]
Macroscelidea	*Petrodromus*	(uncredited) [phylopic.org, no copyright]
Tubulidentata	*Orycteropus*	Afrika Wildlife B Mammals2 is licensed as Freeware, which means it is 100% free for both personal use and commercial use (https://www.fontspace.com/fonts-of-afrika/afrika-wildlife-b-mammals2).
Hyracoidea	*Dendrohyrax*	Steven Traver [phylopic.org, CC-BY4]
Proboscidea	*Stegodon*	Zimices [phylopic.org, CC-BY4]
Sirenia	*Trichechus*	Iconian Fonts; Giants of the Sea is licensed as Freeware, commercial use requires donation, which means it is free for personal use only. To use the font commercially, you will need to purchase a license or contact Iconian Fonts (https://www.fontspace.com/iconian-fonts/giants-of-the-sea).
Camelidae	*Camelus*	Iconian Fonts; Zoologic is licensed as Freeware, commercial use requires donation, which means it is free for personal use only. To use the font commercially, you will need to purchase a license or contact Iconian Fonts (https://www.fontspace.com/iconian-fonts/zoologic).

Ruminantia	*Bos*	Cristopher Silva [phylopic.org, CC-BY4]
Ruminantia	*Giraffa*	Iconian Fonts; Zoologic is licensed as Freeware, commercial use requires donation, which means it is free for personal use only. To use the font commercially, you will need to purchase a license or contact Iconian Fonts (https://www.fontspace.com/iconian-fonts/zoologic).
Suina	*Sus*	Iconian Fonts; Zoologic is licensed as Freeware, commercial use requires donation, which means it is free for personal use only. To use the font commercially, you will need to purchase a license or contact Iconian Fonts (https://www.fontspace.com/iconian-fonts/zoologic).
Whippomorpha	*Hippopotamus*	Alan Carr; Animals is licensed as Freeware, which means it is 100% free for both personal use and commercial use (https://www.fontspace.com/alan-carr/animals).
Cetacea	*Balaenoptera*	Web Dog; AussieIcons is licensed as Freeware, which means it is 100% free for both personal use and commercial use (https://www.fontspace.com/web-dog/aussieicons).
Caniformes	*Vulpes*	Rebecca Groom [phylopic.org, CC-BY4]
Feliformes	*Leo*	Iconian Fonts; Zoologic is licensed as Freeware, commercial use requires donation, which means it is free for personal use only. To use the font commercially, you will need to purchase a license or contact Iconian Fonts (https://www.fontspace.com/iconian-fonts/zoologic).
Yangochiroptera	*Vespertilio*	Yan Wong [phylopic.org, CC-BY4]
Yinpterochiroptera	*Pteropus*	Bat Graphic #1705318 (License: Personal Use) (http://clipart-library.com/clipart/8TzKn5dac.htm).
Erinaceinae	*Erinaceus*	Claus Rebler [phylopic.org, CC-BY4]
Solenodontidae	*Solenodon*	T Michael Keesey after Monika Betley [phylopic.org, CC-BY4]
Soricidae	*Sorex*	Becky Barnes [phylopic.org, CC-BY4]
Talpidae	*Condylura*	WindWalker64; WWFurryFriends is licensed as Shareware, which means it is free for personal use only. To use the font commercially, you will need to purchase a license or contact WindWalker64 (https://www.fontspace.com/windwalker64/wwfurryfriends).
Perissodactyla	*Merychippus*	Mercedes Yrayzoz vectorized by T Michael Keesey [phylopic.org, CC-BY4]

Perissodactyla	*Rhinocerus*	Iconian Fonts; Zoologic is licensed as Freeware, commercial use requires donation, which means it is free for personal use only. To use the font commercially, you will need to purchase a license or contact Iconian Fonts (https://www.fontspace.com/iconian-fonts/zoologic).
Pholidota	*Manis*	Steven Traver [phylopic.org, CC-BY4]
Scandentia	*Ptilocercus*	T Michael Keesey after Joseph Wolf [phylopic.org, CC-BY4]
Dermoptera	*Volaticotherium*	Nobu Tamura vectorized by T Michael Keesey [phylopic.org, CC-BY4]
Catarrhini	*Homo*	T Michael Keesey [phylopic.org, CC-BY4]
Catarrhini	*Papio*	Owen Jones [phylopic.org, CC-BY4]
Platyrrhini	*Lagothrix*	(uncredited) [phylopic.org, no copyright]
Tarsiiformes	*Carlito*	Yan Wong [phylopic.org, CC-BY4]
Strepsirrhini	*Galago*	Joseph Wolf 1863 vectorization by Dinah Challen [phylopic.org, CC-BY4]
Lagomorpha	*Lepus*	Jan A Venter Herbert H T Prins David A Balfour Rob Slotow vectorized by T Michael Keesey [phylopic.org, CC-BY4]
Guinea_pig-related	*Erethizon*	Fonts of Afrika; Afrika Wildlife B Mammals2 is licensed as Freeware, which means it is 100% free for both personal use and commercial use (https://www.fontspace.com/fonts-of-afrika/afrika-wildlife-b-mammals2).
Mouse-related	*Rattus*	Rebecca Groom [phylopic.org, CC-BY4]
Squirrel-related	*Spermophilus*	Alan Carr; Animals is licensed as Freeware, which means it is 100% free for both personal use and commercial use (https://www.fontspace.com/alan-carr/animals).

Figure 2

Boreoeutheria	*Erethizon*	Afrika Wildlife B Mammals2 is licensed as Freeware, which means it is 100% free for both personal use and commercial use (https://www.fontspace.com/fonts-of-afrika/afrika-wildlife-b-mammals2).
Xenarthra	*Bradypus*	Sarah Werning [phylopic.org, CC-BY4]
Afrotheria	*Stegodon*	Zimices [phylopic.org, CC-BY4]

Figure 4

Monotremata	*Ornithorhynchus*	Web Dog; AussieIcons is licensed as Freeware, which means it is 100% free for both personal use and commercial use (https://www.fontspace.com/web-dog/aussieicons).
Paucituberculata	*Caenolestes*	Sarah Werning [phylopic.org, CC-BY4]

Didelphimorphia	*Didelphis*	Sarah Werning [phylopic.org, CC-BY4]
Microbiotheria	*Dromiciops*	Sarah Werning [phylopic.org, CC-BY4]
Notoryctemorphia	*Notoryctes*	Sarah Werning [phylopic.org, CC-BY4]
Dasyuromorphia	*Sminthopsis*	Sarah Werning [phylopic.org, CC-BY4]
Peramelemorphia	*Isoodon*	Steven Traver [phylopic.org, CC-BY4]
Diprodontia	*Macropus*	Web Dog; AussieIcons is licensed as Freeware, which means it is 100% free for both personal use and commercial use (https://www.fontspace.com/web-dog/aussieicons).
Cingulata	*Dasypus*	Steven Traver [phylopic.org, CC-BY4]
Pilosa	*Bradypus*	Sarah Werning [phylopic.org, CC-BY4]
Afrosoricida	*Tenrec*	Yan Wong [phylopic.org, CC-BY4]
Macroscelidea	*Petrodromus*	(uncredited) [phylopic.org, no copyright]
Tubulidentata	*Orycteropus*	Afrika Wildlife B Mammals2 is licensed as Freeware, which means it is 100% free for both personal use and commercial use (https://www.fontspace.com/fonts-of-afrika/afrika-wildlife-b-mammals2).
Hyracoidea	*Dendrohyrax*	Steven Traver [phylopic.org, CC-BY4]
Proboscidea	*Stegodon*	Zimices [phylopic.org, CC-BY4]
Sirenia	*Trichechus*	Iconian Fonts; Giants of the Sea is licensed as Freeware, commercial use requires donation, which means it is free for personal use only. To use the font commercially, you will need to purchase a license or contact Iconian Fonts (https://www.fontspace.com/iconian-fonts/giants-of-the-sea).
Carnivora	*Leo*	Iconian Fonts; Zoologic is licensed as Freeware, commercial use requires donation, which means it is free for personal use only. To use the font commercially, you will need to purchase a license or contact Iconian Fonts (https://www.fontspace.com/iconian-fonts/zoologic).
Eulipotyphla	*Solenodon*	T Michael Keesey after Monika Betley [phylopic.org, CC-BY4]
Pholidota	*Manis*	Steven Traver [phylopic.org, CC-BY4]
Artiodactyla	*Giraffa*	Iconian Fonts; Zoologic is licensed as Freeware, commercial use requires donation, which means it is free for personal use only. To use the font commercially, you will need to purchase a license or contact Iconian Fonts (https://www.fontspace.com/iconian-fonts/zoologic).
Cetacea	*Balaenoptera*	Web Dog; AussieIcons is licensed as Freeware, which means it is 100% free for both personal use and commercial use (https://www.fontspace.com/web-dog/aussieicons).

Yangochiroptera	*Vespertilio*	Yan Wong [phylopic.org, CC-BY4]
Perissodactyla	*Merychippus*	Mercedes Yrayzoz vectorized by T Michael Keesey [phylopic.org, CC-BY4]
Scandentia	*Ptilocercus*	T Michael Keesey after Joseph Wolf [phylopic.org, CC-BY4]
Dermoptera	*Volaticotherium*	Nobu Tamura vectorized by T Michael Keesey [phylopic.org, CC-BY4]
Primates	*Homo*	T Michael Keesey [phylopic.org, CC-BY4]
Lagomorpha	*Lepus*	Jan A Venter Herbert H T Prins David A Balfour Rob Slotow vectorized by T Michael Keesey [phylopic.org, CC-BY4]
Rodentia	*Erethizon*	Fonts of Afrika; Afrika Wildlife B Mammals2 is licensed as Freeware, which means it is 100% free for both personal use and commercial use (https://www.fontspace.com/fonts-of-afrika/afrika-wildlife-b-mammals2).

Figure 5

Rodentia	*Erethizon*	Afrika Wildlife B Mammals2 is licensed as Freeware, which means it is 100% free for both personal use and commercial use (https://www.fontspace.com/fonts-of-afrika/afrika-wildlife-b-mammals2).
Yangochiroptera	*Vespertilio*	Yan Wong [phylopic.org, CC-BY4]
Platyrrhini	*Lagothrix*	(uncredited) [phylopic.org, no copyright]

SUPPLEMENTARY REFERENCES

1. Ronquist F, Teslenko M, Mark P van der, Ayres DL, Darling A, Höhna S, et al. MrBayes 3.2: Efficient Bayesian Phylogenetic Inference and Model Choice across a Large Model Space. Syst Biol. 2012; sys029. doi:10.1093/sysbio/sys029
2. Huelsenbeck JP, Rannala B, Masly JP. Accommodating Phylogenetic Uncertainty in Evolutionary Studies. Science. 2000;288: 2349–2350. doi:10.1126/science.288.5475.2349
3. Huelsenbeck JP, B. Larget, R. E. Miller, F. Ronquist. Potential applications and pitfalls of Bayesian inference of phylogeny. Syst Biol. 2002;51: 673–688.
4. Douady CJ, Delsuc F, Boucher Y, Doolittle WF, Douzery EJP. Comparison of Bayesian and maximum likelihood bootstrap measures of phylogenetic reliability. Mol Biol Evol. 2003;20: 248–254.
5. Yang Z, Rannala B. Bayesian phylogenetic inference using DNA sequences: a Markov Chain Monte Carlo Method. Mol Biol Evol. 1997;14: 717–724. doi:10.1093/oxfordjournals.molbev.a025811
6. Degnan JH, Rosenberg NA. Gene tree discordance, phylogenetic inference and the multispecies coalescent. Trends Ecol Evol. 2009;24: 332–340. doi:10.1016/j.tree.2009.01.009
7. Heled J, Drummond AJ. Bayesian Inference of Species Trees from Multilocus Data. Mol Biol Evol. 2010;27: 570–580. doi:10.1093/molbev/msp274
8. Zimmermann T, Mirarab S, Warnow T. BBCA: Improving the scalability of *BEAST using random binning. BMC Genomics. 2014;15: S11. doi:10.1186/1471-2164-15-S6-S11
9. Mirarab S, Warnow T. ASTRAL-II: coalescent-based species tree estimation with many hundreds of taxa and thousands of genes. Bioinformatics. 2015;31: i44–i52. doi:10.1093/bioinformatics/btv234
10. Zhang C, Rabiee M, Sayyari E, Mirarab S. ASTRAL-III: polynomial time species tree reconstruction from partially resolved gene trees. BMC Bioinformatics. 2018;19: 153. doi:10.1186/s12859-018-2129-y
11. Burgin CJ, Colella JP, Kahn PL, Upham NS. How many species of mammals are there? J Mammal. 2018;99: 1–14. doi:10.1093/jmammal/gyx147
12. Mammal Diversity Database. American Society of Mammalogists, Mammal Diversity Database [Internet]. 2018 [cited 2 Jun 2018]. Available: https://mammaldiversity.org/
13. Faurby S, Svenning J-C. A species-level phylogeny of all extant and late Quaternary extinct mammals using a novel heuristic-hierarchical Bayesian approach. Mol Phylogenet Evol. 2015;84: 14–26. doi:10.1016/j.ympev.2014.11.001
14. Wilson DE, Reeder DM. Mammal species of the world: a taxonomic and geographic reference, 3rd ed. 3rd ed. Baltimore, MD: Johns Hopkins University Press; 2005.
15. IUCN. IUCN Redlist of Threatened Species, 2008 Assessments [Internet]. 2008.
16. Nilsson RH, Ryberg M, Kristiansson E, Abarenkov K, Larsson K-H, Kõljalg U. Taxonomic Reliability of DNA Sequences in Public Sequence Databases: A Fungal Perspective. PLoS ONE. 2006;1. doi:10.1371/journal.pone.0000059
17. Valkiūnas G, Atkinson CT, Bensch S, Sehgal RNM, Ricklefs RE. Parasite misidentifications in GenBank: how to minimize their number? Trends Parasitol. 2008;24: 247–248. doi:10.1016/j.pt.2008.03.004
18. Bridge PD, Roberts PJ, Spooner BM, Panchal G. On the unreliability of published DNA sequences. New Phytol. 2003;160: 43–48. doi:10.1046/j.1469-8137.2003.00861.x

19. Ranwez V, Harispe S, Delsuc F, Douzery EJP. MACSE: Multiple Alignment of Coding SEquences Accounting for Frameshifts and Stop Codons. PLOS ONE. 2011;6: e22594. doi:10.1371/journal.pone.0022594
20. Meredith RW, Janečka JE, Gatesy J, Ryder OA, Fisher CA, Teeling EC, et al. Impacts of the Cretaceous Terrestrial Revolution and KPg Extinction on Mammal Diversification. Science. 2011;334: 521–524. doi:10.1126/science.1211028
21. Katoh K, Standley DM. MAFFT Multiple Sequence Alignment Software Version 7: Improvements in Performance and Usability. Mol Biol Evol. 2013;30: 772–780. doi:10.1093/molbev/mst010
22. Löytynoja A. Phylogeny-aware alignment with PRANK. Methods Mol Biol Clifton NJ. 2014;1079: 155–170. doi:10.1007/978-1-62703-646-7_10
23. Capella-Gutiérrez S, Silla-Martínez JM, Gabaldón T. trimAl: a tool for automated alignment trimming in large-scale phylogenetic analyses. Bioinformatics. 2009;25: 1972–1973. doi:10.1093/bioinformatics/btp348
24. Stamatakis A. RAxML version 8: a tool for phylogenetic analysis and post-analysis of large phylogenies. Bioinformatics. 2014;30: 1312–1313. doi:10.1093/bioinformatics/btu033
25. Field DJ, Gauthier JA, King BL, Pisani D, Lyson TR, Peterson KJ. Toward consilience in reptile phylogeny: miRNAs support an archosaur, not lepidosaur, affinity for turtles. Evol Dev. 2014;16: 189–196. doi:10.1111/ede.12081
26. Wilkinson M. Majority-rule reduced consensus trees and their use in bootstrapping. Mol Biol Evol. 1996;13: 437–444.
27. Aberer AJ, Krompass D, Stamatakis A. Pruning Rogue Taxa Improves Phylogenetic Accuracy: An Efficient Algorithm and Webservice. Syst Biol. 2013;62: 162–166. doi:10.1093/sysbio/sys078
28. Sanderson MJ, Shaffer HB. Troubleshooting Molecular Phylogenetic Analyses. Annu Rev Ecol Syst. 2002;33: 49–72. doi:10.1146/annurev.ecolsys.33.010802.150509
29. Sanderson MJ, McMahon MM, Steel M. Terraces in phylogenetic tree space. Science. 2011;333: 448–450. doi:10.1126/science.1206357
30. R Core Team. R: A language and environment for statistical computing. Vienna, Austria; 2017.
31. Revell LJ. phytools: an R package for phylogenetic comparative biology (and other things). Methods Ecol Evol. 2012;3: 217–223.
32. Kearse M, Moir R, Wilson A, Stones-Havas S, Cheung M, Sturrock S, et al. Geneious Basic: an integrated and extendable desktop software platform for the organization and analysis of sequence data. Bioinforma Oxf Engl. 2012;28: 1647–1649. doi:10.1093/bioinformatics/bts199
33. Lanfear R, Calcott B, Ho SYW, Guindon S. PartitionFinder: Combined Selection of Partitioning Schemes and Substitution Models for Phylogenetic Analyses. Mol Biol Evol. 2012;29: 1695–1701. doi:10.1093/molbev/mss020
34. Lanfear R, Calcott B, Kainer D, Mayer C, Stamatakis A. Selecting optimal partitioning schemes for phylogenomic datasets. BMC Evol Biol. 2014;14: 82. doi:10.1186/1471-2148-14-82
35. Benton MJ, Donoghue PCJ, Asher RJ, Friedman M, Near TJ, Vinther J. Constraints on the timescale of animal evolutionary history. Palaeontol Electron. 2015;18.

36. Phillips MJ. Four mammal fossil calibrations: balancing competing palaeontological and molecular considerations. Palaeontol Electron. 2015;18: 1–16. doi:https://doi.org/10.26879/490
37. Parham JF, Donoghue PCJ, Bell CJ, Calway TD, Head JJ, Holroyd PA, et al. Best practices for justifying fossil calibrations. Syst Biol. 2012;61: 346–359.
38. Flynn JJ, Parrish JM, Rakotosamimanana B, Simpson WF, Wyss AR. A Middle Jurassic mammal from Madagascar. Nature. 1999;401: 57–60. doi:10.1038/43420
39. Luo Z-X, Kielan-Jaworowska Z, Cifelli RL. In quest for a phylogeny of Mesozoic mammals. Acta Palaeontol Pol. 2002;47: 1–78.
40. Luo Z-X, Yuan C-X, Meng Q-J, Ji Q. A Jurassic eutherian mammal and divergence of marsupials and placentals. Nature. 2011;476: 442–445. doi:10.1038/nature10291
41. Luo Z-X, Chen P, Li G, Chen M. A new eutriconodont mammal and evolutionary development in early mammals. Nature. 2007;446: 288–293. doi:10.1038/nature05627
42. Phillips MJ, Bennett TH, Lee MSY. Molecules, morphology, and ecology indicate a recent, amphibious ancestry for echidnas. Proc Natl Acad Sci. 2009;106: 17089–17094. doi:10.1073/pnas.0904649106
43. Beck RMD. An ‘ameridelphian’ marsupial from the early Eocene of Australia supports a complex model of Southern Hemisphere marsupial biogeography. Naturwissenschaften. 2012;99: 715–729. doi:10.1007/s00114-012-0953-x
44. Prideaux GJ, Warburton NM. An osteology-based appraisal of the phylogeny and evolution of kangaroos and wallabies (Macropodidae: Marsupialia). Zool J Linn Soc. 2010;159: 954–987. doi:10.1111/j.1096-3642.2009.00607.x
45. Fox RC, Scott CS, Rankin BD. New Early Carnivoran Specimens from the Puercan (Earliest Paleocene) of Saskatchewan, Canada. In: http://dx.doi.org/10.1666/09-165.1 [Internet]. 3 Nov 2010 [cited 13 Apr 2018]. doi:10.1666/09-165.1
46. Gheerbrant E. Paleocene emergence of elephant relatives and the rapid radiation of African ungulates. Proc Natl Acad Sci. 2009;106: 10717–10721. doi:10.1073/pnas.0900251106
47. Bergqvist LP, Abrantes E, Avilla L dos S. The Xenarthra (Mammalia) of São José de Itaboraí Basin (upper Paleocene, Itaboraian), Rio de Janeiro, Brazil. Geodiversitas. 2004;26: 323–337.
48. Gunnell GF, Simmons NB. Fossil Evidence and the Origin of Bats. J Mamm Evol. 2005;12: 209–246. doi:10.1007/s10914-005-6945-2
49. Rose KD, DeLeon VB, Missiaen P, Rana RS, Sahni A, Singh L, et al. Early Eocene lagomorph (Mammalia) from Western India and the early diversification of Lagomorpha. Proc R Soc Lond B Biol Sci. 2008;275: 1203–1208. doi:10.1098/rspb.2007.1661
50. Marivaux L, Vianey-Liaud M, Jaeger JJ. High-level phylogeny of early Tertiary rodents: dental evidence. Zool J Linn Soc. 2004;142: 105–134.
51. Antoine P-O, Marivaux L, Croft DA, Billet G, Ganerød M, Jaramillo C, et al. Middle Eocene rodents from Peruvian Amazonia reveal the pattern and timing of caviomorph origins and biogeography. Proc R Soc B-Biol Sci. 2012;279: 1319–1326.
52. Seiffert ER, Simons EL, Clyde WC, Rossie JB, Attia Y, Bown TM, et al. Basal Anthropoids from Egypt and the Antiquity of Africa’s Higher Primate Radiation. Science. 2005;310: 300–304. doi:10.1126/science.1116569
53. Seiffert ER, Simons EL, Attia Y. Fossil evidence for an ancient divergence of lorises and galagos. Nature. 2003;422: 421–424. doi:10.1038/nature01489

54. Seiffert ER. Revised age estimates for the later Paleogene mammal faunas of Egypt and Oman. Proc Natl Acad Sci. 2006;103: 5000–5005. doi:10.1073/pnas.0600689103
55. Bajpai S, Gingerich PD. A new Eocene archaeocete (Mammalia, Cetacea) from India and the time of origin of whales. Proc Natl Acad Sci. 1998;95: 15464–15468. doi:10.1073/pnas.95.26.15464
56. O'Leary MA, Uhen MD. The time of origin of whales and the role of behavioral changes in the terrestrial-aquatic transition. Paleobiology. 1999;25: 534–556. doi:10.1017/S0094837300020376
57. Wesley-Hunt GD, Flynn JJ. Phylogeny of the carnivora: Basal relationships among the carnivoramorphans, and assessment of the position of 'miacoidea' relative to carnivora. J Syst Palaeontol. 2005;3: 1–28. doi:10.1017/S1477201904001518
58. Zhou C-F, Wu S, Martin T, Luo Z-X. A Jurassic mammaliaform and the earliest mammalian evolutionary adaptations. Nature. 2013;500: 163–167. doi:10.1038/nature12429
59. Slater GJ. Phylogenetic evidence for a shift in the mode of mammalian body size evolution at the Cretaceous-Palaeogene boundary. Methods Ecol Evol. 2013;4: 734–744. doi:10.1111/2041-210X.12084
60. Close RA, Friedman M, Lloyd GT, Benson RBJ. Evidence for a Mid-Jurassic Adaptive Radiation in Mammals. Curr Biol. 2015;25: 2137–2142. doi:10.1016/j.cub.2015.06.047
61. Teeling EC, Jones G, Rossiter SJ. Phylogeny, Genes, and Hearing: Implications for the Evolution of Echolocation in Bats. Bat Bioacoustics. 2016; 25–54. doi:10.1007/978-1-4939-3527-7_2
62. Amador LI, Arévalo RLM, Almeida FC, Catalano SA, Giannini NP. Bat Systematics in the Light of Unconstrained Analyses of a Comprehensive Molecular Supermatrix. J Mamm Evol. 2018;25: 37–70. doi:10.1007/s10914-016-9363-8
63. Jetz W, Pyron RA. The interplay of past diversification and evolutionary isolation with present imperilment across the amphibian tree of life. Nat Ecol Evol. 2018; 1. doi:10.1038/s41559-018-0515-5
64. Jetz W, Thomas GH, Joy JB, Hartmann K, Mooers AO. The global diversity of birds in space and time. Nature. 2012;491: 444–448. doi:10.1038/nature11631
65. Alroy J, Uhen MD, Mannion PD, Jaramillo C, Carrano MT, van den Hoek Ostende LW. Taxonomic occurrences of Mammalia recorded in Fossilworks, the Evolution of Terrestrial Ecosystems database, and the Paleobiology Database. Fossilworks. http://fossilworks.org. 2018;
66. Brown JW, Rest JS, García-Moreno J, Sorenson MD, Mindell DP. Strong mitochondrial DNA support for a Cretaceous origin of modern avian lineages. BMC Biol. 2008;6: 6. doi:10.1186/1741-7007-6-6
67. Cifelli RL. Chapter 5: Marsupial Mammals from the Albian–Cenomanian (Early–Late Cretaceous) Boundary, Utah. Bull Am Mus Nat Hist. 2004;285: 62–79. doi:10.1206/0003-0090(2004)285<0062:C>2.0.CO;2
68. Davis BM. A revision of "pediomyid" marsupials from the Late Cretaceous of North America. ACTA Palaeontol Pol. 2007; 40.
69. Bininda-Emonds ORP, Cardillo M, Jones KE, MacPhee RDE, Beck RMD, Grenyer R, et al. The delayed rise of present-day mammals. Nature. 2007;446: 507–512. doi:10.1038/nature05634
70. dos Reis M, Inoue J, Hasegawa M, Asher RJ, Donoghue PCJ, Yang Z. Phylogenomic datasets provide both precision and accuracy in estimating the timescale of placental

mammal phylogeny. Proc R Soc Lond B Biol Sci. 2012;279: 3491–3500. doi:10.1098/rspb.2012.0683
71. Ronquist F, Lartillot N, Phillips MJ. Closing the gap between rocks and clocks using total-evidence dating. Phil Trans R Soc B. 2016;371: 20150136. doi:10.1098/rstb.2015.0136
72. Foley NM, Springer MS, Teeling EC. Mammal madness: is the mammal tree of life not yet resolved? Phil Trans R Soc B. 2016;371: 20150140. doi:10.1098/rstb.2015.0140

S3 Table.

Divergence times relative to prior studies. Crown divergence mean estimates (Est) and 95% confidence intervals (CI, lower and upper) for each taxon listed, with the 27 extant mammal orders in capital letters. Ages in gray are order-level divergences estimated near the Cretaceous-Paleogene (K-Pg) extinction, with "near" defined as having 95% CI < 3 Ma of 66 Ma, while black ages have CIs >3 before 66 Ma. Our node-dated estimates are compared to global amino acid and DNA dates [20], best estimate dates [69], combined 14K+Mit dates [70], rapid diversification posteriors [71], and fossil compendia [65,72]. Dates are missing if a node was not recovered or lacked taxon sampling.

		This study			Meredith et al. (2011)			Bininda-Emonds et al. (2007)			Dos Reis et al. (2012)			Ronquist et al. (2016)			Foley et al. (2016)		PaleoBioDB	
Taxon	N	Est	Low	Up	Est	Low	Up	Est	Low	Up	Est	Low	Up	Est	Low	Up	Fossil crown min	Fossil crown max	Fossil stem max	oldest genus/order
Mammalia	5911	188.4	166.7	210.9	217.8	203.3	238.2	166.2	Fixed	Fixed	184.7	174.6	191.9	.	.	.	164.9	227	.	.
MONOTREMATA	5	38.2	13.5	79.4	36.7	22.4	103.1	63.6	52.2	75	56.4	30.6	84.7	.	.	.	25	121	122.5	*Kryoryctes*
Theria	5906	159.6	156.3	166.2	190	167.2	215.3	147.4	141.8	153.1	172.8	168.5	177.9	.	.	.	.	.	.	.
Marsupialia	362	79.4	67.9	92.8	81.8	67.9	97.2	82.5	71.4	93.7	76.0	64.3	83.6	.	.	.	65.18	83.8	.	.
PAUCITUBERCULATA	7	16.4	12.3	20.3	11.7	7.2	16.2	33.2	26.3	40.1	27.8	11.0	47.0	.	.	.	.	.	59.0	*Bardalestes*
DIDELPHIMORPHIA	106	38.3	31.3	45.2	31.4	23	38.4	56.2	45.9	67.7	50.5	39.4	60.4	.	.	.	11.61	66	105.3	*Pariadens*
Australidelphia	100	63.4	54.1	74.8	64.2	53.7	75.5	66.8	63.8	74.2	70.2	58.7	76.6	.	.	.	.	.	.	.
MICROBIOTHERIA	1	.	.	.	.	.	.	.	.	.	.	.	.	.	.	.	.	.	66.0	*Khasia*
NOTORYCTEMORPHIA	2	6.1	3.6	9.0	.	.	.	9.3	8.3	10.2	.	.	.	.	.	.	.	.	23.0	*Naraboryctes*
DASYUROMORPHIA	78	34.1	27.7	40.9	30	22.1	41.7	31.3	16.2	55.8	46.0	35.6	54.9	.	.	.	15.97	54.65	58.7	*Gaylordia*
PERAMELEMORPHIA	22	29.5	24.1	35.2	28	21.1	37.1	36.2	30.6	41.8	40.9	30.8	51.4	.	.	.	4.36	54.65	28.4	*Galadi*
DIPROTODONTIA	146	48.7	41.1	57.5	52.8	42.4	64	54.1	52.4	55.8	57.5	46.5	64.9	.	.	.	24.7	54.65	33.9	*Cercartetus*
Placentalia	5544	91.8	77.4	105.0	101.3	92.1	116.8	98.5	93.2	108.1	89.2	87.9	90.4	85	76	93	65.2	131.5	.	.
Xenarthra	33	67.4	53.0	83.9	65.4	58.4	71.5	72.5	67.4	77.6	69.4	66.5	71.8	43	35	55	47.8	66	.	.
CINGULATA	21	37.6	27.7	47.9	.	.	.	.	.	.	.	.	.	.	.	.	.	.	66.0	*Proeuphractus*
PILOSA	12	59.8	46.8	75.3	56.4	49.2	62.9	.	.	.	62.7	57.5	66.9	.	.	.	31.5	66	58.7	*Asiabradypus*
Afrotheria	92	80.0	64.0	94.5	80.9	74.4	96.5	93.4	90.4	96.4	70.4	68.7	72.1	66	61	74	.	.	.	.
Afroinsectiphilia	75	77.5	62.4	92.3	78.6	71.8	95.2	93.2	90.2	96.2	64.7	61.2	67.9	56	45	66	.	.	.	.
AFROSORICIDA	55	70.4	56.5	85.8	68.2	56.8	88	85.2	81	89.3	.	.	.	.	.	.	.	.	63.3	*Eudaemonema*
MACROSCELIDEA	19	59.0	44.8	71.7	49.1	37.7	57.2	50.7	43.1	58.3	32.4	19.4	43.9	.	.	.	15.97	56	66.0	*Cingulodon*
TUBULIDENTATA	1	.	.	.	.	.	.	.	.	.	.	.	.	.	.	.	.	.	28.1	*Orycteropus*
Paenungulata	17	54.0	41.5	67.4	64.3	56	70.6	75.9	72.0	79.8	60.3	58.3	62.2	61	47	68	59.2	72.3	.	.

HYRACOIDEA	5	10.0	4.3	15.4	6.1	3.9	8.3	19.1	18.4	20	16.0	76	27.0	.	.	.	6.08	11.62	56.0	*Megalohyrax*
PROBOSCIDEA	7	10.1	4.8	16.2	5.3	1.8	8	19.5	7.6	31.4	8.4	3.4	15.9	.	.	.	6.8	11.62	59.2	*Eritherium*
SIRENIA	5	14.3	7.0	22.6	31.4	25	34.4	52.2	37.9	66.5	27.2	12.6	38.7	.	.	.	41.3	59.2	56.0	*Prorastomus*
Boreoeutheria	5419	83.5	73.1	94.8	92	82.9	107.6	96.1	92.9	98.4	83.8	82.8	84.9	81	74	90	.	.	.	.
Laurasiatheria	2456	75.1	66.3	84.1	84.6	78.5	93	87.8	85	90.5	76.0	74.9	77.0	75	68	82	.	.	.	.
EULIPOTYPHLA	491	74.3	65.4	83.6	77.3	70.7	85.8	82.5	79.8	85.3	61.5	60.9	62.0	56	44	69	61.6	83.8	61.7	*Litolestes*
Erinaceidae	24	46.7	39.1	53.7	38.9	29.0	48.9	65.5	60.9	70.1	48.2	43.1	52.7	.	.	.	.	.	.	.
Solenodontidae	3	10.9	0.2	39.6	---	---	---	40.8	40.8	40.8	---	---	---	.	.	.	.	.	.	.
Soricidae	420	47.2	39.5	55.2	---	---	---	49.0	40.3	58	33.2	26.5	39.6	.	.	.	.	.	.	.
Talpidae	44	40.6	32.8	47.7	---	---	---	61.8	52.3	71.3	34.5	29.5	39.4	.	.	.	.	.	.	.
PHOLIDOTA	8	27.1	17.9	38.2	25.3	16.9	35.7	19.1	7.3	46.9	.	.	.	.	.	.	.	.	48.6	*Cryptomanis*
CARNIVORA	298	40.2	33.9	47.9	54.7	47.4	60.6	63.4	59.8	67.1	54.2	52.3	56.0	44	35	61	38	66	66.0	*Pappictidops*
Caniformes	172	34.5	28.6	40.4	46.5	39.6	52.1	59.2	55.8	62.6	41.8	34.6	47.8	.	.	.	38	56	.	.
Feliformes	126	27.2	22.3	32.2	39.7	33.4	45.9	42.5	37.2	47.9	48.5	45.5	51.5	.	.	.	.	.	.	.
PERISSODACTYLA	24	38.7	32.6	45.0	56.8	55.1	61	55.8	51.1	61	52.6	41.8	61.0	.	.	.	55.5	61.6	59.2	*Paschatherium*
ARTIODACTYLA	348	58.4	50.3	67.1	65.4	62.3	68.5	70.7	67.6	73.7	61.4	60.7	62.3	54	47	60	52.5	66	66.0	*Basilosaurus*
Ruminantia	225	34.0	29.1	41.3	40.3	35.1	46.4	30.2	26.5	34.5	38.7	34.3	42.9	.	.	.	.	.	.	.
Whippomorpha	95	39.3	32.7	46.4	53.7	51.1	58.2	52.2	41.9	62.6	48.1	45.9	50.1	.	.	.	52.5	61.6	.	.
Cetacea	91	25.9	15.2	38.6	29.4	13.4	35.1	30.2	26.5	34.5	34.4	33.7	36	.	.	.	34	47.8	.	.
CHIROPTERA	1287	57.1	48.7	66.0	66.5	62.3	71.3	71.2	68	74.3	59.2	57.7	60.7	55	43	65	47.8	66	56.0	*Ageina*
Yangochiroptera	902	51.9	45.3	58.8	56.3	52.2	61.3	.	.	.	50.7	46.1	54.6	.	.	.	47.8	61.6	.	.
Yinpterochiroptera	385	50.5	41.4	60.3	62.9	58.6	66.8	.	.	.	53.4	49.2	56.5	.	.	.	.	.	.	.
Euarchontoglires	2963	77.0	67.8	87.4	83.3	74.1	97.8	91.8	90	93.8	75.7	74.8	76.7	76	69	85	.	.	.	.
Primatomorpha	480	70.4	61.7	79.8	82	73.7	97.4	88.5	85.9	91	74.1	73.2	75.1	65	58	73	65.2	83.8	.	.
SCANDENTIA	20	52.8	38.1	68.4	55.9	45	63.9	31.7	29.9	34.7	.	.	.	.	.	.	34	66	48.6	*Eodendrogale*
DERMOPTERA	2	9.6	3.3	16.5	7.4	4.5	13.2	15	10.2	19.9	.	.	.	.	.	.	.	.	63.3	*Elpidophorus*
PRIMATES	458	67.1	57.6	76.9	71.5	64.3	78.4	84.5	81.9	87.1	68.2	67.3	69.2	58	52	65	38	66	66.0	*Pandemonium*
Catarrhini	152	20.7	17.0	24.6	20.6	17.0	22.7	36.6	33.8	39.5	26.3	25.3	27.4	.	.	.	20.55	38	.	.
Platyrrhini	161	17.0	14.2	20.1	14.6	11.2	18.4	24.5	21.4	28.3	16.2	10.3	22.1	.	.	.	11.8	37.3	.	.
Strepsirrhini	135	49.6	42.2	57.0	55.1	50.2	58.7	75.5	71.3	79.6	55.1	53.4	56.8	.	.	.	38	56	.	.
Glires	2483	72.5	64.4	81.1	79.5	71.5	94.1	88.9	87.8	90.1	70.8	69.9	71.8	73	66	81	.	.	.	.

LAGOMORPHA	91	50.9	47.6	56.0	50.2	47.4	56.9	64.3	60	68.7	47.9	45.9	49.3	.	.	.	53.7	61.6	56.0	*Aktashmys*
RODENTIA	2392	67.9	60.5	75.2	69	64.1	74.8	82.8	80.2	85.4	64.4	63.5	65.3	67	61	73	56	66	58.7	*Asiaparamys*
Guinea_pig-related	304	61.7	53.0	69.3	61.1	56.1	68.3	56.5	52.0	68.5	32.3	19.5	43.2	.	.	.	40.94	56	.	.
Mouse-related	1768	65.0	58.4	71.6	65.1	60.7	70.0	81.0	78.4	84.5	60.0	55.1	56.7	.	.	.	54	66	.	.
Squirrel-related	320	49.8	42.1	57.4	60.5	55.5	64.2	80.1	78.2	81.1	52.8	42.7	58.4	.	.	.	47.8	61.6	.	.

S4 Table.

Fossil maximum stratigraphic ages per order relative to stem ages from our node-dated phylogeny (95% HPD age of 10,000 trees). Fossil occurrences (occs.) per extant mammalian order were gathered from the Paleobiology Database.

Order	Fossil occs. per order	Oldest fossil genus	Fossil max age	Phylogeny stem ages			Diff (tree max - fossil max)
				**Mean**	**Min**	**Max**	
MONOTREMATA	31	*Kryoryctes*	122.5	188.4	166.7	210.9	88.5
**Marsupialia**							
PAUCITUBERCULATA	70	*Bardalestes*	59.0	79.4	67.9	92.8	33.8
DIDELPHIMORPHIA	281	*Pariadens*	105.3	74.4	63.2	87.1	**-18.2**
MICROBIOTHERIA	36	*Khasia*	66.0	63.4	54.1	74.8	8.8
DIPROTODONTIA	1011	*Paljara*	33.9	61.1	51.7	71.9	38.0
NOTORYCTEMORPHIA	2	*Naraboryctes*	23.0	60.0	50.6	70.6	47.5
DASYUROMORPHIA	202	*Gaylordia*	58.7	58.6	49.4	68.9	10.2
PERAMELEMORPHIA	79	*Galadi*	28.4	58.6	49.4	68.9	40.5
**Placentalia**							
Xenarthra							
PILOSA (=Xenarthra)	1184	*Asiabradypus*	58.7	67.4	53.0	83.8	25.1
CINGULATA	608	*Proeuphractus*	66.0	67.4	53.0	83.8	17.8
Afrotheria							
AFROSORICIDA	192	*Eudaemonema*	63.3	74.8	61.0	90.8	27.5
MACROSCELIDEA	695	*Cingulodon*	66.0	74.8	61.0	90.8	24.8
TUBULIDENTATA	93	*Orycteropus*	28.1	77.5	62.4	92.3	64.2
SIRENIA	438	*Prorastomus*	56.0	54.0	41.5	67.3	11.3
PROBOSCIDEA	2665	*Eritherium*	59.2	50.2	38.5	63.8	4.6
HYRACOIDEA	176	*Megalohyrax*	56.0	50.2	38.5	63.8	7.8
Laurasiatheria							
EULIPOTYPHLA	1442	*Litolestes*	61.7	75.1	66.3	84.1	22.4
CHIROPTERA	770	*Ageina*	56.0	70.0	61.7	79.3	23.3
ARTIODACTYLA	16374	*Basilosaurus*	66.0	61.9	53.1	69.6	3.6
PERISSODACTYLA	8948	*Paschatherium*	59.2	61.9	53.1	69.6	10.4
CARNIVORA	8877	*Pappictidops*	66.0	60.6	52.6	69.6	3.6
PHOLIDOTA	16	*Cryptomanis*	48.6	60.6	52.6	69.6	21.0
Euarchontoglires							
PRIMATES	3181	*Pandemonium*	66.0	70.4	61.7	79.8	13.8
DERMOPTERA	29	*Elpidophorus*	63.3	60.9	50.1	72.4	9.1
SCANDENTIA	7	*Eodendrogale*	48.6	60.9	50.1	72.4	23.8
LAGOMORPHA	1971	*Aktashmys*	56.0	72.4	64.4	81.1	25.1
RODENTIA	15928	*Asiaparamys*	58.7	72.4	64.4	81.1	22.4

S5 Table.

Per clade summary of tip-level diversification rate (tip DR). Tip DR median, 95% confidence interval, and the skew in a given clade across 10,000 node-dated trees. Tests of the clade tip DR versus the (non-clade) background rate used the Mann-Whitney U statistic: greater (>, grayed), lesser (<), or not significant (NS).

Taxon	Richness	Median	Low (2.5%)	High (97.5%)	Skew	Mann-Whitney U-test	
Mammalia	5804	0.211	0.056	0.480	0.145	.	
MONOTREMATA	5	0.022	0.015	0.022	-1.200	***	<
RODENTIA	2354	0.208	0.061	0.482	1.193	NS	
Mouse-related	1742	0.203	0.060	0.430	0.726	*	<
Squirrel-related	320	0.213	0.076	0.501	0.835	NS	
Guinea pig-related	292	0.233	0.057	0.684	1.090	**	>
LAGOMORPHA	90	0.265	0.113	0.543	0.595	***	>
CHIROPTERA	1282	0.180	0.062	0.442	1.585	***	<
Yinpterochiroptera	381	0.225	0.072	0.647	1.216	**	>
Yangochiroptera	901	0.162	0.059	0.351	0.720	***	<
EULIPOTYPHLA	484	0.229	0.053	0.486	0.753	NS	
Soricidae	414	0.243	0.086	0.491	0.847	***	>
Talpidae	44	0.128	0.043	0.221	-0.003	***	<
Erinaceidae	24	0.113	0.033	0.133	-0.797	***	<
SCANDENTIA	20	0.110	0.022	0.137	-1.163	***	<
DERMOPTERA	2	.	.	.	.	.	
PRIMATES	450	0.326	0.093	0.480	-0.431	***	>
Simiiformes	309	0.345	0.183	0.500	-0.127	***	>
Catarrhini	149	0.346	0.174	0.511	-0.013	***	>
Platyrrhini	160	0.344	0.221	0.487	-0.211	***	>
Strepsirrhini	131	0.259	0.084	0.397	-0.194	**	>
PERISSODACTYLA	18	0.106	0.062	0.171	0.074	***	<
ARTIODACTYLA	338	0.248	0.079	0.537	0.432	***	>
Ruminantia	217	0.260	0.090	0.502	0.163	***	>
Whippomorpha	93	0.247	0.075	0.602	0.599	***	>
PHOLIDOTA	8	0.057	0.054	0.072	0.983	***	<
CARNIVORA	286	0.238	0.084	0.458	0.305	***	>
Feliformes	122	0.259	0.089	0.473	0.153	***	>
Caniformes	164	0.232	0.084	0.426	0.398	*	>
CINGULATA	21	0.068	0.047	0.092	-0.034	***	<
PILOSA	10	0.047	0.024	0.058	-0.742	***	<
AFROSORICIDA	55	0.082	0.032	0.149	0.189	***	<
MACROSCELIDEA	19	0.061	0.044	0.068	-0.630	***	<
TUBULIDENTATA	1	.	.	.	.	.	

PROBOSCIDEA	2	.	.	.	.	.	
HYRACOIDEA	5	0.063	0.056	0.063	-0.408	***	<
SIRENIA	4	.	.	.	.	.	
Marsupialia	350	0.207	0.055	0.414	0.752	NS	
DIPROTODONTIA	139	0.262	0.055	0.530	0.325	***	>
DIDELPHIMORPHIA	105	0.207	0.075	0.336	0.008	NS	
DASYUROMORPHIA	77	0.183	0.104	0.270	0.058	**	<
PERAMELEMORPHIA	19	0.189	0.102	0.215	-1.031	NS	
NOTORYCTEMORPHIA	2	.	.	.	.	.	
PAUCITUBERCULATA	7	0.084	0.070	0.099	-0.155	***	<
MICROBIOTHERIA	1	.	.	.	.	.	

*** P < 0.001; ** P < 0.01; * P < 0.05

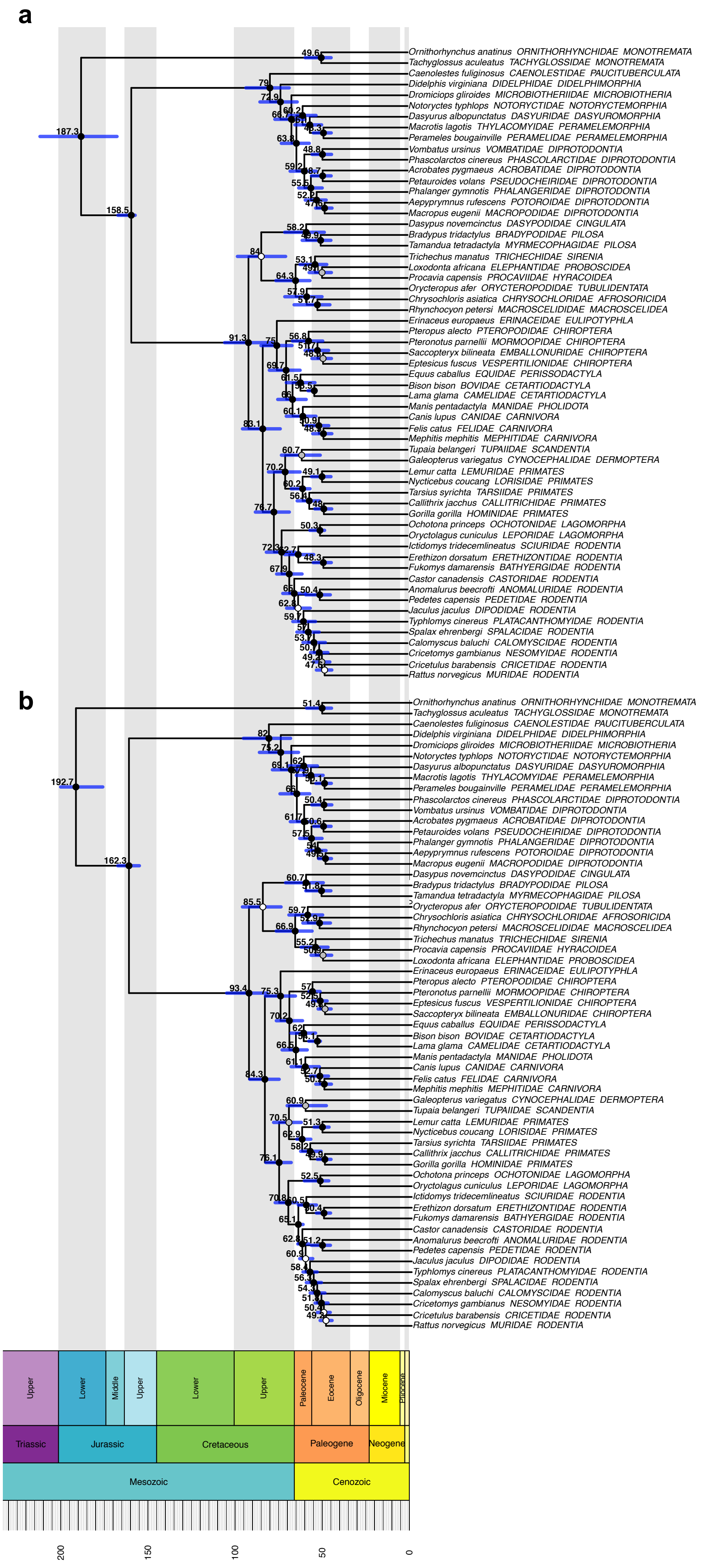

S4 Fig. Full node-dated backbone phylogenies. These were constructed using (**a**) exponential node priors (NDexp) or (**b**) uniform priors (NDuni) in MrBayes based on 17 fossil calibrations and molecular data from our 31-gene supermatrix. Topology is the maximum clade credibility tree of 10,000 phylogenies. Median ages and 95% highest posterior density intervals are displayed at nodes. Node circles indicate posterior probability (PP) values of ≥ 0.95 (black), 0.94-0.75 (grey), < 0.75 (white).

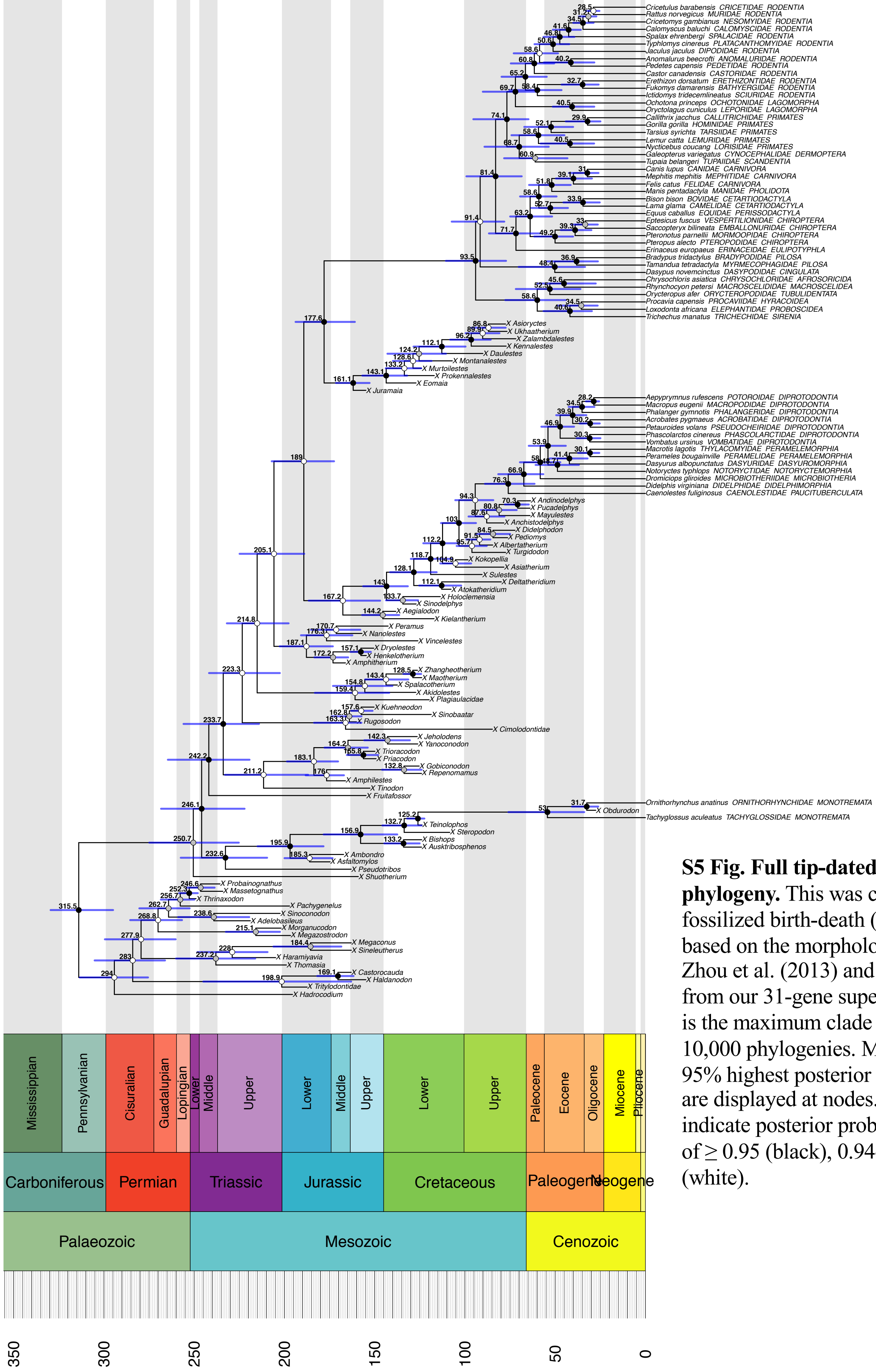

S5 Fig. Full tip-dated backbone phylogeny. This was constructed using fossilized birth-death (FBD) in MrBayes based on the morphological matrix of Zhou et al. (2013) and molecular data from our 31-gene supermatrix. Topology is the maximum clade credibility tree of 10,000 phylogenies. Median ages and 95% highest posterior density intervals are displayed at nodes. Node circles indicate posterior probability (PP) values of ≥ 0.95 (black), 0.94-0.75 (grey), < 0.75 (white).

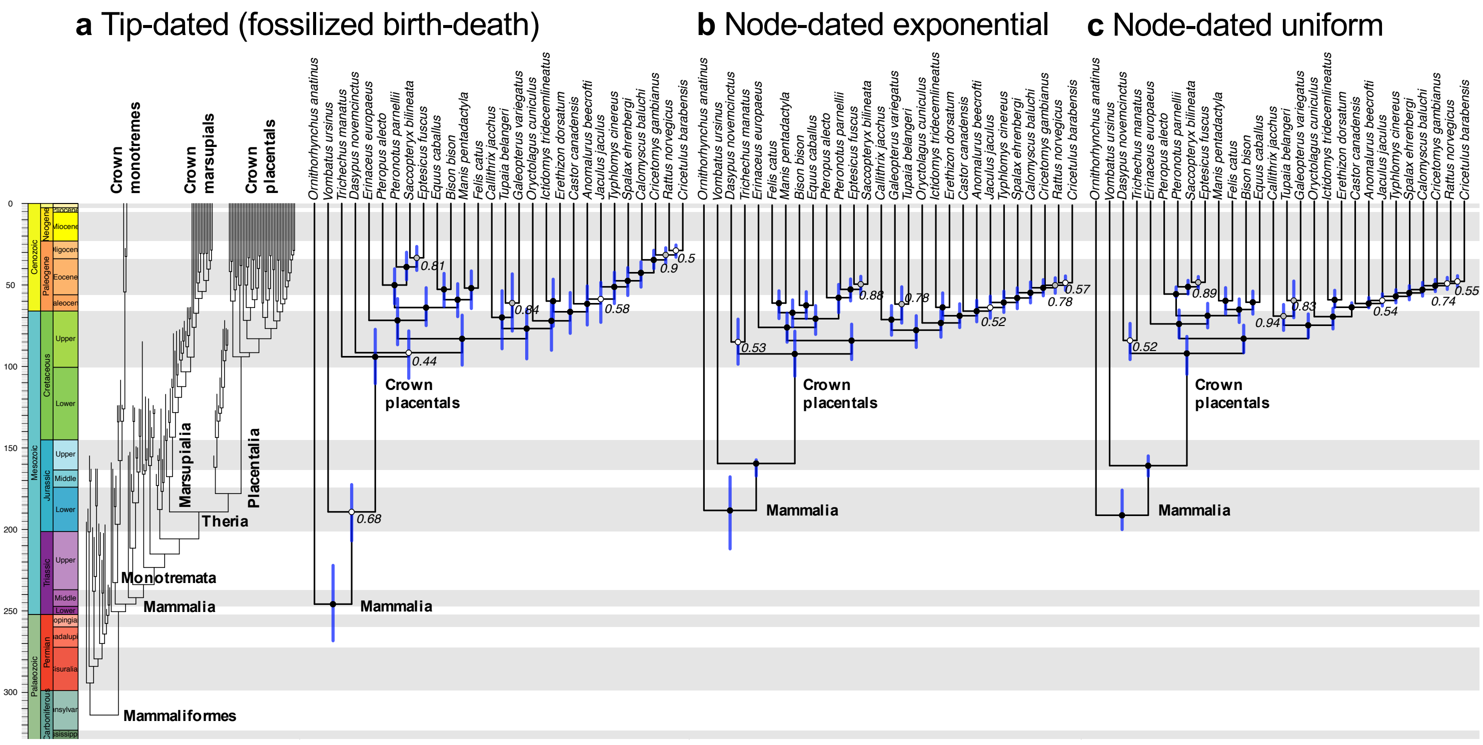

d Node age comparisons across the backbones pruned to 28 patch clade representatives

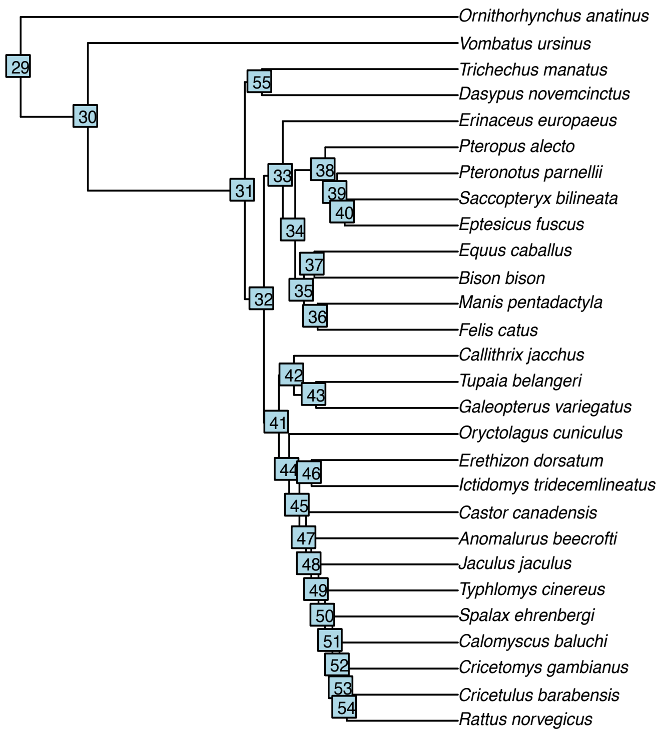

	Node-dated exponential				Node-dated uniform				Tip-dated (FBD)			
Node	Median	Low	Up	Width	Median	Low	Up	Width	Median	Low	Up	Width
29	187.3	166.7	210.9	44.2	192.7	177.3	201.5	24.2	246.1	222.1	268.3	46.3
30	158.5	156.3	166.2	9.9	162.3	156.3	168.7	12.4	189.0	172.5	207.0	34.5
31	91.3	77.4	105.0	27.6	93.4	83.0	106.2	23.2	93.5	77.2	110.5	33.3
32	83.1	73.1	94.7	21.7	84.3	76.1	92.9	16.8	81.4	68.5	99.2	30.6
33	75.0	66.3	84.1	17.8	75.3	66.7	83.9	17.2	71.7	58.4	86.7	28.3
34	69.7	61.7	79.3	17.7	70.2	62.7	77.7	15.0	63.2	51.7	74.9	23.3
35	66.0	58.2	74.5	16.3	66.5	60.0	74.4	14.5	58.6	49.2	69.5	20.2
36	60.1	52.6	69.6	17.0	61.1	53.0	69.2	16.2	51.8	41.4	64.5	23.1
37	61.5	53.1	69.6	16.4	62.0	55.2	69.3	14.1	52.7	42.9	63.2	20.3
38	56.8	48.7	66.0	17.4	57.0	52.6	58.9	6.3	49.2	40.1	61.6	21.5
39	51.7	45.3	58.8	13.5	52.5	48.6	56.9	8.4	39.3	29.9	46.9	17.0
40	48.5	43.7	54.9	11.2	49.8	46.3	53.7	7.5	33.0	26.4	41.1	14.7
41	76.7	67.8	87.3	19.5	76.1	69.2	83.7	14.5	74.1	64.8	95.3	30.6
42	70.2	61.7	79.8	18.2	70.5	63.4	79.3	15.9	68.7	53.6	89.2	35.6
43	60.7	50.1	72.4	22.3	60.9	49.0	71.5	22.5	60.9	43.2	78.3	35.1
44	72.3	64.4	81.1	16.6	70.8	65.7	78.4	12.7	69.7	59.9	90.1	30.1
45	67.9	60.5	75.1	14.6	65.1	62.4	66.0	3.6	65.2	54.7	79.7	25.0
46	62.7	53.9	70.5	16.6	60.5	54.7	65.1	10.4	58.4	46.5	75.3	28.8
47	65.0	58.4	71.6	13.3	62.8	59.1	65.4	6.3	60.8	50.6	74.6	24.0
48	62.8	55.8	69.4	13.6	60.9	56.8	64.3	7.5	58.6	48.4	73.0	24.6
49	59.7	52.8	65.8	13.0	58.4	54.3	62.4	8.1	50.6	42.2	61.4	19.3
50	57.0	50.7	63.5	12.8	56.3	51.8	60.5	8.7	46.8	39.3	56.5	17.1
51	53.7	47.2	60.0	12.9	54.3	49.8	58.3	8.5	41.6	35.8	51.3	15.5
52	50.7	45.7	55.7	10.0	51.8	48.0	55.5	7.4	34.5	28.7	40.0	11.3
53	49.2	44.6	54.1	9.5	50.4	46.8	54.2	7.4	31.2	27.2	36.8	9.6
54	47.6	43.5	52.3	8.9	49.2	45.7	52.6	6.9	28.5	25.5	33.0	7.5
55	84.0	70.0	97.6	27.7	85.5	75.0	97.2	22.2	---	---	---	---
Mean width of 95% HPD				17.0				12.5				24.1

S6 Fig. Comparison of results from three methods used to time-calibrate the backbone. Each method is pruned to the 28 patch clade representatives: (**a**) fossilized birth-death (FBD) where fossil taxa are placed as extinct tips in the tree (left side) and then pruned (right side); and node-dating (ND) approaches setting priors as (**b**) exponential priors from minimum to soft-max ages, and (**c**) uniform priors spanning minimum to maximum ages. Trees are maximum clade credibility summaries of 10,000 trees. Circles at nodes indicate posterior probability (PP) values of ≥ 0.95 (black), 0.94-0.75 (grey), < 0.75 (white), with the values < 0.95 given. (**d**) Inferred ages for backbone nodes are compared across methods, as based on the ND tree. Note that the FBD trees did not recover node 55 (see part **a** and S5 Fig).

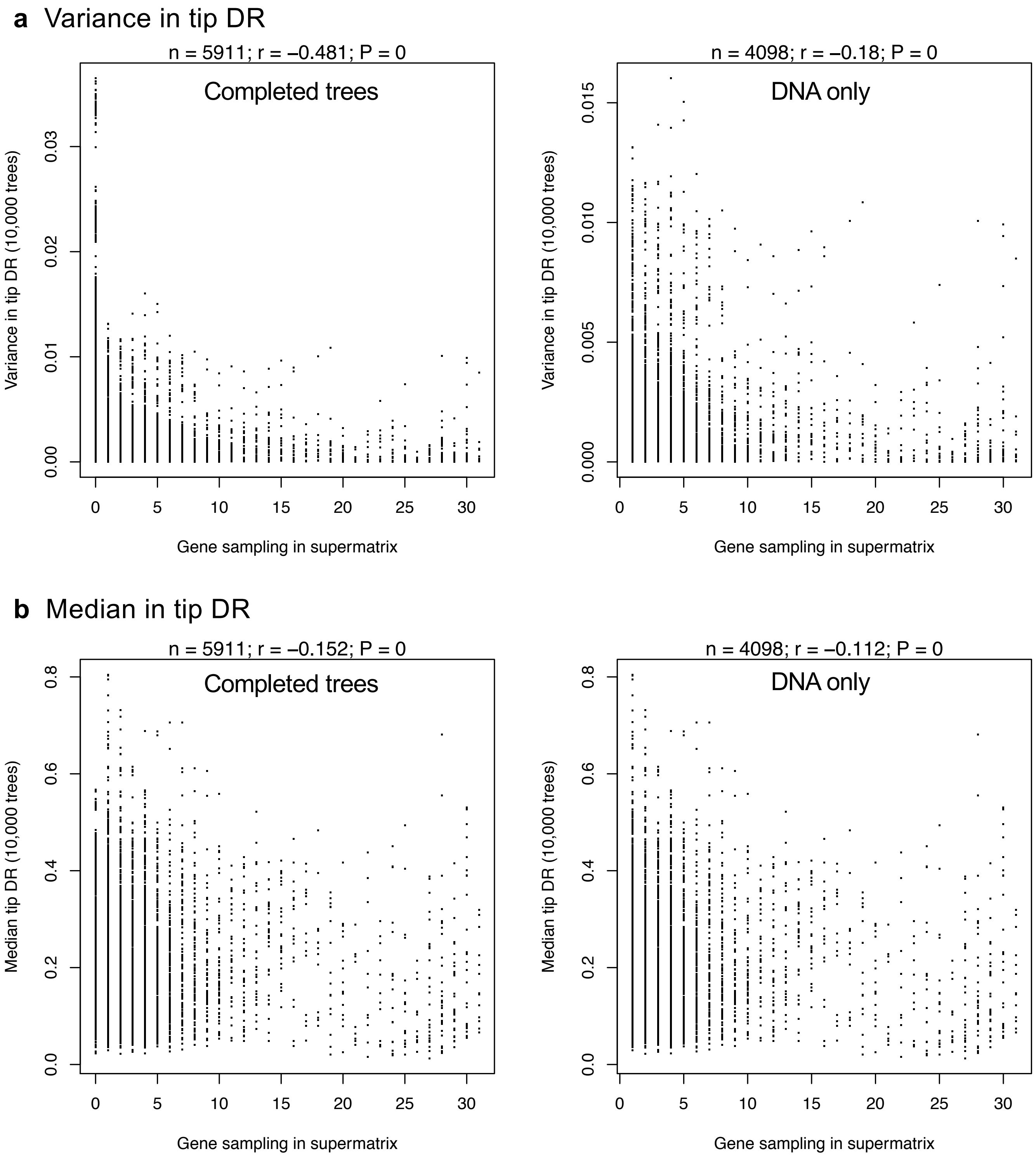

S7 Fig. Effect of gene sampling per species upon tip DR estimates. Compared are the per-species (a) variances and (b) medians in tip DR across 10,000 node-dated trees vs. the number of genes (0–31) sampled in the global DNA supermatrix. Completed trees are those where no-DNA species (0 genes) were added using PASTIS during MrBayes runs (see Supplementary Methods, section 5). As expected, variance in tip DR estimates is higher for completed species (note the different y-axes from left to right panel in part a). However, median tip DR estimates are similar between completed and DNA-only trees. Spearman's correlation coefficients, *r*, are shown for each plot as an indication of general trends in the data (slight negative trends do not account for phylogenetic covariance).

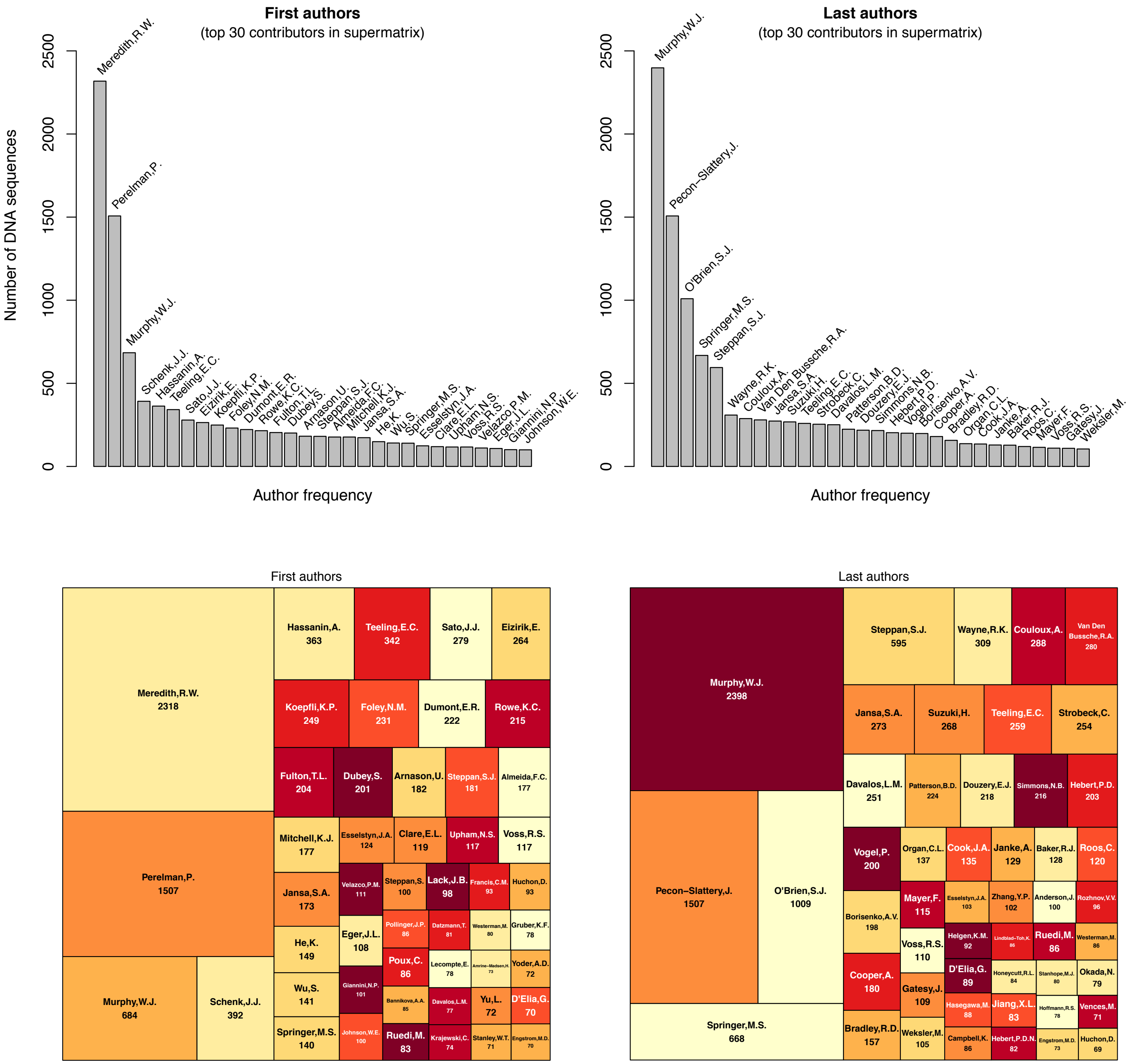

S8 Fig. Summary of data contributions per author on the NCBI public sequence database. The top 30 contributors as first and last authors toward the 31-gene supermatrix used in this study, first as barplots of author frequency (top row) and then treemap diagrams of the same data (bottom row). The full DNA supermatrix consisted of 21,021 total sequences after error-checking steps, of which 1,963 sequences were contributed by the Meredith et al. (2011) study that served as DNA baits for 27 of the 31 genes examined in this study.

Fig. S9

Node−dating exponential backbone −− Upham et al. 2019

MCC tree of DNA−only (4098 species) TopoFree distribution of 10,000 trees

Mean node ages; Bayesian PP >= 0.95 (black), < 0.95 (red)

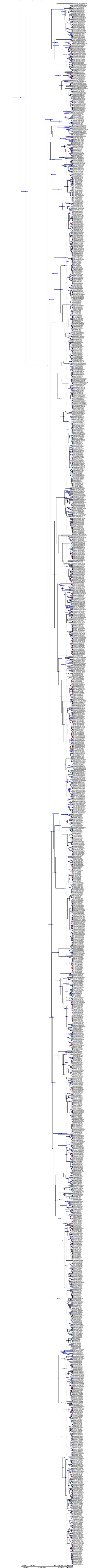

Fig. S10

Fossilized birth−death backbone (as Zhou et al. 2013) −− Upham et al. 2019

MCC tree of DNA−only (4098 species + 76 fossils) TopoFree distribution of 10,000 trees

Mean node ages; Bayesian PP >= 0.95 (black), < 0.95 (red)

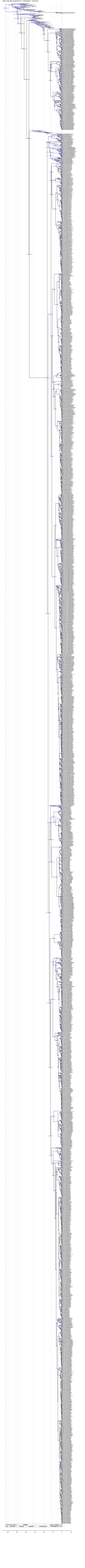

Supplement: S1 Text — Included are details of the taxonomic matchup, DNA sequence alignment, gene tree construction and error checking, final DNA sampling, 31-gene supermatrix analyses, patch clade run settings, 17 node-dated fossil calibrations, and construction of the full Mammalia-wide phylogenies. (PDF) [file pbio.3000494.s026.pdf]
