# Supplementary material for: Inferring the mammal tree: Species-level sets of phylogenies for questions in ecology, evolution, and conservation
Source: PLoS Biol. 2019 Dec 4;17(12):e3000494. doi: 10.1371/journal.pbio.3000494 (PMC6892540; doi:10.1371/journal.pbio.3000494)
Supplement: Attachment — Submitted filename: PLOSBiol_R1_responseToComments_22July2019.pdf [file pbio.3000494.s027.pdf]

Authors' response to editor and reviewer comments:

Dear Dr Upham,

Thank you very much for submitting your manuscript entitled "Ecological causes of uneven diversification and richness in the mammal tree of life" for review as a Research Article by PLOS Biology. As with all papers reviewed by the journal, yours was assessed and discussed by the PLOS Biology editors, by an academic editor with relevant expertise and in this case by three independent reviewers.

Based on the reviews, I regret that we will not be able to accept this manuscript for publication in the journal. I hope that you will find the reviewers' feedback helpful as you consider how to proceed with this work.

The rationale behind this decision is that the concerns about novelty of, and support for, the macroevolutionary inferences raise serious questions about the suitability of this aspect of your study for PLOS Biology. That said, it's possible that the mammalian phylogeny might in itself be appropriate for our new-ish Methods & Resource article type (i.e. as a "Resource"), after substantial revision to address the concerns raised.

Thus, if you are willing to take on the extra work involved, namely to remove the macroevolutionary (diversification) part of the paper, to comprehensively address the reviewers' concerns regarding the robustness and novelty of the phylogeny, and to resubmit as a Resource - and I must emphasize that this would be your choice - we would be willing to consider such an extensively revised version as a new submission. As such, the article would receive a new number and submission date and we would evaluate it against any work published in the interim that might undermine the novelty. Although we would try our best to engage the same academic editor and reviewers, please be aware that this isn't always possible. Also, if the new experiments and data introduce new areas that require review we might need to consult additional/new reviewers. The new data would all need to be evaluated as if a first submission and further revisions might prove necessary.

Response: Thanks for making this suggestion—Yes, we have decided to heed your advice and now present a fully revised manuscript that focuses solely on the Mammalia phylogeny component of our paper. By removing all of the macroevolutionary analyses, we have made room for additional explication of our tree-building methods, greater comparison of our approach with previous backbone-level and species-level studies of mammals, and discussion of the appropriate uses and limitations of our final tree products (four credible sets of 10,000 trees). Since the macroevolutionary analyses are now excluded from the present study, we only address reviewer comments below that pertain to our tree-building analyses.

We appreciate that the scale of the requested additional work is significant and you may well prefer to pursue publication of this work elsewhere. At this point, your manuscript is no longer under active consideration at PLOS Biology.

Please do feel free to contact me if you would like to discuss next steps for your manuscript. We

might also be able to facilitate a faster consideration at one of the other PLOS journals; if this is of interest to you, please let me know.

If you do make the choice to revise and re-submit to PLOS Biology, you should provide a point-by-point response to the reviewers' comments when re-submitting. Please include this as a separate file, labelled 'Response to Reviewers'. Please also provide the tracking number of this manuscript [PBIOLOGY-D-19-00877R1] in the 'previous interaction' section of the online submission form and in your cover letter when re-submitting.

Sincerely,

Roli Roberts

Roland G Roberts, PhD,
Senior Editor
PLOS Biology

REVIEWERS' COMMENTS:

Reviewer #1:

In this manuscript, the authors generate a new time-calibrated, species-level phylogeny of mammals and use it to perform macroevolutionary analyses. For example, they conclude that diversification is positively related to diurnality but not to latitude.

Overall, I do not think that this manuscript is appropriate for PLoS Biology. Although the authors only rarely acknowledge it, many of the same results they describe here were already found in earlier studies. Thus, the paper is substantially less novel than what one might infer from reading it. I find it very hard to believe that these authors are truly so unaware of the relevant literature on mammal phylogeny and macroevolution. Instead, it appears that the authors deliberately ignored previous studies to make theirs seem more novel.

Here are some examples of this (and other) problems.

1) The authors treat the lack of a relationship between latitude and diversification as the surprising new result of the study. This was also found by Soria-Carrasco & Castresana (2012: Proc. R. Soc. Lond.), analyzing rates among genera. On the other hand, Rolland et al. (2014; PLoS Biology) found increased diversification rates in tropical mammals using an SSE approach within orders. Remarkably, the authors of the present paper do not cite either of these papers, and do not address why their results are different. Similarly, the idea that speciation and extinction rates are higher at higher latitudes in mammals was found Weir & Schluter (2007; Science). What is particularly irritating is that they seem to present the basic conclusions of Weir & Schluter (2007) as if they were some novel conceptual conclusion of their own (in the Abstract!). Similarly, the finding that diurnality increases diversification rates was found by

Anderson & Wiens (2017; Evolution). That paper also is not cited.

Response R1.1: We thank the reviewer for their feedback. The founding assumption of our previous joint 'tree & macroevolution' manuscript was that existing supertree-based mammal trees were inappropriate for questions of diversification rates—thus, we did not previously discuss all literature on these topics. Admittedly, our assumption was premature. Now that we have split apart the 'tree' and 'macroevolution' components of our paper, there is more room to fully explain the justification for our improved mammal phylogeny, as well as compare it to previous studies. We ask that R1 please re-consider our manuscript in light of the new phylogeny-only focus and extensive comparison to previous works (Figs. 4-6).

2) Another major conclusion of the present study, that the species richness of mammalian clades is related to their diversification rates, was previously found by Castro-Insua et al. (2018; Scientific Reports). That paper is also not cited. The authors of that paper also found that diversification rates were not strongly related to the climates where these clades occur (this is further evidence against a relationship between climate and diversification, a finding that the authors here present as a novel conclusion of their study). In contrast, those authors did find a strong relationship between diversification rates and rates of climatic-niche divergence, a variable that the authors of this paper fail to consider. The authors also seem to ignore the various papers on mammal diversity and diversification by Machac, Graham, and Stork (e.g. Machac and Graham 2018; Am. Nat.; Machac et al. 2018; Global Ecology and Biogeography). I was also very surprised that the prominent study of mammalian diversification and diet was ignored (Price et al. 2012; PNAS).

Response R1.2: Not applicable to the new phylogeny-only focus.

3) The authors seem to justify ignoring the results of previous studies because they did not use the latest phylogeny. However, the authors present no evidence that changing the phylogeny will actually change the macroevolutionary conclusions (they state this without supporting citations, but this is an assumption that seems self-serving at best; see also #7 below). What is also problematic is that since they are changing the methods as well as changing the tree, it is difficult to isolate the specific effects of changing the phylogeny.

Response R1.3: Not applicable to the new phylogeny-only focus.

4) Outside of these generally less-than-novel macroevolutionary inferences, the main selling point of this study is the new mammalian phylogeny. However, the authors do not really address to what extent their tree is different from previous estimates. For example, Rolland et al. (2014: PLoS Biology) based their phylogeny on that of Meredith et al. (2011). Are the ordinal-level and family-level relationships the same here, or different? If they are different, how do we know that their estimate of higher-level relationships is any better than previous estimates? Is it more strongly supported, or less? The lack of comparison raises the possibility that there are only trivial differences between this tree and previous estimates, and that this new tree might even be a worse estimate than previous ones.

Response R1.4: We thank the reviewer for suggesting that greater emphasis be placed on comparing our mammal phylogenies to previous studies. As a result, we have added backbone-level comparisons to divergence times in our study to four previous estimates and the fossil record (Fig. 4) and species-level comparisons to three existing mammal supertrees (Fig. 5 and 6). We emphasize that our focus in building these phylogenies is improving the modeling of lineage diversification processes in mammals from root to tip across all ~6000 species. Thus, we also focus our comparisons to previous studies upon divergence times, tree shape, and the tempo of lineage diversification relative to our credible sets of trees—the topology of specific nodes is of secondary importance. Nevertheless, we include species-level maximum clade credibility (MCC) trees of the DNA-only data sets in the *Supporting Information* (Fig. S8 for node-dating, Fig. S9 for tip-dating).

5) I have numerous concerns about the methods. For example, the authors seem to be arguing that they can ignore controversies about macroevolutionary methods because they use multiple methods. Although this sounds reassuring in theory, in practice, it appears that each conclusion is generally based on a single method. Thus, rate shifts are primarily identified using BAMM, despite evidence that BAMM increasingly underestimates rate shifts as heterogeneity in rates increases (Rabosky 2014; Meyer and Wiens 2018), and has relatively limited ability to detect rate differences among clades (Kodandaramaiah & Murali 2018; PeerJ). I did not see evidence presented that BAMM and the DR statistic yielded congruent rate estimates. Similarly, the authors use the approach of Morlon et al. (2011; PNAS) to analyze diversification dynamics within clades, despite serious concerns raised about this approach by Burin et al. (2018; Systematic Biology; also not cited).

Response R1.5: In our revised phylogeny-focused manuscript, we added this sentence to the Discussion regarding our use of tip DR to compare species-level studies (line 877-880): "We use the tip DR metric (Jetz et al. 2012) because it is readily calculable across all 10,000 trees in our credible sets while being highly correlated with model-based estimators of tip speciation rates (demonstrated in Quintero and Jetz (2018) and reviewed in Title and Rabosky (2019))."

6) I was surprised to see the authors conclude that the level of missing data in their matrix (88%) was a level not expected to confound analyses. I could not find this in the papers that they cited. As far as I could tell, those papers did not identify a specific cut-off for a level of missing data that would or would not be problematic.

Response R1.6: We added a section on 'Limitations' in the Discussion (line 950-1010), which specifically deals with missing gene-by-species data in our DNA supermatrix:

> "The substantial level of missing data in our 31-gene supermatrix (mean = 88.1% per species) is worth further attention. Some simulation studies suggest that analyzing matrices with missing cells may yield erroneous estimates of topology, node support, and phylogeny branch lengths (e.g., (Lemmon et al. 2009)), while other empirical and simulation studies have found no or small impact of missing data (Wiens 2003; Wiens and Morrill 2011; Pyron et al. 2013; Roure et al. 2013). Wiens and Tiu (2012) demonstrated that adding taxa with 90% missing data is beneficial to phylogenetic analyses when the alternative is to be misled by incomplete taxon sampling. Instead,

model misspecification appears to have a greater impact on tree accuracy than missing data (Roure et al. 2013).

To empirically test this dynamic in our data, we performed a test of terminal branch length in the global ML tree relative to proportional DNA completeness (bp of sampled data per species / 39,099 bp of complete data). We found no relationship (spearman's r = -0.01, P = 0.582), corroborating the result of Pyron et al. (2013) to suggest that estimated branch lengths are not consistently biased by missing data. We note, however, that global biases in species distributional knowledge (e.g., Meyer et al. 2015) may also influence levels of taxonomic attention and thus DNA completeness. Subsequent tests should endeavor to include ecological covariates of missing data to tease apart their relative impacts on phylogenetic estimates."

7) Please look at lines 66–68 and also 69–70. This first statement is not supported by any citations, and may well be untrue. How do you know that an approximate estimate of the evolutionary timescale is not adequate? Indeed, I seriously doubt that the phylogeny in the present study is perfect. For example, it is based on a relatively small number of genes, whereas whole genomes are now available for many mammalian groups. Given that the tree presented by these authors is not perfect, and that better ones should soon be available, then what is the point of publishing the present study? Basically, if one follows the philosophy of these authors to its logical conclusion, then the macroevolutionary inferences of the present study are also based on an imperfect tree and should therefore be ignored.

Response R1.7: These sentences are now excluded from the revised phylogeny-focused version of our manuscript. Nevertheless, we want to thank this reviewer for pointing out our need to better explain the motivation for the present study. The reviewer's statement regarding their "doubt that the phylogeny in the present study is perfect" points out a widespread misconception in the phylogenetic research community that our study aims to address. That is, because no reconstructed phylogeny is ever expected to be perfect, single 'best' trees should not be the targeted output of phylogenetic studies. Instead, the aim should be to *estimate the probability* of the true tree, given the data, understanding that the true tree itself is likely impossible to obtain.

Thus, our entire study is motivated by the goal of *approximating the true history* with the full envelope of statistical uncertainty associated with our phylogenetic data. That is why we emphasize the use of samples of 100 or 1000 trees from our credible sets of 10,000 trees. Analyzing a single phylogeny as known without error is not expected to be sufficient, and can be misleading in many circumstances. Bayesian phylogenetics is founded on these principles. Papers by Huelsenbeck et al. (2000 Science 288:2349–2350) and Pagel and Lutzoni (2002 Pp. 148–161 in Biological Evolution and Statistical Physics, Springer) clearly outline these principles. However, given this reviewer's criticism, we have added sections in our revised Introduction to clarify the goals of this present study (see line 70-112).

Reviewer #2:

This is a very interesting MS that using cutting-edge techniques to produce the newest comprehensive time tree of extant mammals. The time tree then serves as a base to conduct a series of analyses to determine where variation in diversification rate have occurred and what factors might have been driving these differences.

As well done as the study is, the authors tend to overstate the novelty of their tree and the inferred divergence times and probably the robustness of the sensitivity analyses that were performed, although this latter aspect is difficult to judge. Given the number of species involved, Figure 1 is naturally unreadable WRT the phylogenetic relationships and a presentation of the higher-level relationships, where most of the "important" disagreement tends to lie, would be very helpful in judging what difference the new methods have really made. This is, to my knowledge, the largest phylogeny of mammals based exclusively on DNA data. Other comparable phylogenies in terms of size had to resort to supertree methods and in terms of data were much, much smaller. Given the push the authors make for their backbone-and-patch method (especially in the SI), together with their critique of the supertree method, some comparison or at least some data to be able to make a comparison seems to be a requirement.

Response R2.1: Given that length requirements are no longer as serious an issue as they were for our initial PLOS Biology Research Article submission, we now have the ability to include additional figures to document the higher level relationships in our tree. Our new Fig 2b and c now clearly shows the backbone-level relationships among the 28 patch clades that we incorporated in our trees, contrasting the topology and node ages derived from tip-dating and node-dating methods, respectively. New Fig 2d also clearly shows that the Placentalia basal relationships are not resolved in our study, but rather that uncertainty is propagated into the final samples of trees. The Boreoeutheria rooting (Atlantogenata hypothesis) is contrasted in blue versus to the Afrotheria rooting in red in that Fig. 2d. Furthermore, Figs. 3-6 make further comparisons within our study and between previous studies of mammalian phylogeny. We additionally added a section in the Discussion ('Backbone-level topology') for comparing our results to previous studies for four particularly controversial nodes.

In the same vein, it is necessary to indicate how robust the topology itself is, critical information that I could not find anywhere. For instance, the root of the Placentalia has been notoriously difficult to pin down, even with molecular data. Notwithstanding the question of what topology the authors found, how much support for it was there?

Response R2.2: This information is now provided in Fig. 2, as mentioned above, as well as in the *Supporting Information* Figs. S3-S5 with greater detail. Species-level maximum clade credibility (MCC) trees of the DNA-only data sets are shown at the end of the *Supporting Information* (Fig. S8 for node-dating, Fig. S9 for tip-dating).

What I am mostly concerned about here is the taxonomic placement of species without any DNA data, a procedure that is necessary to derive the complete tree needed for the diversification analyses. At best, I see this procedure as a necessary evil. More honestly, I am deeply skeptical of it because it amounts to a guess and one often based on questionable data. As the authors themselves noted when tracking down the species names, there were a significant number of instances where (poorly known) species had changed genera, a not insignificant change.

Response R2.3: We appreciate the reviewer's concern here, which is why we originally spent considerable effort performing taxonomic updates across Mammalia and unifying GenBank data

to accepted names with as great of rigor as feasible. We want all the DNA-missing species to be placed within their taxonomically-defined genus (if sampled for DNA, or else family), and thus we rely on the most up-to-date taxonomic understanding for these species. Our study aims to be a synthesis of current knowledge, but we acknowledge that gaps in biodiversity information will bias our study in some cases. The goal of this synthesis is to understand where these gaps are, and to thus to aid future phylogenetic research on these 'genetically missing' taxa.

More practically, in the current context, Bayesian analyses routinely return very high support values, leading me to suspect that much of the variation might derive from the taxonomically placed species. This in combination with the number of these latter species (ca. 1000), means that most of the 10 000 trees for the sensitivity analyses will be due to different placements of poorly known taxa rather than reflecting uncertainty in the DNA data. Unfortunately, there are no data provided to be able to assess this very important question upon which the remainder of the paper rests.

Response R2.4: The reviewer here refers to the sensitivity analyses for macroevolutionary tests of tip DR and ecological traits, which we conducted in the previous version of the manuscript but are now excluded in this phylogeny-only refocus. Nevertheless, we concur that much of the variation in per-species tip DR values is due to the imputation of DNA-missing species using taxonomic constraints – our current Fig 3 and 6 show the 95% CIs of the tip DR values. However, this is a statistically honest representation of our understanding about tip-level rates of speciation in these species. Tip rates require complete sampling of modern species, so taxonomic imputation will be needed (indeed, a 'necessary evil') until we can sample all living mammals for one or more genes.

The authors do, however, provide a good comparison of divergence-time estimates in the SI, something that is very welcome. However, as the authors very briefly state there, their inferred crown ages for the different clades were broadly similar to previous studies, which, unfortunately, undermines their contention of both the superiority of their method and the novelty of their results, at least with respective to the divergence times upon which the diversification analyses strongly depend.

Response R2.5: The higher-level divergence-time estimates in our study are based on many of the same fossil calibrations as used in previous studies – indeed, our goal was to use the cladistically most securely placed fossils, which by definition are derived from previous studies. So the concordance of our divergence-time estimates with previous studies is indeed a strength, not a weakness. A further strength – and the key novelty – of our study is that those backbone-level calibrations are propagated all the way to the species-level throughout the entire Mammalia radiation. As a result, the species-level branches are time-scaled in a comparable manner across the entire tree. Clades from very disparate parts of the tree can now be pruned off and compared directly, without worry regarding differences in the underlying data, assumptions, or models of evolution used to calculate their branch lengths. This is the core strength of our study, which sets in apart from previous species-level studies of mammals.

In the end, I find it disappointing that no real comparison to other studies have really been made and the few that have been made are buried in the SI, showing either no broad similarity

(divergence times) or substantial differences (tip DR). However, substantial differences, even when italicised by the authors, does not automatically mean substantial improvement, just different.

Response R2.6: We added a range of comparisons to previous studies of mammalian phylogeny in our revised manuscript (Fig. 4-6). The previous focus on the macroevolutionary analyses had not previously enabled many of these comparisons, so we hope that their present inclusion is more satisfying to this reviewer.

By extension, I feel that the authors need to conduct a more meaningful sensitivity analysis at least with respect to the topology. As they note in the SI, competing methods like ASTRAL II are still too slow to analyse the data set; however, ASTRAL III has been available since May 2018 and reportedly scales up to 10 000 species. Together with sensitivity analyses that are not driven so much by taxonomically placed taxa, obtaining a topology using ASTRAL III would be a much more meaningful test of the robustness of the patterns found in this study. If the results are largely specific to the essentially single tree topology found here (i.e., if most of the topological variation does come from non-DNA species), then they will stand or fall with the next phylogeny that inevitably will come out. By contrast, highlighting those patterns that are robust to topological changes would be a much bigger step forward.

Response R2.6: We added the following section to the Methods (section for 'Global RAxML tree' line 323-330) with regard to ASTRAL-III:

> "While methods based on the multi-species coalescent (MSC) are preferable to supermatrix analyses in terms of their modeling of gene tree to species tree processes at both deep and shallow nodes (Liu et al. 2019), most software implementations of these methods are computationally infeasible for large trees. For example, fully Bayesian implementations using *BEAST do not scale well beyond a few hundred species (Ogilvie et al. 2016). We note that ASTRAL-III (Zhang et al. 2018) claims to scale to 10,000 species, but its release in May 2018 after the majority of our study was complete prevented tests using our dataset."

Some more minor and/or specific points:

L138: The possible non-effect of the K-Pg boundary on mammalian diversification was also noted by the Bininda-Emonds et al. supertree study.

Response R2.7: We removed the macroevolutionary analyses from the current ms.

L142: Although there has been much disagreement about deeper mammalian divergence times, pretty much everyone agrees that the majority of the mammalian radiation has occurred in the past 50 million years. This is not a new result.

Response R2.8: We removed the macroevolutionary analyses from the current ms.

L224: I am always concerned with phylogenetic reconstructions of latitude for a pair of reasons. The first is the assumption that we can estimate it for ancestors correctly. All estimates /

extrapolations lose accuracy the further they are from the data and my feeling is that latitude would be worse than many other variables in this regard. Notwithstanding these problems, the second is that such estimates never account for the changes from continental drift, meaning that even if a lineage has never moved its continental location, it could still find itself in different latitudes over an extended time period because the continents are moving. For example, a reconstruction of being in India at a latitude of 20 degrees N some 75 million years ago is meaningless because at that time India was completely south of the equator.

Response R2.9: We removed the macroevolutionary analyses from the current ms.

L243: I do not understand at all what it means for a result to be "confounded with nocturnal ancestors".

Response R2.10: We removed the macroevolutionary analyses from the current ms.

Reviewer #3:

I was very excited to finally see a modern megaphylogeny of mammals (and one that was well done). This tree fills an important gap and will be a tremendous resource to the community of comparative biologists, macroecologists, and macroevolutionary researchers. It is also clear that this represents a tremendous amount of work. However, I am not as enthusiastic about the macroevolutionary analyses conducted here and think that this warrants some substantial revision before I would be comfortable recommending it for publication. (I also think it might be worth considering splitting the paper into two; obviously this is totally up to you but I do think that the two elements -- tree building and diversification analysis -- might both be worthy of a full paper on their own).

Response R3.1: We thank the reviewer for their helpful suggestion to split the paper in two. We have now done this, and it is allowing for considerably more explication of our phylogenetic analyses in the present (now revised) ms for PLOS Biology.

Major Comments

Given that the most substantial contribution of this paper (at least in my mind) is the phylogeny itself, it seemed very odd that so little attention was paid to the phylogenetic methodology and results in the main text. I.e., how well supported are traditionally recognized taxonomic groups in this phylogeny; how well does it correspond to previous large scale trees; did we learn anything new about the time scale of mammalian diversification that was not previously known? What remains unresolved by this effort? I think that some further attention to these points and particularly in the main text is very much deserved.

Response R3.2: Our revised Figs 2-6 now address these questions directly, as does our Discussion section. In particular, the “Backbone-level topology” subsection addresses some of the main topological controversies that remain in mammals. However, we note that our coverage of topological comparisons is not exhaustive – with a phylogeny of ~6000 species, it was clearly

not feasible to discuss all nodes of interest in a single manuscript. Furthermore, the primary goal of our study was to enable accurate estimation of tip-level rates of speciation – thus our focus was upon building a phylogeny where the branch lengths of all species were estimated comparably. Thus, our study did not aim to "resolve all the nodes" for mammals – particularly since the DNA sequences we incorporated have all already been published to the NCBI database. Rather, the novelty of our study is in placing existing sequences of our 31 sampled genes in a unified taxonomic and phylogenetic framework for the first time.

In general, I found it very difficult to pin down a clear question or clear answer in the diversification analyses. Even after reading through it several times, it was unclear how all the different analyses related to one another or more to the literature more broadly. You investigated lots of different things: rate shifts across clades, across time, clade age v. diversity, transitions across KPg (with a fossil analyses thrown in and incompletely described), associations with latitude, diurnality, and vagility. It was hard to ascertain what it all meant and what precisely we learned that we did not know before.

Response R3.3: We removed the macroevolutionary analyses from the present ms to focus on the construction and validation of our mammal phylogenies.

One area that I definitely think warrants more attention is the fact that rates of diversification seem to increase towards the present. What does this precisely mean and how does the results you present here, including the relationships between diversification rate, diversity, and time relate to similar recent studies on this topic (Rabosky et al. 2012 PLoS Bio, Rabosky 2015 Evolution, Machac et al. 2018 GEB, Marin and Hedges 2018 MBE, Henao Diaz et al 2019 PNAS). In their summation, do your findings confirm or contradict these previous results.

Response R3.4: N/A since our phylogeny-focused ms excludes the macroevolutionary analyses.

Was there any particularly reason you didn't statistically test for an uptake at the KPg boundary rather than just eyeballing the graph (line 122). There are a number of statistical methods (Stadler 2012 PNAS, May et al. 2016 Methods in Ecology and Evolution) and graphical inspection is not sufficient to my mind.

Response R3.5: N/A see above.

The analyses you conducted in which you broke apart the tree into different time bins was very difficult to understand and to interpret. Honestly I am having trouble commenting intelligently about since I am quite confused about what exactly you did and what exactly it means. I think the explanation of this requires considerable attention.

Response R3.6: N/A see above.

One of the traits that you suggest might promote speciation is vagility. First I don't think you make a tremendously strong case as to why this might be the case (I had no prior belief that this would be the case). Second and more problematically, if I am understanding you correctly was intrapolated from body size and home range data from a model fit to 89 species. This seemed

odd to me for the following reasons: i) how could you suggest from this that it was vagility per se and not body size or home range that is actually related to diversification?; and ii) the estimates of the prediction model differed substantially from that of Whitmee and Orme with very similar data -- if the model predictions are so sensitive to taxonomic breadth of the data (including a handful more species), how reliable can the predictions actually be when going from 89 to 5000+? At the very least some error in these estimates should be incorporated.

Response R3.7: N/A see above.

Minor Comments

I think it is quite silly to suggest that rate variation suggests that "ecology" plays a role in diversification (190); ecology here is so broadly defined as to be meaningless. And furthermore, of course ecology plays a role in diversification whether there is rate variation or not. And it also seems odd to contrast ecology and time as evolutionary forces…neither really appear to be forces per se

Response R3.8: N/A see above.

I don't think the finding that some species rich clades have rate variation within them is particularly interesting (line 151) because as you state, there is both more opportunity for rate shifts to occur in larger groups and more power to detect to those shifts. It is hard for me to interpret the biological meaning of this result.

Response R3.9: N/A see above.

I think that result that the latitudinal gradient does not mirror differences in speciation rate is less novel than you suggest. This has been pointed out several times for mammals in particular (e.g., Wier and Schluter 2006 Science among others) and appears to be a quite general trend according to a recent review of the topic (Schluter and Pennell 2017 Nature).

Response R3.10: N/A see above.

Line 214 - Both the evidence in support of this hypothesis and the reasoning behind it are unclear to me.

Response R3.11: N/A see above.

Line 130 - The observation that all nearly all the shifts were positive is very intriguing and I think deserves a bit more attention

Response R3.12: N/A see above.
