# Supplementary material for: Inferring the mammal tree: Species-level sets of phylogenies for questions in ecology, evolution, and conservation
Source: PLoS Biol. 2019 Dec 4;17(12):e3000494. doi: 10.1371/journal.pbio.3000494 (PMC6892540; doi:10.1371/journal.pbio.3000494)
Supplement: Attachment — Submitted filename: RESPONSE_to_minorRevisions_25Sep2019.pdf [file pbio.3000494.s029.pdf]

Response to reviewers for 'Inferring the mammal tree: species-level sets of phylogenies for questions in ecology, evolution, and conservation'

Reviewer #2:
[identifies himself as Olaf R.P. Bininda-Emonds]

With the revision, the MS has been sharply refocused on the topology of the mammalian timetree with the macroevolutionary analyses being reduced to discussions of diversification rates. As such, there is much more room for a discussion about how the timetree was generated and how it compares to other mammalian timetrees of similar scale, which I welcome greatly. Much of the information in this regard in the previous version was very limited because of space limitations and I do find this topic to be particularly important, especially with the authors contending previously that their tree was a large improvement over other efforts. The results here instead show much more agreement between these trees and much more uncertainty within the new timetree, which seems to be a accurate reflection of the state of affairs.

This is not to say that I do not find the new timetree to be impressive. I certainly do! I did not expect there to be a DNA-based phylogeny of mammals on such a scale for the next 10 years perhaps. so this study, at least from my perspective, is way ahead of its time!

Response R2.1: We thank Dr. Bininda-Emonds for his gracious comments, and appreciation of the revised focus of our manuscript. We agree that having the extra space to explicitly compare our tree to previous works—including the seminal contribution from his group in 2007—is beneficial to the broader research community for understanding the statistical properties of these different phylogenetic tree products. There is a general need for greater attention upon large-tree building methods and their disparate influences upon eco-evolutionary inferences, both in terms of conclusions and the level of confidence we place in them. Our revised ms now contributes to this debate while advancing mammalian species-level phylogenetics, to which we are grateful.

In general, I am very satisfied with the revisions and the additional information that the authors could provide about the timetree and have one major comment and several more minor ones.

The major comment

Although I think that this tree is a great advancement over previous supertrees, I also feel that the authors are being unfair in their criticism of supertrees. Contrary to what the authors repeatedly state, polytomies are not inherent to supertrees, but rather to the entire non-Bayesian framework in which the supertrees were conducted. Even supermatrices computed with MP and theoretically with ML will contain polytomies because a non-Bayesian framework ignores equally optimal trees in general to focus on the consensus solution. More importantly, it doesn't have to. If one were to look at all equally most optimal solutions or to use the set of bootstrap trees that were generated, one would get a rough equivalent to the credible sets of trees of a Bayesian analysis. "Ignoring" or "hiding" uncertainty is not inherent to supertrees or other non-Bayesian analyses, but rather a choice that is probably rooted partly historically when computer performance was insufficient to handle subsequent analyses on so many trees. That other

investigators misinterpret what a soft polytomy means (PG 29) is not the fault of the investigators who quite clearly stated that these were soft polytomies and knew what this meant.

Response R2.2: We thank the referee for this helpful critique, and now the opportunity to elucidate our treatment of supertrees in the context of their historical development. Here is our updated section on Page 29 (now on P16, starting at Line 447):

> Resolving those polytomies changes the tree shape, but does not reflect the considerable associated uncertainty in node ages and relationships. That is, the unresolved nodes produced in supertree studies when nodes conflict are ~~best considered~~ 'soft' polytomies, where the data needed to resolve a given node is lacking [1], ~~(~~as opposed to 'hard' polytomies where rapid divergence effectively leads to a star phylogeny [2]~~)~~. Collapsing uncertainty into ~~Rather than reflecting the large phylogenetic uncertainty inherent in~~ soft polytomies was a purposeful tool for~~,~~ supertree methods to yield a single consensus picture of evolutionary topology for more species than possible under joint inference [1,3,4]~~mask it~~.
>
> The danger, of course, has been when ~~here~~ soft polytomies ~~is that supertrees~~ are misinterpreted by subsequent investigators who assume that ~~the predominant~~all temporal and phylogenetic signatures in supertrees ~~is~~ are driven by biological processes ~~rather than methodological artifacts~~. For example, the study of Stadler et al. [5] made an important modeling advance for detecting tree-wide shifts in diversification rates, but the biological conclusion of a major rate shift ~30 Ma in rodents was apparently driven by soft polytomies in the MRP supertree (see Fig. 5b). Miscommunication between the stated purpose of supertrees—"to produce phylogenies based on all data sources" ([4]: 266)—and the need for big trees to additionally model all uncertainty in those data sources appears to have limited the durability of supertree-based inferences, and perhaps non-Bayesian methods generally [6].

See also our other responses to R2 comments below, which similarly soften our treatment of supertrees to be more accurate regarding the history of this field.

The minor comments

PG 5, L133: These comments are unfair and unnecessary. First, the taxonomy we used was the most modern one available and arguably every study (including this one) can be argued to be based on an outdated taxonomy because updates are continuously occurring. Second, the dating errors (which were generally very small; and 25 nodes in bats are also not that many of the total number) were due to a bug in one of the programs, were recognised, and corrected and only the data from the corrected version are now available. There is no need to discredit all the dates in the tree when similar and unknown bugs probably exist in all software used in such analyses, including the ones in this study.

Response R2.3: We see the referee's point that this paragraph could be read as negative—however, that was not our intention. Our goal was to provide the reader with a mini-review of the

different versions of the MRP supertree and the taxonomic and statistical properties that they contain. To help clarify our points, we have made several changes (new P6, starting on Line 161):

> The~~is~~ MRP~~consensus~~ supertree was based on the ~~an older~~ taxonomy of ~~(~~(*Mammal Species of the World*, 2nd edition (~~;~~ MSW2 [7]) and ~~included errors in the use of published dates (particularly within bats [18,73]). It~~ was ~~subsequently~~ updated twice: (i) Fritz et al. [8] linked the taxonomy to 5,020 of the 5,415 species in MSW3 [9], plus fixed errors in the dating of bats [10,11]; and (ii) Kuhn et al. [12] resolved the >50% of unresolved nodes (2,503 polytomies) remaining in the MRP supertree~~. Neither of those updates added new data. However, the latter one secondarily resolved each of the supertree's 2,503 unresolved nodes~~ using a stochastic birth-death model, creating a set of 1,000 trees with random variation in the placement of unresolved species [12]. Versions of the MRP supertree have been widely applied to questions of species diversification (e.g., [5,13–15]) and conservation (e.g., [11,16–18]) despite the ~~artifacts~~ initially~~of~~ unresolved ~~nodes~~species and consequent potential for artifacts in downstream analyses, in part because ~~they~~ it contained~~were~~ the only estimates of ~~species-level~~ branch lengths ~~for many~~across most of M~~m~~ammali~~a~~ia~~n clades~~.

PG 7, L171: I have no idea what "steps of merging, collapsing, and re-scaling" refers to and why this is so bad. Merging and re-scaling would also seem to be part and parcel of the backbone-and-patch method used here, which, albeit in a cruder fashion, was also employed to generate the topology of the mammal supertree. As for the mammal supertree at least, no re-scaling was performed either (also PG 29, L872; the only thing that was fixed was the topology, not the temporal backbone (whatever that is)). The entire topology was dated in one step in one piece.

Response R2.4: Thanks for pointing out our imprecise language, which is important in this end-Introductory paragraph. We clarified now this phrase to be (Line 205):

> Steps of merging overlapping sources, collapsing conflicting nodes, and applying point-estimate dates to scale phylogenies to time ~~re-scaling branches~~ are common to the MRP supertree, DNA supertree, and consensus timetree analyses.

We note that the backbone-and-patch approach, as explained in the Introduction and Methods, differs from supertrees in each of these points by: (i) estimating non-overlapping patch clades that correspond to branches in the backbone; (ii) having no overlapping nodes among the patch clades that could disagree and need to be collapsed; and (iii) using a distribution of node ages from the backbone to propagate age-estimate uncertainty from root to tip throughout the tree. The **non-overlapping** aspect of the backbone-and-patch approach is a key difference from supertree, but perhaps not clear before (only on Line 82)—to amend this, we now

- added italic emphasis on Line 77,
- added "non-overlapping" to Line 23, 81, 102, 432, 673, and 834, and

- on Line 834 wrote: “We divided the mammalian phylogeny into 28 patch clades that were non-overlapping in their ingroup species membership”.

PG 23, L679: As a personal observation without any real evidence to back it up, my experience has been that dating ancestral nodes based on descendant divergence times (which is what tip-dating essentially does) tends to overinflated the ages of the deepest nodes. In mentioning this to Tanja Stadler, she has also noticed the same phenomenon.

Response R2.5: We tend to agree with the referee here, but think that tip-dating has promise for continuing to develop the use of tree priors to correct some these dating biases. That is why we applaud the future potential for tip dating in this paragraph (now Line 266), but still focus our discussion on the node-dating analyses in the rest of the paper.

PG 28, L837: Retaining uncertainty has less to do with the backbone-and-patch framework than the Bayesian perspective in which it is performed. Backbone-and-patch (i.e., Mischler's compartmentalization) is arguably also the method that was used to construct the mammal supertree.

Response R2.6: We respectfully disagree with the referee here, since their statement appears to stem from the abovementioned misunderstanding that the backbone-and-patch approach uses overlapping sources similar to MRP supertrees. Rather, the use of non-overlapping patch clades circumvents the need to collapse any disagreeing nodes among sources, since none of the nodes can disagree if they do not overlap. Thus, the estimated uncertainty from source tree analyses can be directly propagated into the full tree assembly in the backbone-and-patch approach.

The innovation of two non-overlapping levels of analysis – which Mischler first conceived, but did not implement – is usefully paired with the Bayesian inference of each level. However, without the two-level approach, Bayesian joint analysis would be limited to ~800-1000 species (as we note) due to computational limits. To clarify this advantage of joining Bayesian inference with the non-overlapping backbone-and-patch analyses, we changed this passage to read (Line 1008-1010):

> We emphasize the retained uncertainty in the placental backbone divergence (Fig. 2c) as a strength of ~~our~~ the backbone-and-patch approach, since having two levels of non-overlapping Bayesian analysis enables temporal information to be passed ~~analysis~~ forward to the species tips~~framework~~.

PG 28, L853: Not "best considered", they *are* soft polytomies and nothing to the contrary was ever implied.

Response R2.7: We thank the referee for pointing out this discrepancy—we now change this sentence to the following (and cite the glossary in Bininda-Emond et al. 2004 – Line 448):

> That is, the unresolved nodes produced in supertree studies when nodes conflict are ~~best considered~~ ‘soft’ polytomies, where the data needed to resolve a given node is lacking [1], ~~(~~as opposed to ‘hard’ polytomies where rapid divergence effectively leads to a star phylogeny [2]).

PG 28, L856: Not supertree methods, but any tress produced in a non-Bayesian framework. Even ML trees mask topological uncertainty. The reason that so few / no polytomies are found there is because it's almost impossible to get equally likely topologies down to the xth decimal place.

Response R2.8: We removed the phrase about 'masking' phylogenetic uncertainty because we agree that it was misleading – instead we have changed this section to be a fairer interpretation of the supertree literature (Line 447 – see also R2.2 response above):

> Resolving those polytomies changes the tree shape, but does not reflect the considerable associated uncertainty in node ages and relationships. That is, the unresolved nodes produced in supertree studies when nodes conflict are ~~best considered~~ 'soft' polytomies, where the data needed to resolve a given node is lacking [1], ~~(~~as opposed to 'hard' polytomies where rapid divergence effectively leads to a star phylogeny [2]~~)~~. Collapsing uncertainty into ~~Rather than reflecting the large phylogenetic uncertainty inherent in~~ soft polytomies was a purposeful tool for~~,~~ supertree methods to yield a single consensus picture of evolutionary topology for more species than possible under joint inference [1,3,4]~~mask it~~.

PG 28, L869: I would actually argue that deeper dates are more reliable than ones closer to the tips because of the greater confidence in the calibration points and the greater margin of error allowed. It is far easier to confidently assert a fossil as being a mammal than belonging to a given genus, for instance. Genera as such are taxonomically more unstable (cf. Rattus) and, as an unsupported example, a mouse-like fossil will often end up in the genus Mus by default. (Or at least it used to. But how many of these old assertions have been retested?). It's definitely a mammal, but is it Mus or how exactly is it related to Mus? Tricky. Also, Given that mammals are somewhere around 166 million years old, a few million years of uncertainty in the fossil WRT its placement or age won't be a big deal. It definitely can have a major impact when trying to calibrate a node that itself is only a few million years old!

Response R2.9: The referee argues here that older mammal fossils are more likely to be confidently placed both taxonomically and temporally than are younger ones. We respectfully disagree based on the following points:

(i) Placing a fossil under "Mammalia" is unlikely to be useful for dating purposes, its rather the differential placement in crown vs. stem orders and families using cladistic characters that is relevant for dating our backbone-level phylogeny.

(ii) To that end, older fossils are known to be more susceptible to 'stem-ward slippage' or taxonomic loss (see papers by Sansom et al. [19,20]) and thus give more uncertain phylogenetic placement than younger fossils.

(iii) With a few exceptions for well-dated volcanic deposits (e.g., Gran Barranca in South America) where U-Pb dating or similar is possible, correlational dating further back in time is more likely to be associated with wider confidence limits given the greater likelihood of net erosion in those sediments (see [21]).

Given these points, we add a note of clarification to the sentence in question (Line 482):

Are paleomammalogists actually more certain about the timing of events near the K-Pg extinction event ~66 Ma then they are about modern divergences? Although this seems unlikely given preservation biases in the fossil record (e.g., [19,21]), that is the information conveyed by the DNA supertrees.

Olaf R.P. Bininda-Emonds

Reviewer #3:

The phylogeny (or rather set of phylogenies) that you have produced here is a big scientific achievement and I'm sure will be a major resource for the community of comparative biologists for years to come. This is a really substantial contribution to the field and I recognize how much effort this represents. Fantastic work! I have one major comment and a couple of very minor comments that I think might improve the paper.

Response R3.1: Awesome, thanks for the encouragement— The long road has ultimately improved this study quite a bit for the community of researchers to now use.

Major comment:

I understand that the main text of the present paper is essentially a reformatted/restructured version of the supplemental material of your previously submitted paper. But I think that broadly speaking, you could do much more to trim the main text more to work better as a paper -- one that can be read and digested by the broad group of people who are likely interested in using this phylogeny. While I appreciate your attention to detail, I think it is worth giving a long hard look at each section and paragraph and deciding whether it is indeed essential for the general reader of this paper or whether it fits better in a supplement. Even as a reviewer (and someone interested in the technical bits of the paper) it was a difficult paper to read as really important methodological points were mixed in with very minute details. Rather than being prescriptive (e.g., keep such and such paragraph in the main text), I am just leaving this as a general impression; please do what you think is best here.

Response R3.2: We thank R3 for this helpful comment. Although we mostly agree with the Associate Editor in wanting to keep the majority of methodological detail in the main text, we deleted some unnecessary details and moved a 3-paragraph section under 'Construction of full dated mammalian phylogenies' regarding exceptions to the rescale-and-graft to the *Supplementary Information* (see new section 7 there).

Minor comments:

Perhaps I missed it in the text (and I apologize if I did), but I would ask you to please make available the *dated* set of trees with and without PASTIS-placed species. Some researchers (myself included) who are interested in investigating the diversification dynamics of mammals might prefer to analyze this data without having the distribution of branch lengths assumed by a particular tree model. I know you are providing the ML estimate of the gene-only tree but I was wondering if you were supplying the full dated posterior estimates as well.

Response R3.3: Yes indeed! We are calling these the 'DNA-only' trees (4098 species total) in comparison to the 'completed' trees (5911 species) where missing species are taxonomically imputed using PASTIS. These DNA-only trees will be in credible sets of 10,000 trees as well, and in the same tip- and node-dated context as the completed trees. So there will be a total of four credible sets of Mammalia-wide trees distributed as part of our study.

First lines of abstract and introduction: I found the focus on speciation/extinction rates a bit misplaced. Honestly proper the largest use case for this set of phylogenies is going to be from comparative biologists who want to use a tree to do PGLS. I think it is worth broadening the opening to discuss some of the important questions in mammalian evolution (including of course diversification rates) that a robust phylogenetic hypothesis can be used to address.

Response R3.4: We thank the referee for pointing out this very valid point – we made the following changes to Abstract:

> Big, time-scaled phylogenies ~~Rates of speciation and extinction~~ are fundamental to connecting~~our understanding of~~ evolutionary processes to modern biodiversity patterns. Yet~~, but~~ inferring ~~these parameters requires~~ reliable phylogenetic trees for thousands of species involves trade-offs that have limited their utility to comparative biologists~~ies~~.

And this change to the first sentence in the opening paragraph of the Introduction:

> Reconstructing the timing and patterns of evolutionary relationship in the tree of life ~~a robust timescale for evolution is central to fathoming~~illuminates the processes of species birth (speciation),~~ and~~ death (extinction), character evolution, and many other fundamental aspects of biodiversity generation and maintenance ~~that have generated modern gradients of species diversity~~ [14,22].

Line 88: I think some credit for the development of this approach to building megaphylogenies should be given to Smith, Beaulieu and Donoghue 2009 BMC

Response R3.5: We agree that its helpful to put the Smith et al. 2009 method for building big trees in the context of the backbone-and-patch approach—we now add this sentence to that 4th paragraph of the Introduction:

> By comparison, the 'mega-phylogeny' approach of Smith et al. [23] used one level of maximum-likelihood analysis to construct large consensus trees that lack a distribution of estimated ages or relationships.

Line 328: The argument for not using ASTRAL III presented here isn't very satisfying. I am not suggesting you need to run this program but to say that you didn't use it because it was released in May 2018 (over a year before you submitted the current version of the paper) seems odd. If it really was a great solution (again, not saying it is) you had more than a year to run the analyses!

Response R3.6: We agree with the referee's comment and have decided to exclude mention of multi-species coalescent methods in the main text. Instead, we now have this section in the opening of the Supplementary Information:

> Our goal of including full uncertainty of divergence times and species relationships was best suited to Bayesian inference, where the likelihood of

> parameter estimates is reflected by their posterior frequency [6,24–26]. In contrast, maximum-likelihood and parsimony approaches to find the single 'best' point estimate effectively collapse tree uncertainty and can result in false confidence [6]. The ideal of using the multi-species coalescent to co-estimate genealogies and the species tree [27] in a Bayesian framework is unfortunately limited to ~25 species (e.g., *BEAST [28,29]). ML-based models (e.g., ASTRAL-II and ASTRAL-III [30,31]) have extended applications of the coalescent to larger trees, but analyzing the breadth of Mammalia in this context again only produces consensus trees, and requires subsequent steps of time calibration. Overall, we emphasize that the 'big tree' territory beyond ~1000 species requires new types of phylogenetic assumptions to overcome computational limits to co-estimation (Fig. S1).

Line 604: Minor technical comment (beyond the scope of this paper but perhaps worth mentioning): I think the interpretation of the DR statistic might be improved by formally deriving it in the context of the "identifiable" variables recently described by Louca and Pennell (BioRxiv)

Response R3.7: Thanks for pointing out this recent pre-print by Louca and Pennell, which we agree is an important contribution regarding information content in molecular phylogenies. Their main point is that diversification scenarios may not be distinguishable from each other if speciation and extinction rates are highly fluctuating through time. They develop their argument based on a tree's LTT trajectory and density, and thus their arguments are focused on deep-time inferences of diversification-rate variation through the internodes – rather than the tree tips – of a big phylogeny like ours. Our focus in building and characterizing the mammal tree has been upon these tip-rate estimates, which appear to be less biased by the type of rate fluctuations through time that Louca and Pennell mention.

To help clarify this point in our MS, we added the following passage to the Methods section on 'Tip-level speciation rates' (Line 1093):

> Tip DR emphasizes geologically recent speciation over deeper-time dynamics, and so is comparatively less prone to bias from undetected extinction events or non-identifiability [32] than methods for detecting branch-specific or tree-wide rate shifts [33–35].

Line 652: it is not really clear why using a single tree is likely to lead to overestimation errors (Type 1); what about Type II errors if the single tree is not the same as the posterior mode/median.

Response R3.8: This is a valid point by the referee that we are glad to fix—our real point is the false *confidence* implied by choosing one best phylogeny since the ensuing comparative methods will then treat it as the 'true' tree, i.e., known without error. We changed this sentence as follows (Line 231):

> Philosophically, our approach aimed to minimize the ~~overestimation~~ false confidence associated with ~~errors (i.e., false positives), which are inherent to~~ choosing one ‘best’ phylogeny to represent the complex, probabilistic landscape of reconstructed macroevolutionary history

And also changed this sentence in the Introduction (Line 109):

> This~~, which~~ is a feature especially designed to minimize inflated confidence ~~type I error (false positives)~~ in subsequent statistical tests where phylogenies are otherwise treated as known without error.

Line 1033: what is the basis for making the recommendation that 100 or 1000 trees will be sufficient to capture uncertainty. I generally think this is a reasonable suggestion but I don’t think you have provided evidence that this is actually a good rule of thumb.

Response R3.9: We agree that we did not demonstrate this rule of thumb, and so have removed this recommendation. Instead, we highlight the recent application of Rubin’s rules that demonstrate by simulation that 50-100 trees is a safe amount to sample from credible tree sets with ‘noisy’ branch lengths:

> Approaches that apply Rubin’s rules to address missing data in traits and phylogenetic sampling are particularly promising, suggesting that sampling 50-100 trees is sufficient to capture parameter uncertainty [36].

COMMENTS FROM THE ACADEMIC EDITOR:

I think the paper is an improvement and a good fit for the Methods & Resources section.

I agree that the remaining requests are largely textual in nature and should be dealt with through a Minor Revision. I'm not even sure I entirely agree with Rev #3's major comment about the level of detail being problematic given the article type. Sure the paper is dense, but I think people can skim through the sections that are most relevant to them - though maybe his point is more about the level of detail being inconsistent within sections. Irrespective, I do like the rigour and prescription with which steps are presented.

Response AE.1: We agree with you on this point, although on the advice of R3 still did skim the Methods section of our MS and made the following changes:

- Transferred three-paragraph section regarding exceptions to the rescale-and-graft procedure to the *S1 Text*, section 7 under ‘Construction of full dated mammalian phylogenies’
- Other small changes for clarity and brevity throughout.

I did, however, think that Rev #2's major comment needed addressing, especially the point that "ignoring" or "hiding" uncertainty is not inherent to supertrees but rather a reflection of the era in which many of these non-Bayesian methods were developed. This fix should be straightforward.

Response AE.2: We absolutely agree with this contention, and made several changes to alter the tone of our sections regarding supertrees and their role in the historical development of big tree methods from non-Bayesian into Bayesian conceptions. See above R2 responses.

The only other point I picked up on, and shared with Rev #3, was the argument for not using ASTRAL III on Line 328 wasn't satisfying (to say the least!).

Response AE.3: Thanks for allowing us to fix this – we removed our statement regarding ASTRAL from the main text, and now only mention multi-species coalescent methods in the *S1 Text* (opening section and S1 Fig).

REFERENCES

1. Bininda-Emonds ORP. The evolution of supertrees. Trends in Ecology & Evolution. 2004;19: 315–322. doi:10.1016/j.tree.2004.03.015

2. Lara MC, Patton JL, da Silva MNF. The Simultaneous Diversification of South American Echimyid Rodents (Hystricognathi) Based on Complete Cytochrome b Sequences. Molecular Phylogenetics and Evolution. 1996;5: 403–413. doi:10.1006/mpev.1996.0035

3. Sanderson MJ, Purvis A, Henze C. Phylogenetic supertrees: Assembling the trees of life. Trends in Ecology & Evolution. 1998;13: 105–109. doi:10.1016/S0169-5347(97)01242-1

4. Bininda-Emonds ORP, Gittleman JL, Steel MA. The (Super)Tree of Life: Procedures, Problems, and Prospects. Annual Review of Ecology and Systematics. 2002;33: 265–289. doi:10.1146/annurev.ecolsys.33.010802.150511

5. Stadler T. Mammalian phylogeny reveals recent diversification rate shifts. PNAS. 2011;108: 6187–6192. doi:10.1073/pnas.1016876108

6. Huelsenbeck JP, Rannala B, Masly JP. Accommodating Phylogenetic Uncertainty in Evolutionary Studies. Science. 2000;288: 2349–2350. doi:10.1126/science.288.5475.2349

7. Wilson DE, Reeder DM. Mammal Species of the World. A Taxonomic and Geographic Reference. Second Edition [Internet]. Second Edition. Washington: Smithsonian Institution Press; 1993. Available: https://www.biblio.com/book/mammal-species-world-taxonomic-geographic-reference/d/1084714387

8. Fritz SA, Bininda-Emonds ORP, Purvis A. Geographical variation in predictors of mammalian extinction risk: big is bad, but only in the tropics. Ecology Letters. 2009;12: 538–549. doi:10.1111/j.1461-0248.2009.01307.x

9. Wilson DE, Reeder DM. Mammal species of the world: a taxonomic and geographic reference, 3rd ed. 3rd ed. Baltimore, MD: Johns Hopkins University Press; 2005.

10. Bininda-Emonds ORP, Cardillo M, Jones KE, MacPhee RDE, Beck RMD, Grenyer R, et al. Corrigendum. Nature. 2008;456: 274. doi:10.1038/nature07347

11. Davies TJ, Fritz SA, Grenyer R, Orme CDL, Bielby J, Bininda-Emonds ORP, et al. Phylogenetic trees and the future of mammalian biodiversity. PNAS. 2008;105: 11556–11563. doi:10.1073/pnas.0801917105

12. Kuhn TS, Mooers AØ, Thomas GH. A simple polytomy resolver for dated phylogenies. Methods in Ecology and Evolution. 2011;2: 427–436. doi:10.1111/j.2041-210X.2011.00103.x

13. Cooper N, Purvis A. Body size evolution in mammals: complexity in tempo and mode. The American Naturalist. 2010;175: 727–738.

14. Purvis A, Fritz SA, Rodríguez J, Harvey PH, Grenyer R. The shape of mammalian phylogeny: patterns, processes and scales. Philosophical Transactions of the Royal Society of London B: Biological Sciences. 2011;366: 2462–2477. doi:10.1098/rstb.2011.0025

15. Belmaker J, Jetz W. Relative roles of ecological and energetic constraints, diversification rates and region history on global species richness gradients. Arita H, editor. Ecology Letters. 2015;18: 563–571. doi:10.1111/ele.12438

16. Isaac NJB, Turvey ST, Collen B, Waterman C, Baillie JEM. Mammals on the EDGE: Conservation Priorities Based on Threat and Phylogeny. PLOS ONE. 2007;2: e296. doi:10.1371/journal.pone.0000296

17. Collen B, Turvey Samuel T., Waterman Carly, Meredith Helen M. R., Kuhn Tyler S., Baillie Jonathan E. M., et al. Investing in evolutionary history: implementing a phylogenetic approach for mammal conservation. Philosophical Transactions of the Royal Society B: Biological Sciences. 2011;366: 2611–2622. doi:10.1098/rstb.2011.0109

18. Rosauer DF, Pollock LJ, Linke S, Jetz W. Phylogenetically informed spatial planning is required to conserve the mammalian tree of life. Proc R Soc B. 2017;284: 20170627. doi:10.1098/rspb.2017.0627

19. Sansom RS. Bias and Sensitivity in the Placement of Fossil Taxa Resulting from Interpretations of Missing Data. Syst Biol. 2015;64: 256–266. doi:10.1093/sysbio/syu093

20. Sansom RS, Gabbott SE, Purnell MA. Non-random decay of chordate characters causes bias in fossil interpretation. Nature. 2010;463: 797–800. doi:10.1038/nature08745

21. Holland SM. The non-uniformity of fossil preservation. Philosophical Transactions of the Royal Society B: Biological Sciences. 2016;371: 20150130. doi:10.1098/rstb.2015.0130

22. Schluter D, Pennell MW. Speciation gradients and the distribution of biodiversity. Nature. 2017;546: 48–55. doi:10.1038/nature22897

23. Smith SA, Beaulieu JM, Donoghue MJ. Mega-phylogeny approach for comparative biology: an alternative to supertree and supermatrix approaches. BMC Evolutionary Biology. 2009;9: 37. doi:10.1186/1471-2148-9-37

24. Huelsenbeck JP, B. Larget, R. E. Miller, F. Ronquist. Potential applications and pitfalls of Bayesian inference of phylogeny. Syst Biol. 2002;51: 673–688.

25. Douady CJ, Delsuc F, Boucher Y, Doolittle WF, Douzery EJP. Comparison of Bayesian and maximum likelihood bootstrap measures of phylogenetic reliability. Mol Biol Evol. 2003;20: 248–254.

26. Yang Z, Rannala B. Bayesian phylogenetic inference using DNA sequences: a Markov Chain Monte Carlo Method. Mol Biol Evol. 1997;14: 717–724. doi:10.1093/oxfordjournals.molbev.a025811

27. Degnan JH, Rosenberg NA. Gene tree discordance, phylogenetic inference and the multispecies coalescent. Trends in Ecology & Evolution. 2009;24: 332–340. doi:10.1016/j.tree.2009.01.009

28. Heled J, Drummond AJ. Bayesian Inference of Species Trees from Multilocus Data. Mol Biol Evol. 2010;27: 570–580. doi:10.1093/molbev/msp274

29. Zimmermann T, Mirarab S, Warnow T. BBCA: Improving the scalability of *BEAST using random binning. BMC Genomics. 2014;15: S11. doi:10.1186/1471-2164-15-S6-S11

30. Mirarab S, Warnow T. ASTRAL-II: coalescent-based species tree estimation with many hundreds of taxa and thousands of genes. Bioinformatics. 2015;31: i44–i52. doi:10.1093/bioinformatics/btv234

31. Zhang C, Rabiee M, Sayyari E, Mirarab S. ASTRAL-III: polynomial time species tree reconstruction from partially resolved gene trees. BMC Bioinformatics. 2018;19: 153. doi:10.1186/s12859-018-2129-y

32. Louca S, Pennell MW. Phylogenies of extant species are consistent with an infinite array of diversification histories. bioRxiv. 2019; 719435. doi:10.1101/719435

33. Quental TB, Marshall CR. Diversity dynamics: molecular phylogenies need the fossil record. Trends in Ecology and Evolution. 2010;25: 434–441.

34. Rabosky DL. Extinction rate should not be estimated from molecular phylogenies. Evolution. 2010;64: 1816–1824.

35. Beaulieu JM, Brian C. O'Meara. Extinction can be estimated from moderately sized molecular phylogenies. Evolution. 2015;69: 1036–1043.

36. Nakagawa S, De Villemereuil P. A General Method for Simultaneously Accounting for Phylogenetic and Species Sampling Uncertainty via Rubin's Rules in Comparative Analysis. Syst Biol. 2019;68: 632–641. doi:10.1093/sysbio/syy089
