# Supplementary material for: Inferring the mammal tree: Species-level sets of phylogenies for questions in ecology, evolution, and conservation
Source: PLoS Biol. 2019 Dec 4;17(12):e3000494. doi: 10.1371/journal.pbio.3000494 (PMC6892540; doi:10.1371/journal.pbio.3000494)
Supplement: S2 Data — Includes PDF plots of each gene tree along with the newick tree file and the DNA alignments in phylip format. Dryad data: https://doi.org/10.5061/dryad.tb03d03. RAxML, Randomized Axelerated Maximum Likelihood. (ZIP) [file pbio.3000494.s022.zip › S2_Data.zip!/Data_S2_geneTree_files/RAxML_bipartitions_fin3_BRCA2_plot.pdf]

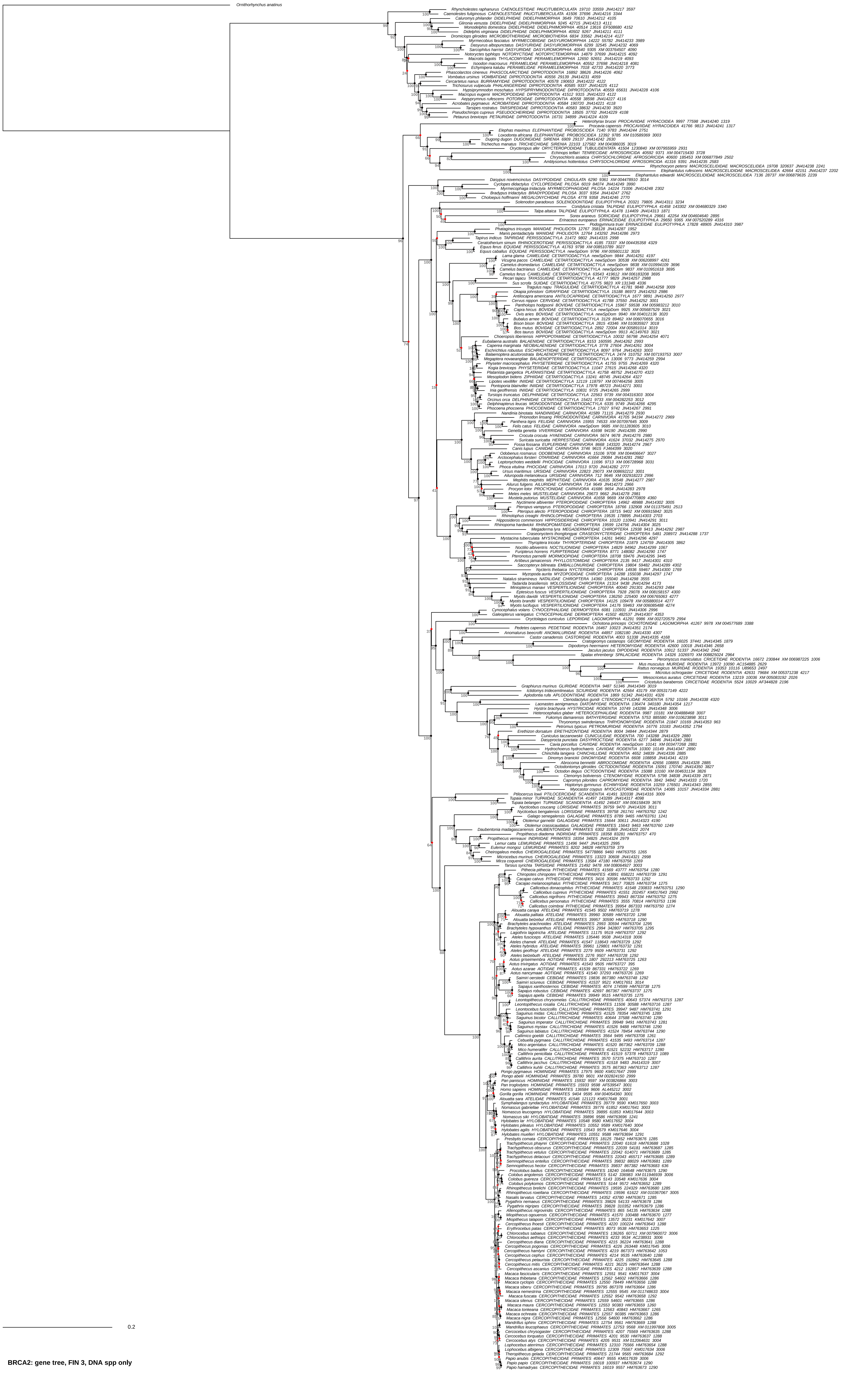

BRCA2: gene tree, FIN 3, DNA spp only
