# Supplementary material for: Inferring the mammal tree: Species-level sets of phylogenies for questions in ecology, evolution, and conservation
Source: PLoS Biol. 2019 Dec 4;17(12):e3000494. doi: 10.1371/journal.pbio.3000494 (PMC6892540; doi:10.1371/journal.pbio.3000494)
Supplement: S2 Data — Includes PDF plots of each gene tree along with the newick tree file and the DNA alignments in phylip format. Dryad data: https://doi.org/10.5061/dryad.tb03d03. RAxML, Randomized Axelerated Maximum Likelihood. (ZIP) [file pbio.3000494.s022.zip › S2_Data.zip!/Data_S2_geneTree_files/RAxML_bipartitions_fin3_CYTB_plot.pdf]

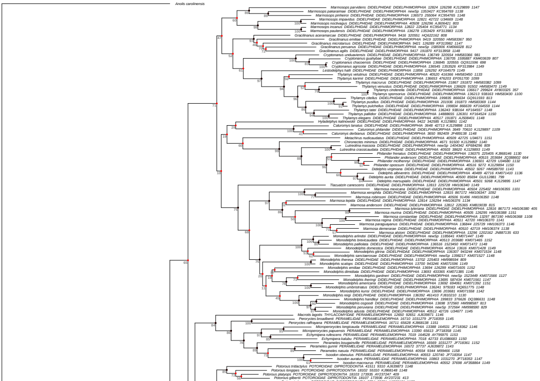
Anolis carolinensis
Marmosops parvidens DIDELPHIDAE DIDELPHIMORPHIA 12824 126298 KJ129899 1147
Marmosops pakaraimae DIDELPHIDAE DIDELPHIMORPHIA newSp 1353427 KC954769 1138
Marmosops pinheiroi DIDELPHIDAE DIDELPHIMORPHIA 136573 255064 KC954765 1148
Marmosops impavidus DIDELPHIDAE DIDELPHIMORPHIA 12821 42722 U34669 1148
Marmosops noctivagus DIDELPHIDAE DIDELPHIMORPHIA 40508 126296 AJ606421 803
Marmosops incanus DIDELPHIDAE DIDELPHIMORPHIA 12822 225404 KC954771 1134
Marmosops paulensis DIDELPHIDAE DIDELPHIMORPHIA 136278 1353429 KF313983 1135
Gracilinanus aceramarcae DIDELPHIDAE DIDELPHIMORPHIA 9416 320551 HQ622162 808
Gracilinanus emiliae DIDELPHIDAE DIDELPHIMORPHIA 9419 320550 HM583367 950
Gracilinanus microtarsus DIDELPHIDAE DIDELPHIMORPHIA 9421 126289 KF313982 1147
Gracilinanus peruanus DIDELPHIDAE DIDELPHIMORPHIA newSp 1585906 KM066028 812
Gracilinanus agilis DIDELPHIDAE DIDELPHIMORPHIA 9417 191870 KF313958 1148
Cryptonanus unduaviensis DIDELPHIDAE DIDELPHIMORPHIA 136749 320554 HM583366 981
Cryptonanus guahybae DIDELPHIDAE DIDELPHIMORPHIA 136705 1595887 KM403639 807
Cryptonanus chacoensis DIDELPHIDAE DIDELPHIMORPHIA 136845 320555 GQ911596 698
Cryptonanus agricolai DIDELPHIDAE DIDELPHIMORPHIA 136545 1353526 KF313984 1149
Lestodelphys halli DIDELPHIDAE DIDELPHIMORPHIA 11856 126292 KF164579 1149
Thylamys velutinus DIDELPHIDAE DIDELPHIMORPHIA 40520 416366 HM583450 1133
Thylamys karimii DIDELPHIDAE DIDELPHIMORPHIA 136653 476203 EF051700 1099
Thylamys macrurus DIDELPHIDAE DIDELPHIMORPHIA 21867 191872 HM583382 1099
Thylamys venustus DIDELPHIDAE DIDELPHIMORPHIA 136626 91502 HM583470 1149
Thylamys cinderella DIDELPHIDAE DIDELPHIMORPHIA 136617 299624 AY803325 357
Thylamys sponsorius DIDELPHIDAE DIDELPHIMORPHIA 136213 938163 HM583430 1100
Thylamys citellus DIDELPHIDAE DIDELPHIMORPHIA 199835 866634 GQ911593 813
Thylamys pusillus DIDELPHIDAE DIDELPHIMORPHIA 201936 191873 HM583369 1144
Thylamys pulchellus DIDELPHIDAE DIDELPHIMORPHIA 199834 866639 KF164559 1144
Thylamys tatei DIDELPHIDAE DIDELPHIMORPHIA 136243 938164 KF164557 1148
Thylamys pallidior DIDELPHIDAE DIDELPHIMORPHIA 14888655 126301 KF164524 1150
Thylamys elegans DIDELPHIDAE DIDELPHIMORPHIA 40517 191871 AJ508401 1148
Hyladelphys kalinowskii DIDELPHIDAE DIDELPHIMORPHIA 9422 342585 KJ129891 1142
Caluromys lanatus DIDELPHIDAE DIDELPHIMORPHIA 3648 42713 KJ129898 1151
Caluromys philander DIDELPHIDAE DIDELPHIMORPHIA 3649 70610 KJ129897 1109
Caluromys derbianus DIDELPHIDAE DIDELPHIMORPHIA 3650 992409 JF489138 1148
Metachirus nudicaudatus DIDELPHIDAE DIDELPHIMORPHIA 40509 42725 U34671 1151
Chironectes minimus DIDELPHIDAE DIDELPHIMORPHIA 4671 91500 KJ129892 1140
Lutreolina massoia DIDELPHIDAE DIDELPHIMORPHIA newSp 1404342 KF684296 809
Lutreolina crassicaudata DIDELPHIDAE DIDELPHIMORPHIA 40503 38620 KJ129893 1149
Philander frenatus DIDELPHIDAE DIDELPHIMORPHIA 136375 225405 KJ868146 1130
Philander andersoni DIDELPHIDAE DIDELPHIMORPHIA 40515 203684 JQ388602 664
Philander mcilhennyi DIDELPHIDAE DIDELPHIMORPHIA 136501 42729 U34680 1132
Philander opossum DIDELPHIDAE DIDELPHIMORPHIA 40516 9272 KJ129894 1150
Didelphis virginiana DIDELPHIDAE DIDELPHIMORPHIA 40502 9267 HM589700 1143
Didelphis albiventris DIDELPHIDAE DIDELPHIMORPHIA 40489 42716 KM071410 1136
Didelphis aurita DIDELPHIDAE DIDELPHIMORPHIA 40500 85694 GU112881 799
Didelphis marsupialis DIDELPHIDAE DIDELPHIMORPHIA 40501 9268 KJ129895 1147
Tlacuatzin canescens DIDELPHIDAE DIDELPHIMORPHIA 12813 225728 HM106340 1149
Marmosa mexicana DIDELPHIDAE DIDELPHIMORPHIA 40504 225402 HM106355 1101
Marmosa xerophila DIDELPHIDAE DIDELPHIMORPHIA 12815 867172 HM106347 1092
Marmosa robinsoni DIDELPHIDAE DIDELPHIMORPHIA 40506 91496 HM106350 1148
Marmosa lepida DIDELPHIDAE DIDELPHIMORPHIA 12814 126294 HM106376 1134
Marmosa andersoni DIDELPHIDAE DIDELPHIMORPHIA 12812 225365 KM819039 815
Marmosa tyleriana DIDELPHIDAE DIDELPHIMORPHIA 12816 867173 HM106380 405
Marmosa murina DIDELPHIDAE DIDELPHIMORPHIA 40505 126295 HM106388 1151
Marmosa constantiae DIDELPHIDAE DIDELPHIMORPHIA 13297 867190 HM106368 1108
Marmosa regina DIDELPHIDAE DIDELPHIMORPHIA 40511 42720 HM106370 1141
Marmosa paraguayanus DIDELPHIDAE DIDELPHIMORPHIA 136844 225729 HM106373 1146
Marmosa demerarae DIDELPHIDAE DIDELPHIMORPHIA 40510 42719 HM106374 1138
Marmosa alstoni DIDELPHIDAE DIDELPHIMORPHIA 13296 1202162 JN887135 633
Monodelphis arlindoi DIDELPHIDAE DIDELPHIMORPHIA newSp 1185641 KM071447 1149
Monodelphis brevicaudata DIDELPHIDAE DIDELPHIMORPHIA 40513 203680 KM071465 1152
Monodelphis palliolata DIDELPHIDAE DIDELPHIMORPHIA 136516 1523450 KM071472 1148
Monodelphis domestica DIDELPHIDAE DIDELPHIMORPHIA 40514 13616 KM071428 1149
Monodelphis glirina DIDELPHIDAE DIDELPHIMORPHIA 136307 943244 KM071534 1148
Monodelphis sanctaerosae DIDELPHIDAE DIDELPHIMORPHIA newSp 1398217 KM071527 1148
Monodelphis theresa DIDELPHIDAE DIDELPHIMORPHIA 13702 225403 HM998594 809
Monodelphis scalops DIDELPHIDAE DIDELPHIMORPHIA 13700 943246 KM071596 1149
Monodelphis emiliae DIDELPHIDAE DIDELPHIMORPHIA 13694 126299 KM071605 1152
Monodelphis dimidiata DIDELPHIDAE DIDELPHIMORPHIA 13693 433365 KM071385 1145
Monodelphis gardneri DIDELPHIDAE DIDELPHIMORPHIA newSp 1523449 KM071566 1127
Monodelphis iheringi DIDELPHIDAE DIDELPHIMORPHIA 13695 587434 KM071561 1147
Monodelphis americana DIDELPHIDAE DIDELPHIMORPHIA 13692 694061 KM071392 1151
Monodelphis umbristriatus DIDELPHIDAE DIDELPHIMORPHIA 136241 979183 HQ651775 1148
Monodelphis kunsi DIDELPHIDAE DIDELPHIMORPHIA 13696 203681 KM071558 1142
Monodelphis reigi DIDELPHIDAE DIDELPHIMORPHIA 136392 461410 FJ810210 1130
Monodelphis handleyi DIDELPHIDAE DIDELPHIMORPHIA 199833 376626 DQ386631 1148
Monodelphis osgoodi DIDELPHIDAE DIDELPHIMORPHIA 13698 372560 HM998587 813
Monodelphis peruviana DIDELPHIDAE DIDELPHIMORPHIA newSp 372564 HM998590 829
Monodelphis adusta DIDELPHIDAE DIDELPHIMORPHIA 40512 42726 U34677 1145
Macrotis lagotis THYLACOMYIDAE PERAMELEMORPHIA 12650 92651 AJ639871 1146
Peroryctes broadbenti PERAMELIDAE PERAMELEMORPHIA 16710 1031279 JF718359 1145
Peroryctes raffrayana PERAMELIDAE PERAMELEMORPHIA 16711 65628 KJ868138 1151
Microperoryctes longicauda PERAMELIDAE PERAMELEMORPHIA 13388 164531 JF718362 1146
Microperoryctes papuensis PERAMELIDAE PERAMELEMORPHIA 13390 65613 JF718358 1145
Echymipera rufescens PERAMELIDAE PERAMELEMORPHIA 7019 164528 AY795975 1153
Echymipera kalubu PERAMELIDAE PERAMELEMORPHIA 7018 42733 EU086683 1150
Perameles bougainville PERAMELIDAE PERAMELEMORPHIA 16569 1031277 JF718361 1152
Perameles gunnii PERAMELIDAE PERAMELEMORPHIA 16572 37737 AJ639872 1143
Perameles nasuta PERAMELIDAE PERAMELEMORPHIA 40554 9344 M99466 1158
Isoodon obesulus PERAMELIDAE PERAMELEMORPHIA 40553 120740 JF718354 1147
Isoodon auratus PERAMELIDAE PERAMELEMORPHIA 10863 1031270 JF718353 1147
Isoodon macrourus PERAMELIDAE PERAMELEMORPHIA 40552 37698 AF358864 1149
Potorous tridactylus POTOROIDAE DIPROTODONTIA 41511 9310 AJ639873 1148
Potorous longipes POTOROIDAE DIPROTODONTIA 18102 55310 KJ868148 1148
Potorous platyops POTOROIDAE DIPROTODONTIA 18103 173935 AY237247 409
Potorous gilbertii POTOROIDAE DIPROTODONTIA 18107 173936 AY237231 413

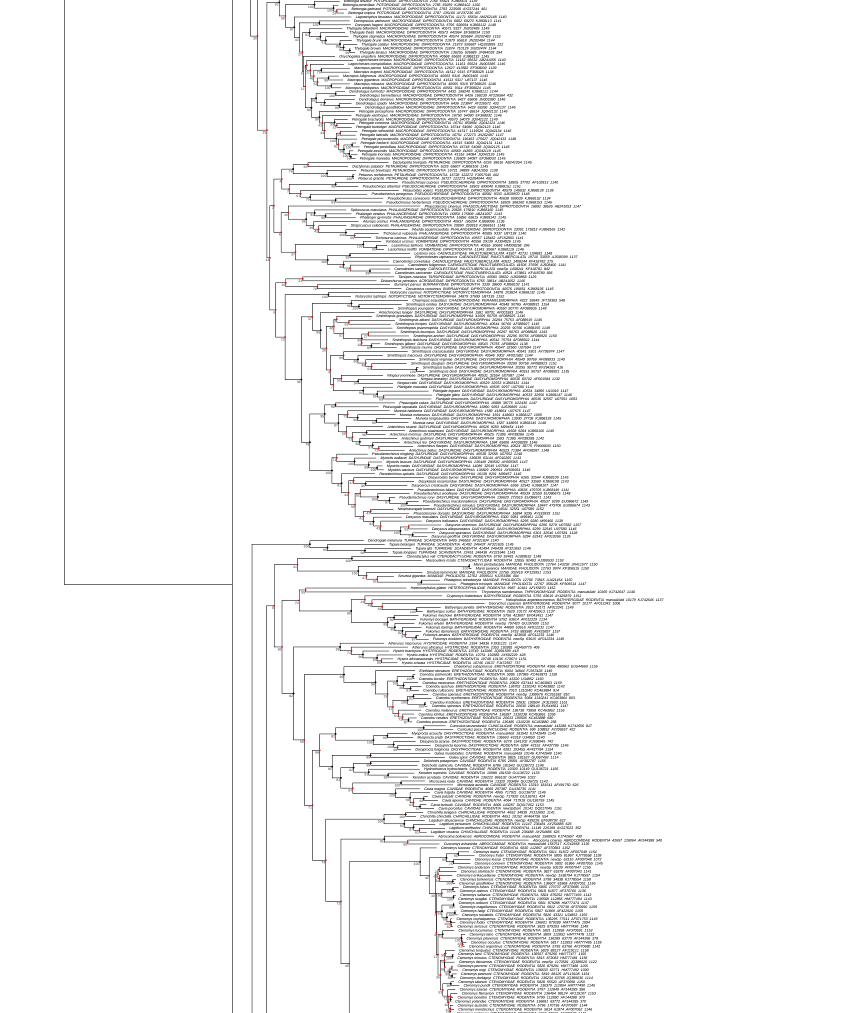

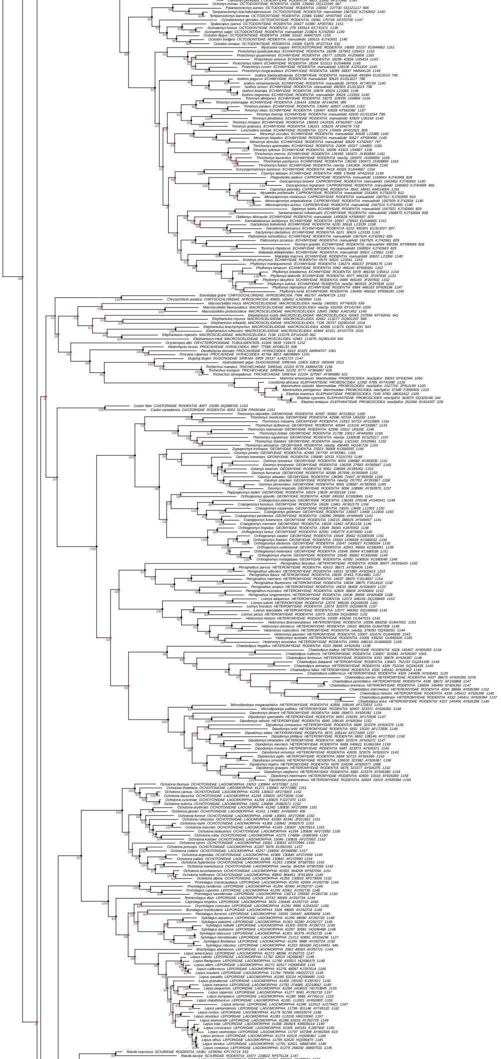

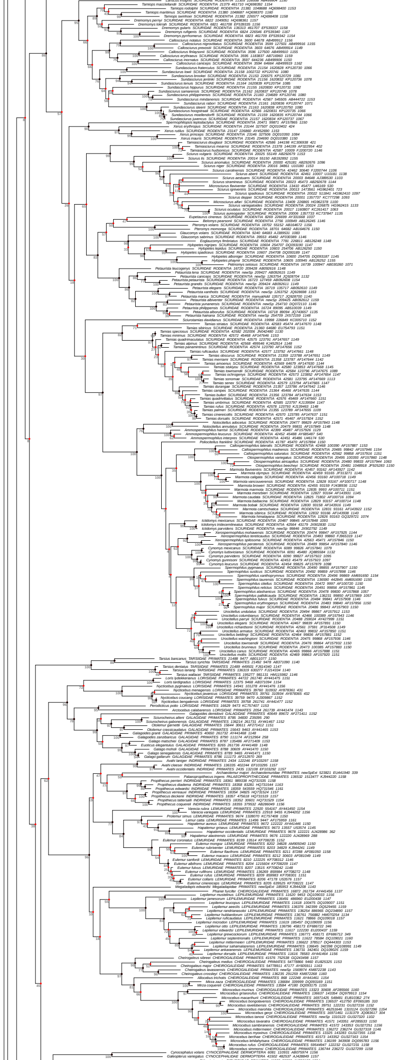

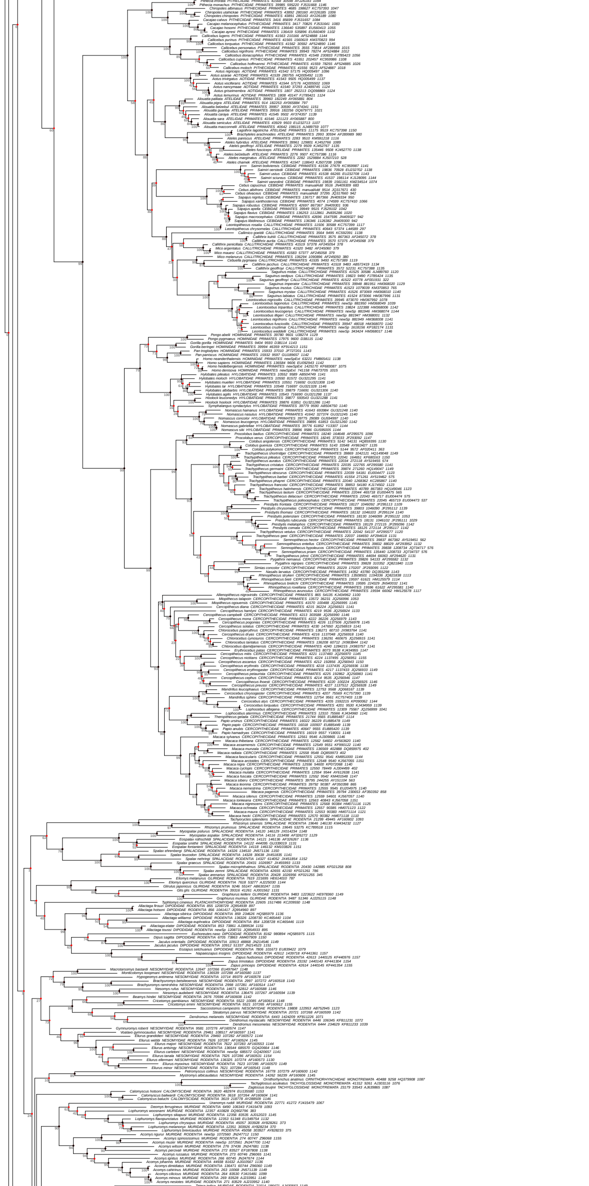

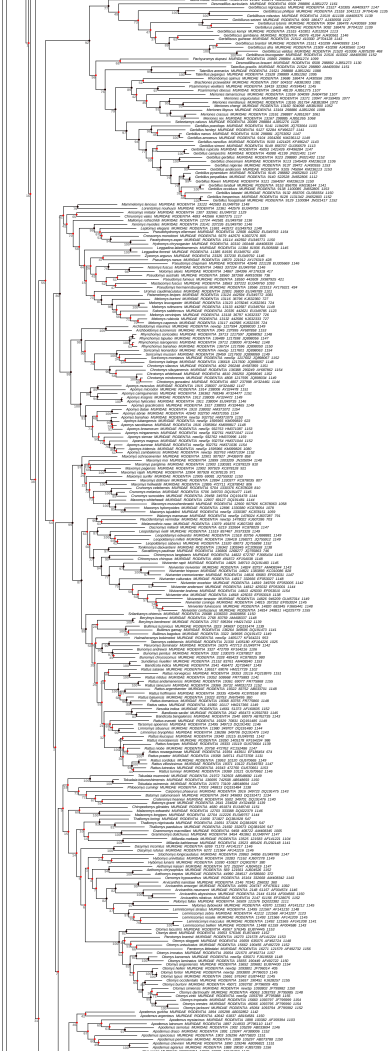

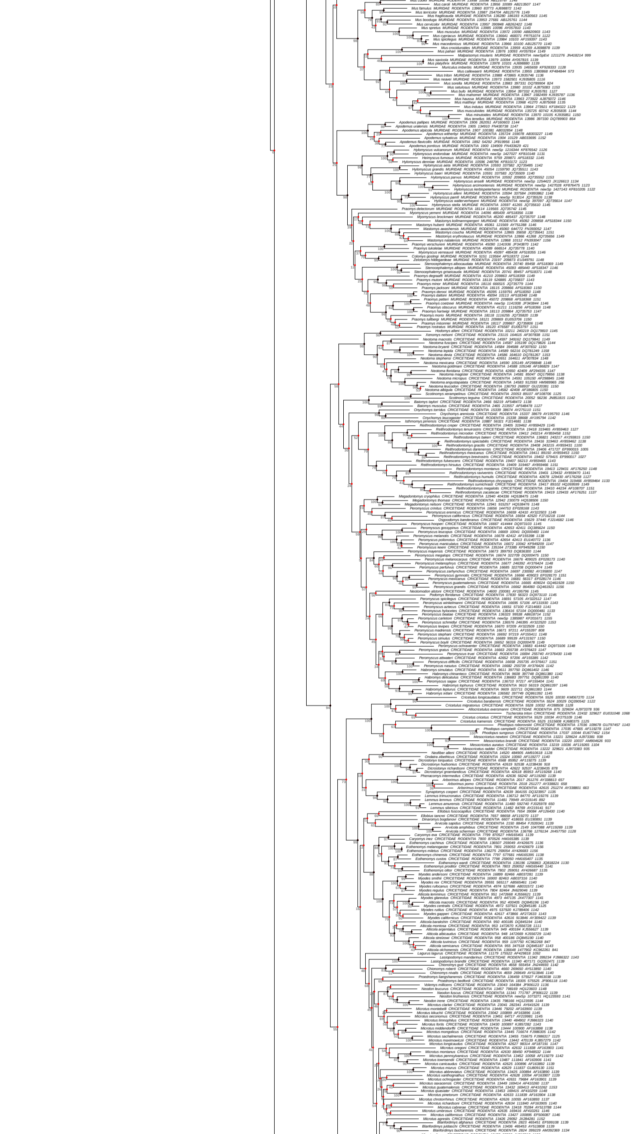

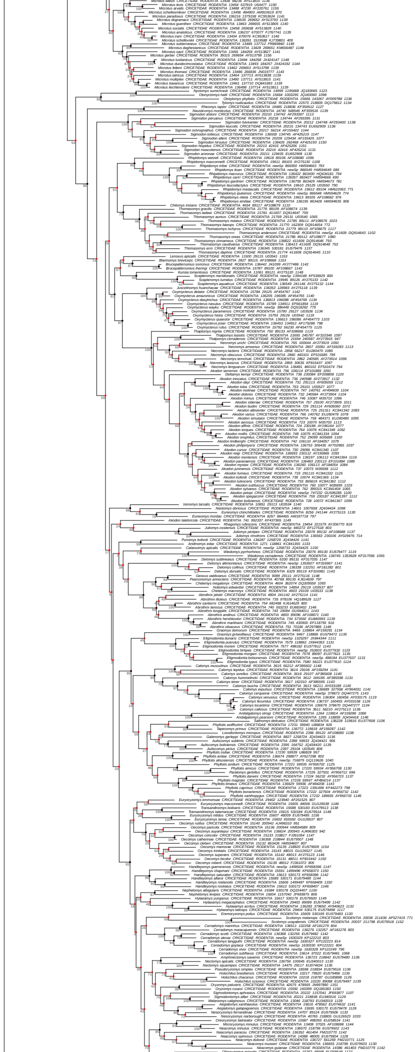

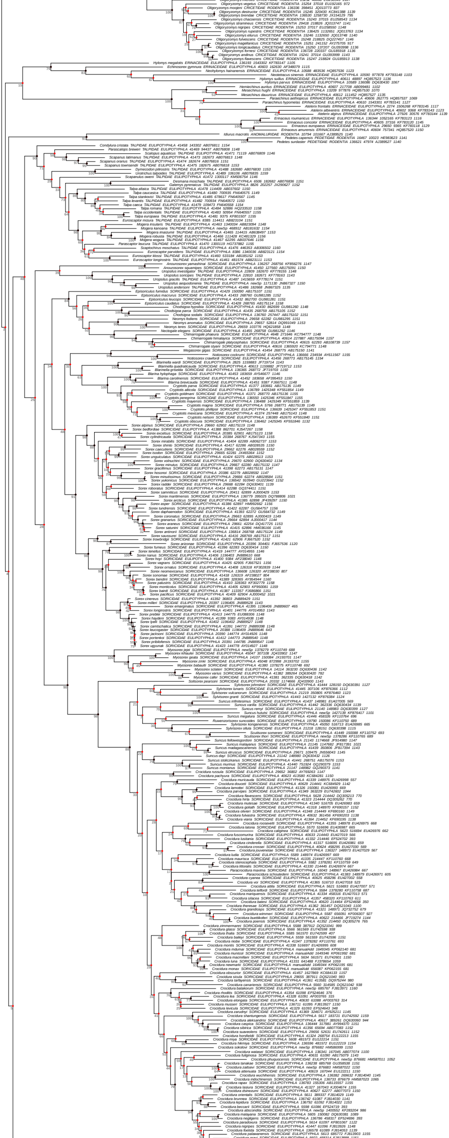

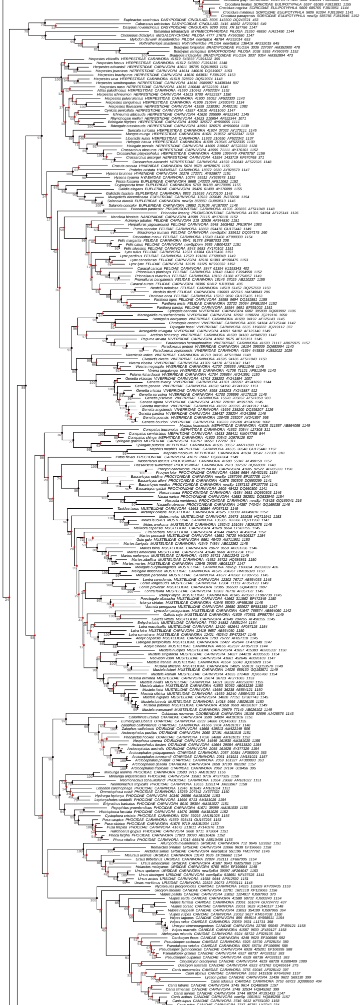

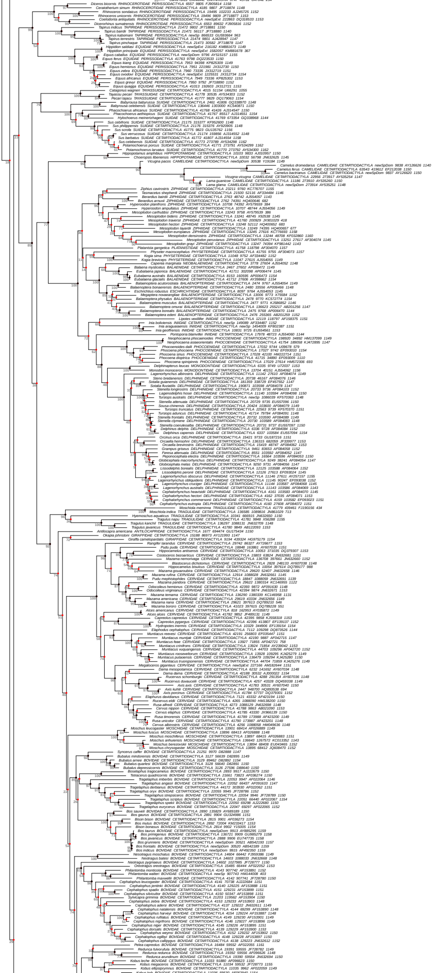

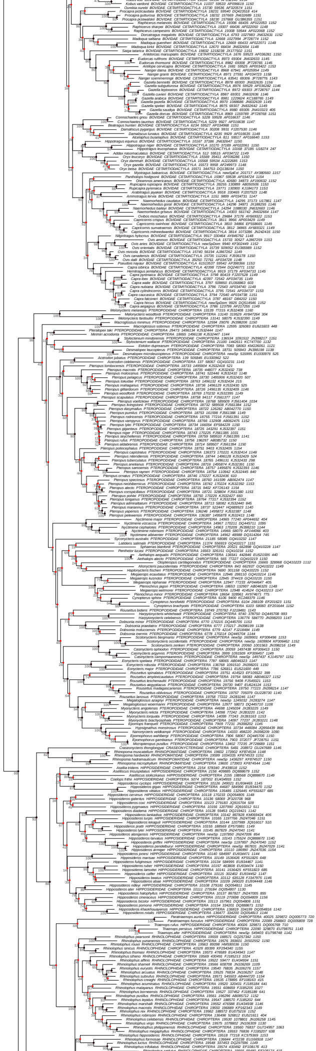

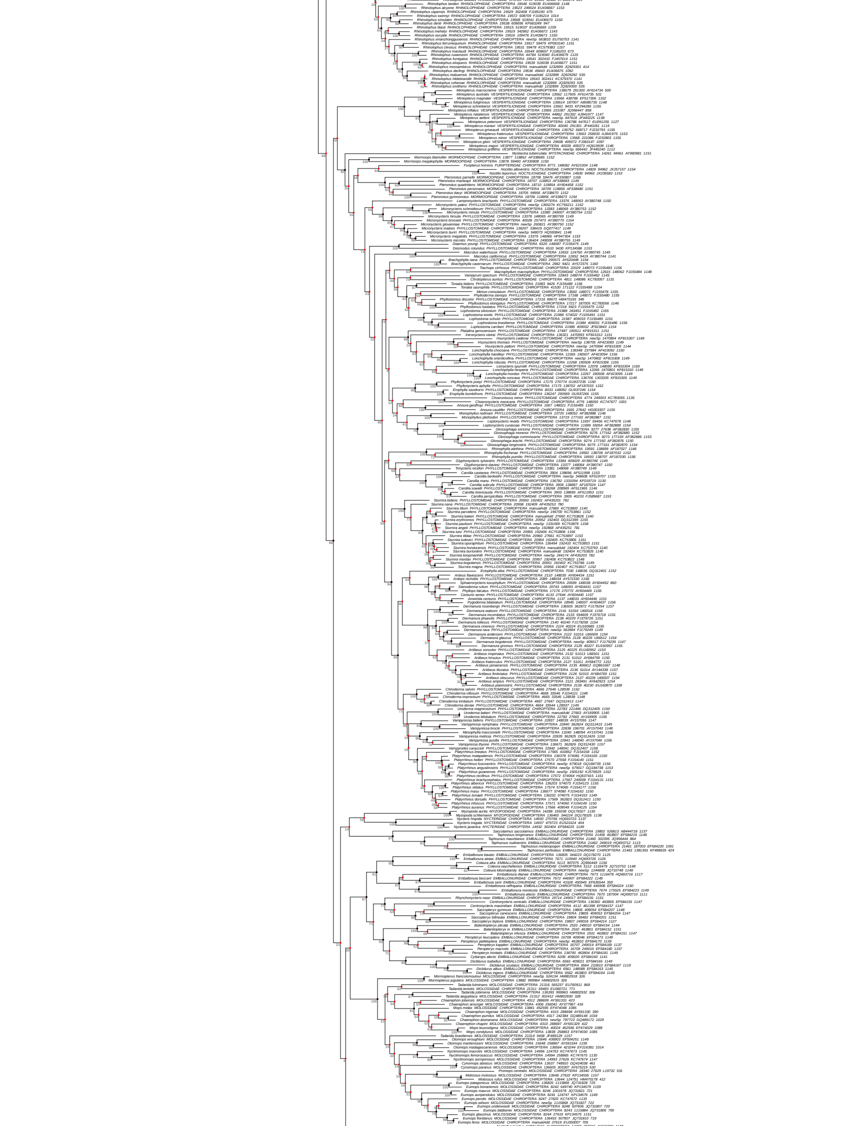

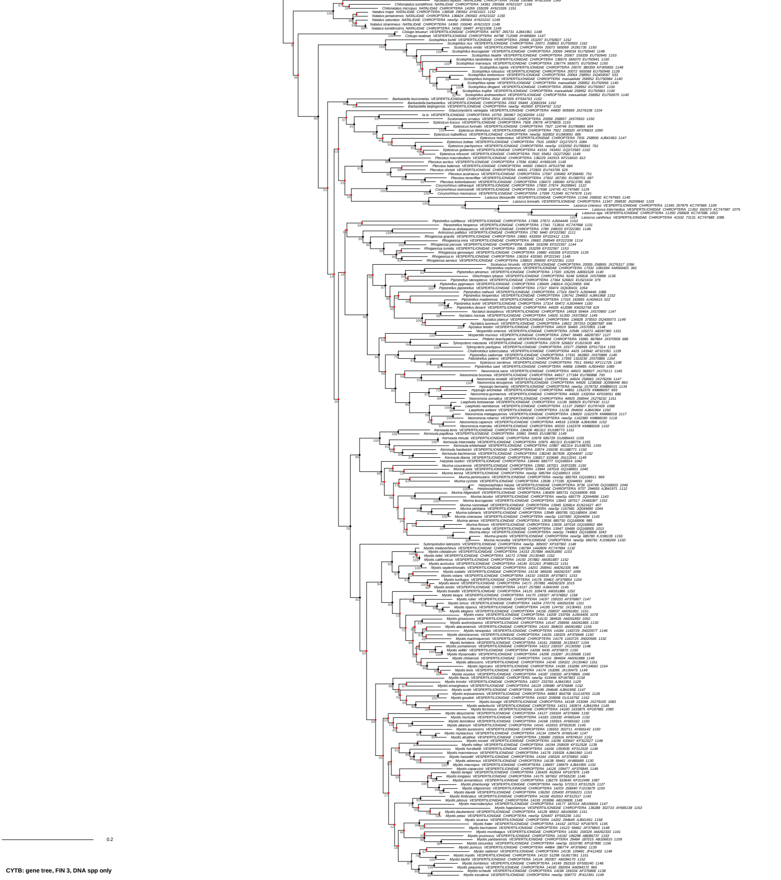


**CYTB: gene tree, FIN 3, DNA spp only**
