# Supplementary material for: Inferring the mammal tree: Species-level sets of phylogenies for questions in ecology, evolution, and conservation
Source: PLoS Biol. 2019 Dec 4;17(12):e3000494. doi: 10.1371/journal.pbio.3000494 (PMC6892540; doi:10.1371/journal.pbio.3000494)

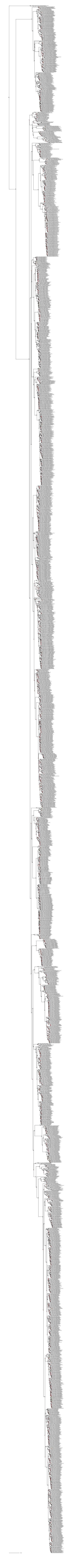

Supplement: S3 Data — Includes the full 31-gene supermatrix alignment, taxonomy file, newick tree file and PDF plotting of global RAxML tree, and R code for dividing the tree into patch clade segments for scaffolding the subsequent Bayesian analyses. Dryad data: https://doi.org/10.5061/dryad.tb03d03. ML, maximum-likelihood; RAxML, Randomized Axelerated Maximum Likelihood. (ZIP) [file pbio.3000494.s023.zip › S3_Data.zip!/Data_S3_globalRAxML_files/MamPhy_globalRAxML_FIN4_108bootstraps_4098mam_linear_agesBP.pdf]
