# Supplementary material for: Inferring the mammal tree: Species-level sets of phylogenies for questions in ecology, evolution, and conservation
Source: PLoS Biol. 2019 Dec 4;17(12):e3000494. doi: 10.1371/journal.pbio.3000494 (PMC6892540; doi:10.1371/journal.pbio.3000494)
Supplement: S4 Data — Relative time results (i.e., not yet time scaled) presented as MCC trees for each of the patch clade runs, both as nexus and PDF plots, and details of the species and gene sampling in each patch clade. Dryad data: https://doi.org/10.5061/dryad.tb03d03. MCC, maximum clade credibility. (ZIP) [file pbio.3000494.s024.zip › S4_Data.zip!/Data_S5_patchClade_results_and_MCC/PatchClades_MCC_Completed-TopoCons_mean/postburn10k_BDvr_FIXED_MCC_PC9_Spalacidae_plot.pdf]

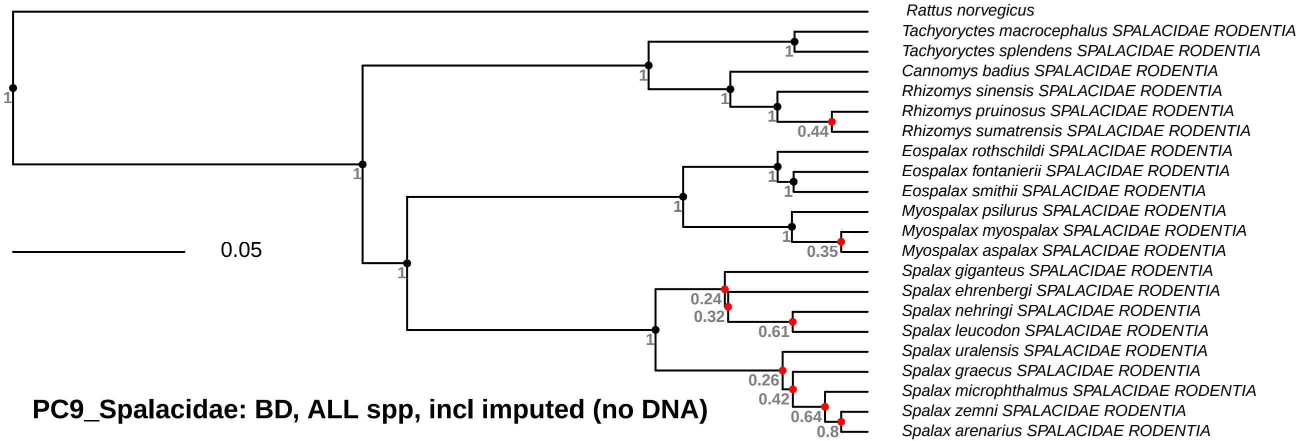
Rattus norvegicus
Tachyoryctes macrocephalus SPALACIDAE RODENTIA
Tachyoryctes splendens SPALACIDAE RODENTIA
Cannomys badius SPALACIDAE RODENTIA
Rhizomys sinensis SPALACIDAE RODENTIA
Rhizomys pruinosus SPALACIDAE RODENTIA
Rhizomys sumatrensis SPALACIDAE RODENTIA
Eospalax rothschildi SPALACIDAE RODENTIA
Eospalax fontanierii SPALACIDAE RODENTIA
Eospalax smithii SPALACIDAE RODENTIA
Myospalax psilurus SPALACIDAE RODENTIA
Myospalax myospalax SPALACIDAE RODENTIA
Myospalax aspalax SPALACIDAE RODENTIA
Spalax giganteus SPALACIDAE RODENTIA
Spalax ehrenbergi SPALACIDAE RODENTIA
Spalax nehringi SPALACIDAE RODENTIA
Spalax leucodon SPALACIDAE RODENTIA
Spalax uralensis SPALACIDAE RODENTIA
Spalax graecus SPALACIDAE RODENTIA
Spalax microphthalmus SPALACIDAE RODENTIA
Spalax zemni SPALACIDAE RODENTIA
Spalax arenarius SPALACIDAE RODENTIA
0.05
1
1
1
1
1
1
1
1
1
1
1
1
1
0.44
0.35
0.24
0.32
0.61
0.26
0.42
0.64
0.8
PC9_Spalacidae: BD, ALL spp, incl imputed (no DNA)
