# Supplementary material for: Inferring the mammal tree: Species-level sets of phylogenies for questions in ecology, evolution, and conservation
Source: PLoS Biol. 2019 Dec 4;17(12):e3000494. doi: 10.1371/journal.pbio.3000494 (PMC6892540; doi:10.1371/journal.pbio.3000494)
Supplement: S4 Data — Relative time results (i.e., not yet time scaled) presented as MCC trees for each of the patch clade runs, both as nexus and PDF plots, and details of the species and gene sampling in each patch clade. Dryad data: https://doi.org/10.5061/dryad.tb03d03. MCC, maximum clade credibility. (ZIP) [file pbio.3000494.s024.zip › S4_Data.zip!/Data_S5_patchClade_results_and_MCC/PatchClades_MCC_Completed-TopoCons_mean/_ALL28plots_postburn10k_BDvr_FIXED_MCCs.pdf]

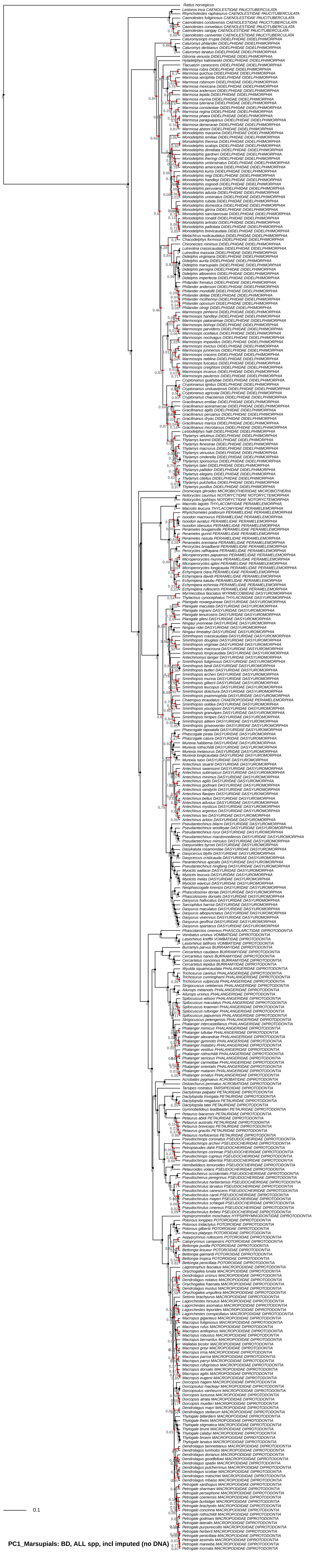

PC1_Marsupials: BD, ALL spp, incl imputed (no DNA)

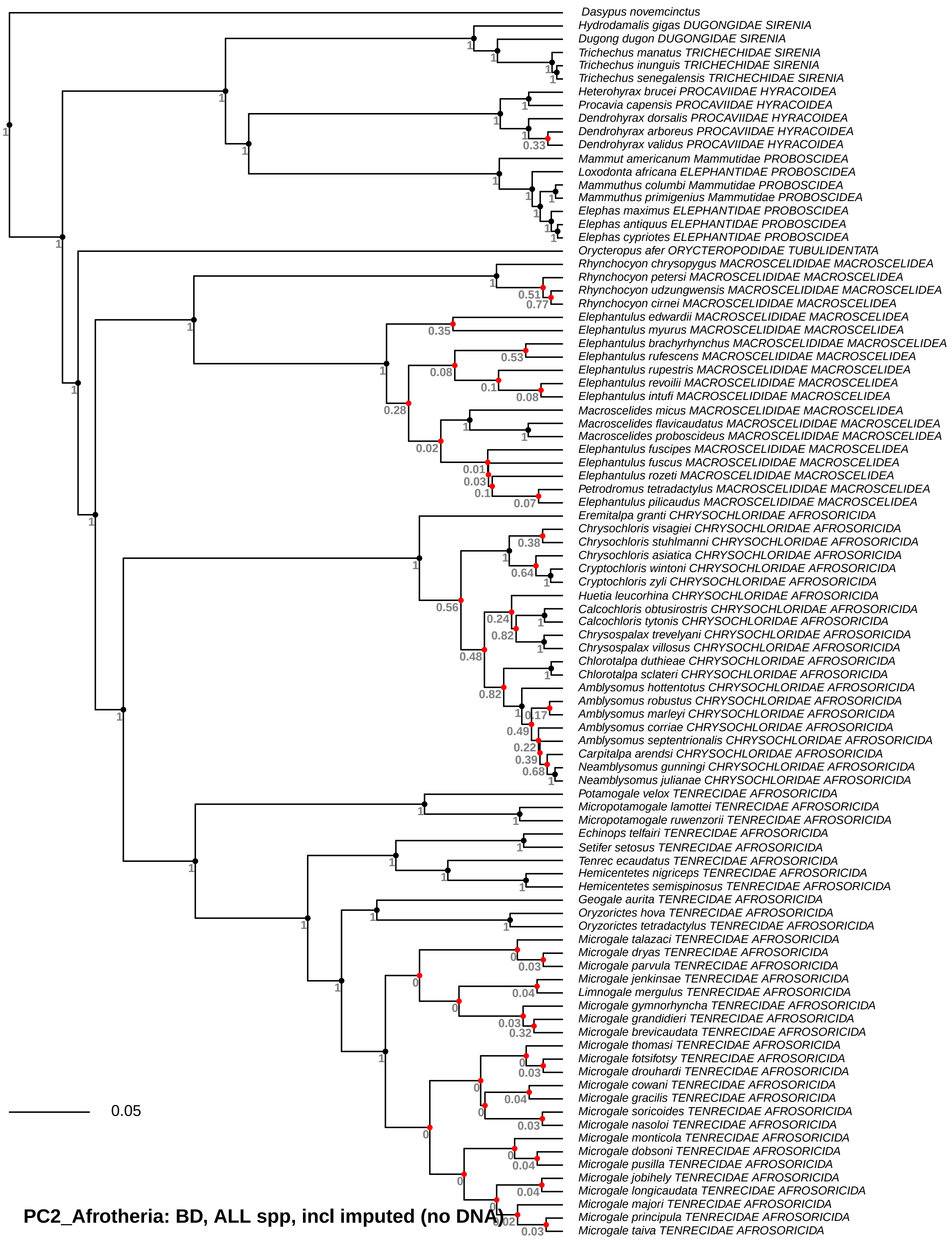

PC2_Afrotheria: BD, ALL spp, incl imputed (no DNA)

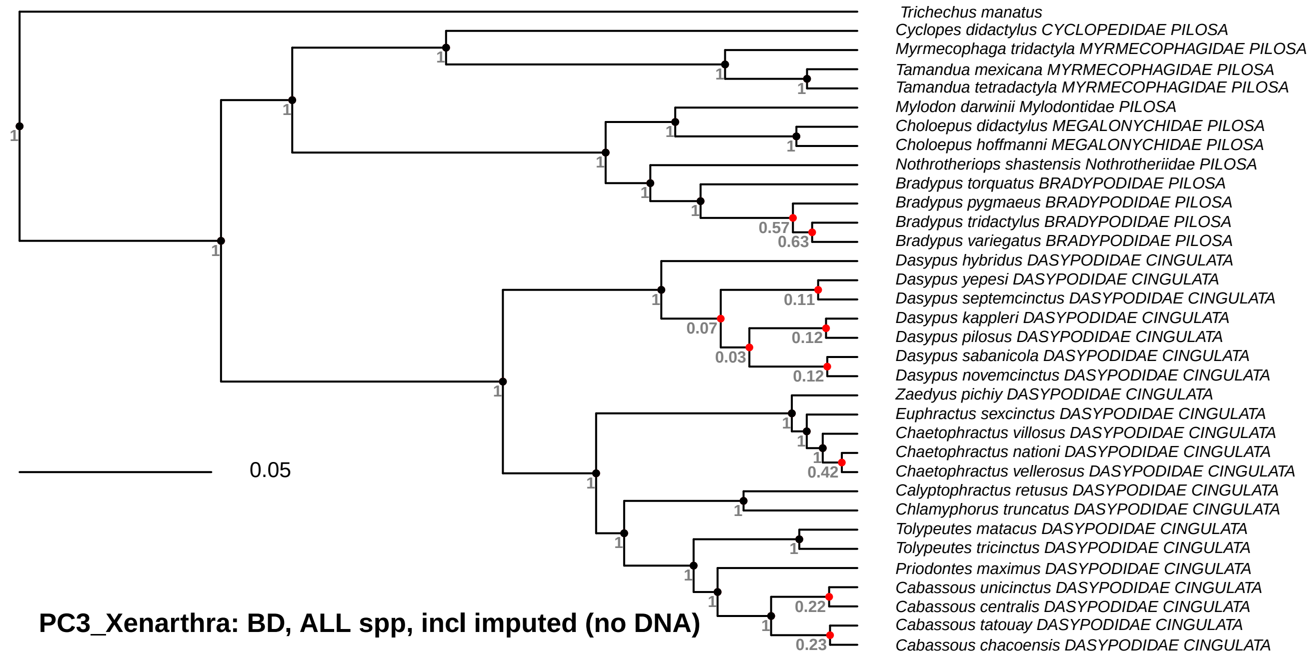
Trichechus manatus
Cyclopes didactylus CYCLOPEDIDAE PILOSA
Myrmecophaga tridactyla MYRMECOPHAGIDAE PILOSA
Tamandua mexicana MYRMECOPHAGIDAE PILOSA
Tamandua tetradactyla MYRMECOPHAGIDAE PILOSA
Mylodon darwinii Mylodontidae PILOSA
Choloepus didactylus MEGALONYCHIDAE PILOSA
Choloepus hoffmanni MEGALONYCHIDAE PILOSA
Nothrotheriops shastensis Nothrotheriidae PILOSA
Bradypus torquatus BRADYPODIDAE PILOSA
Bradypus pygmaeus BRADYPODIDAE PILOSA
Bradypus tridactylus BRADYPODIDAE PILOSA
Bradypus variegatus BRADYPODIDAE PILOSA
Dasypus hybridus DASYPODIDAE CINGULATA
Dasypus yepesi DASYPODIDAE CINGULATA
Dasypus septemcinctus DASYPODIDAE CINGULATA
Dasypus kappleri DASYPODIDAE CINGULATA
Dasypus pilosus DASYPODIDAE CINGULATA
Dasypus sabanicola DASYPODIDAE CINGULATA
Dasypus novemcinctus DASYPODIDAE CINGULATA
Zaedyus pichiy DASYPODIDAE CINGULATA
Euphractus sexcinctus DASYPODIDAE CINGULATA
Chaetophractus villosus DASYPODIDAE CINGULATA
Chaetophractus nationi DASYPODIDAE CINGULATA
Chaetophractus vellerosus DASYPODIDAE CINGULATA
Calyptophractus retusus DASYPODIDAE CINGULATA
Chlamyphorus truncatus DASYPODIDAE CINGULATA
Tolypeutes matacus DASYPODIDAE CINGULATA
Tolypeutes tricinctus DASYPODIDAE CINGULATA
Priodontes maximus DASYPODIDAE CINGULATA
Cabassous unicinctus DASYPODIDAE CINGULATA
Cabassous centralis DASYPODIDAE CINGULATA
Cabassous tatouay DASYPODIDAE CINGULATA
Cabassous chacoensis DASYPODIDAE CINGULATA
0.57
0.63
0.11
0.07
0.12
0.03
0.12
0.42
0.22
0.23
0.05
PC3_Xenarthra: BD, ALL spp, incl imputed (no DNA)

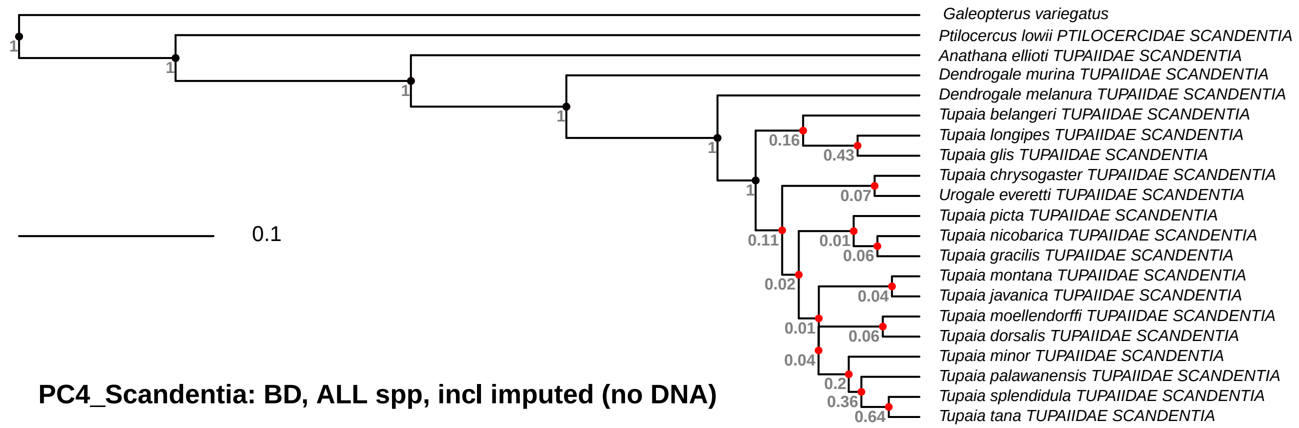

PC4_Scandentia: BD, ALL spp, incl imputed (no DNA)

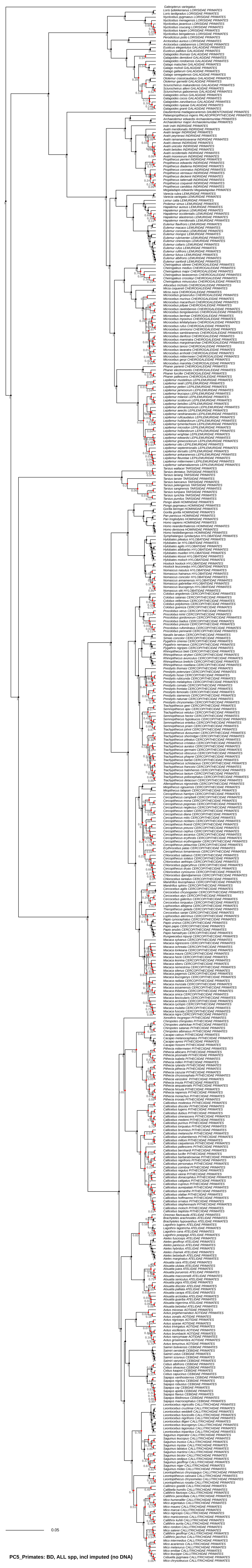

PC5_Primates: BD, ALL spp, incl imputed (no DNA)

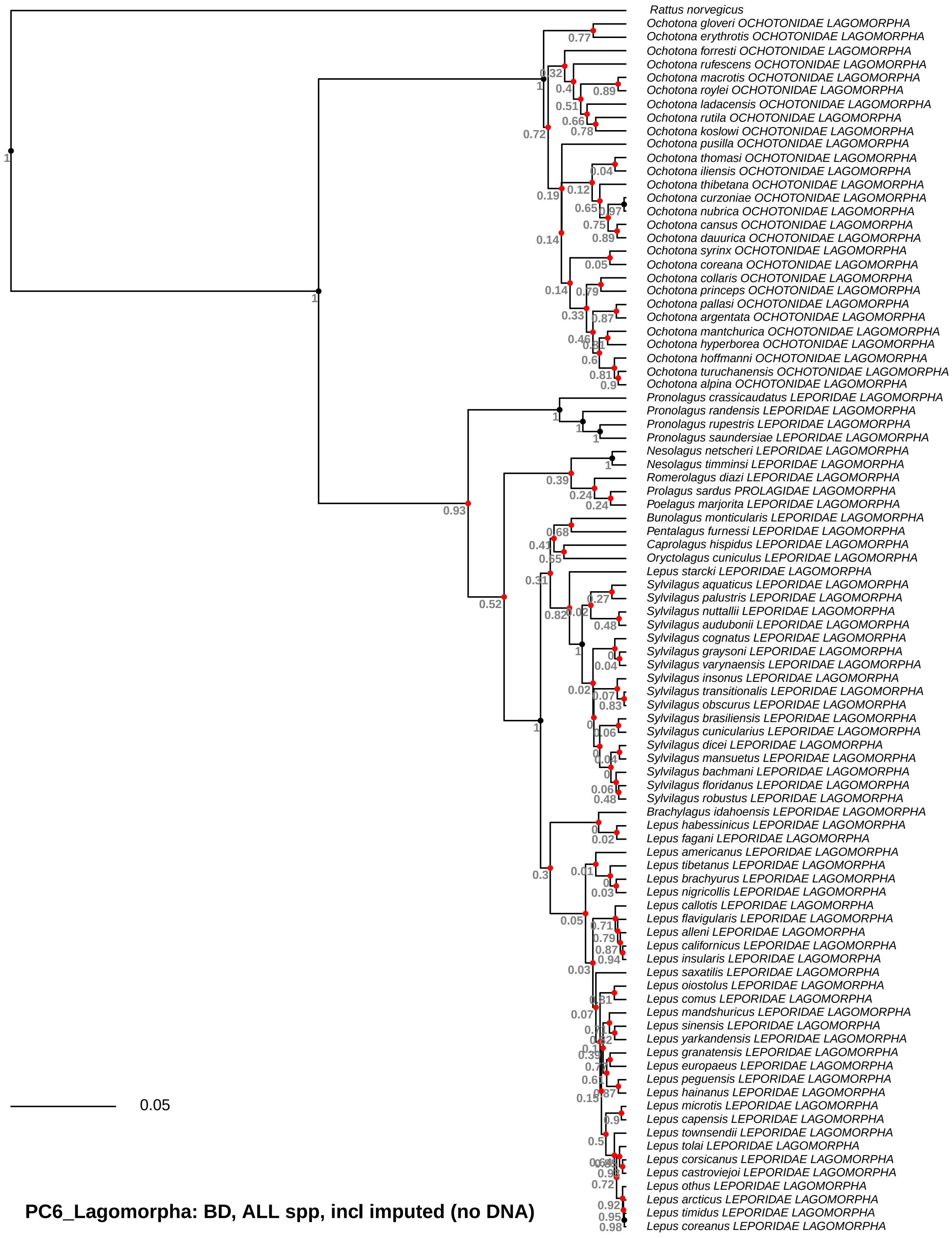

PC6_Lagomorpha: BD, ALL spp, incl imputed (no DNA)

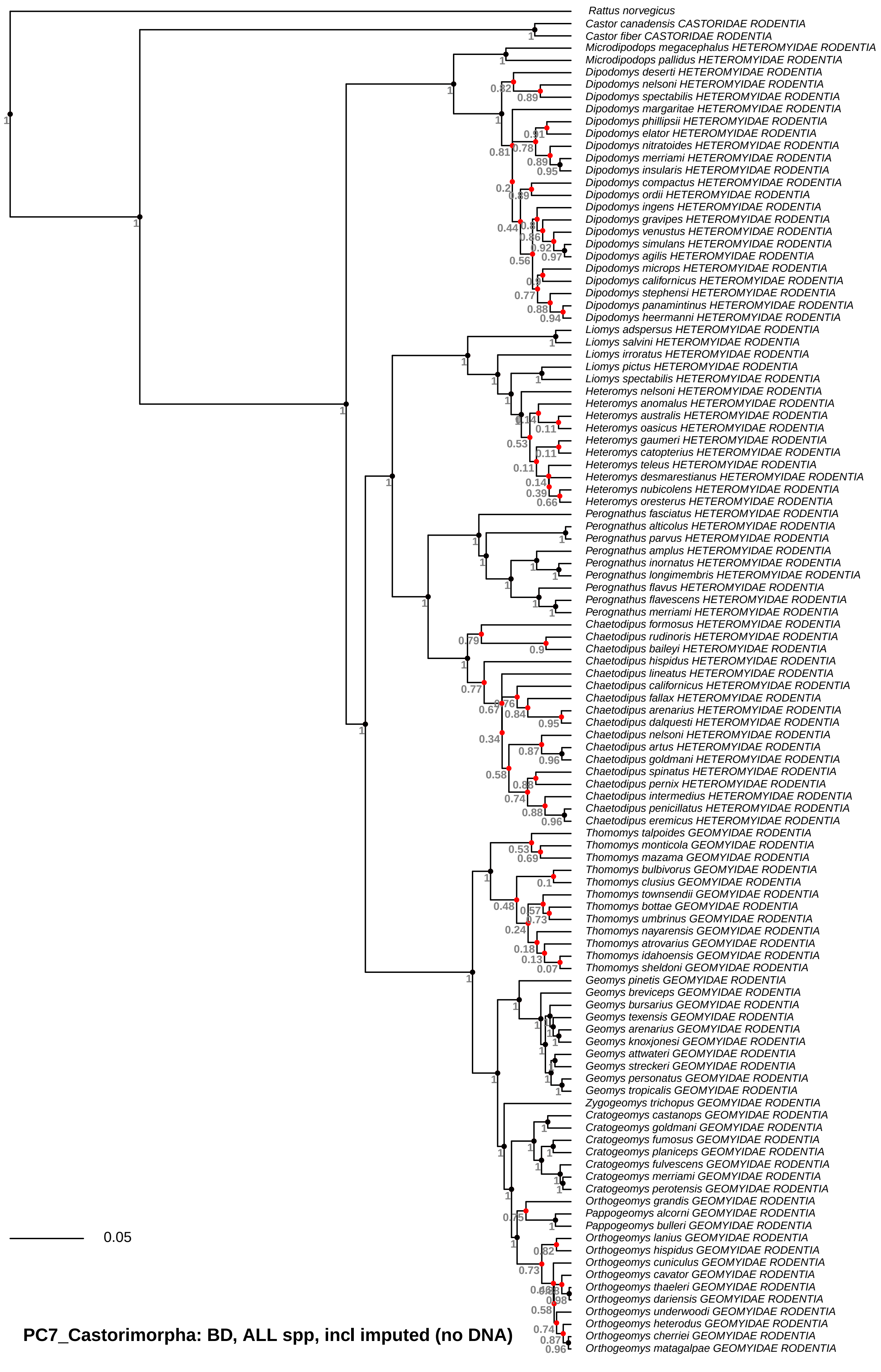

PC7_Castorimorpha: BD, ALL spp, incl imputed (no DNA)

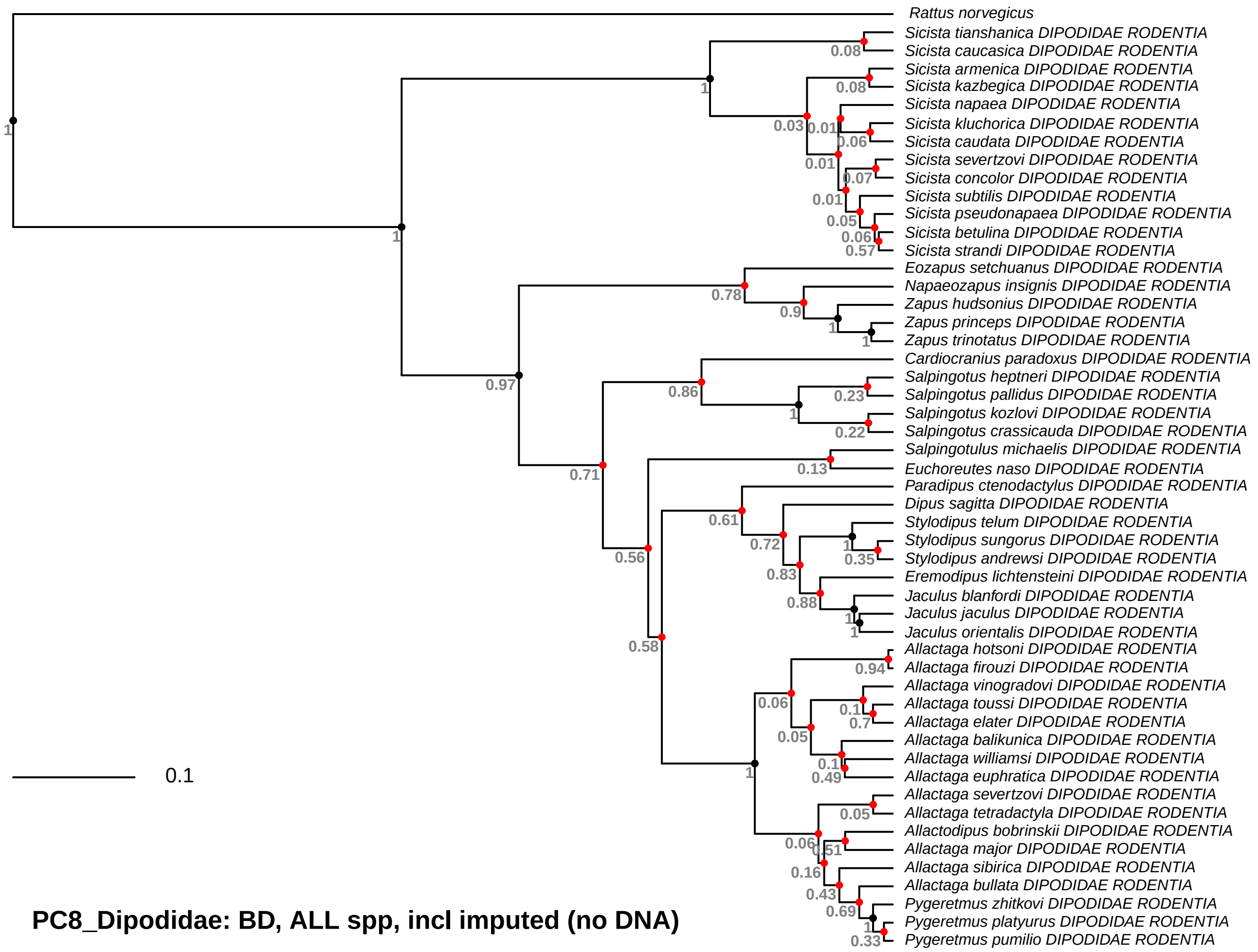
Rattus norvegicus
Sicista tianshanica DIPODIDAE RODENTIA
Sicista caucasica DIPODIDAE RODENTIA
Sicista armenica DIPODIDAE RODENTIA
Sicista kazbegica DIPODIDAE RODENTIA
Sicista napaea DIPODIDAE RODENTIA
Sicista kluchorica DIPODIDAE RODENTIA
Sicista caudata DIPODIDAE RODENTIA
Sicista severtzovi DIPODIDAE RODENTIA
Sicista concolor DIPODIDAE RODENTIA
Sicista subtilis DIPODIDAE RODENTIA
Sicista pseudonapaea DIPODIDAE RODENTIA
Sicista betulina DIPODIDAE RODENTIA
Sicista strandi DIPODIDAE RODENTIA
Eozapus setchuanus DIPODIDAE RODENTIA
Napaeozapus insignis DIPODIDAE RODENTIA
Zapus hudsonius DIPODIDAE RODENTIA
Zapus princeps DIPODIDAE RODENTIA
Zapus trinotatus DIPODIDAE RODENTIA
Cardiocranius paradoxus DIPODIDAE RODENTIA
Salpingotus heptneri DIPODIDAE RODENTIA
Salpingotus pallidus DIPODIDAE RODENTIA
Salpingotus kozlovi DIPODIDAE RODENTIA
Salpingotus crassicauda DIPODIDAE RODENTIA
Salpingotulus michaelis DIPODIDAE RODENTIA
Euchoreutes naso DIPODIDAE RODENTIA
Paradipus ctenodactylus DIPODIDAE RODENTIA
Dipus sagitta DIPODIDAE RODENTIA
Stylodipus telum DIPODIDAE RODENTIA
Stylodipus sungorus DIPODIDAE RODENTIA
Stylodipus andrewsi DIPODIDAE RODENTIA
Eremodipus lichtensteini DIPODIDAE RODENTIA
Jaculus blanfordi DIPODIDAE RODENTIA
Jaculus jaculus DIPODIDAE RODENTIA
Jaculus orientalis DIPODIDAE RODENTIA
Allactaga hotsoni DIPODIDAE RODENTIA
Allactaga firouzi DIPODIDAE RODENTIA
Allactaga vinogradovi DIPODIDAE RODENTIA
Allactaga toussi DIPODIDAE RODENTIA
Allactaga elater DIPODIDAE RODENTIA
Allactaga balikunica DIPODIDAE RODENTIA
Allactaga williamsi DIPODIDAE RODENTIA
Allactaga euphratica DIPODIDAE RODENTIA
Allactaga severtzovi DIPODIDAE RODENTIA
Allactaga tetradactyla DIPODIDAE RODENTIA
Allactodipus bobrinskii DIPODIDAE RODENTIA
Allactaga major DIPODIDAE RODENTIA
Allactaga sibirica DIPODIDAE RODENTIA
Allactaga bullata DIPODIDAE RODENTIA
Pygeretmus zhitkovi DIPODIDAE RODENTIA
Pygeretmus platyurus DIPODIDAE RODENTIA
Pygeretmus pumilio DIPODIDAE RODENTIA
0.1
PC8_Dipodidae: BD, ALL spp, incl imputed (no DNA)

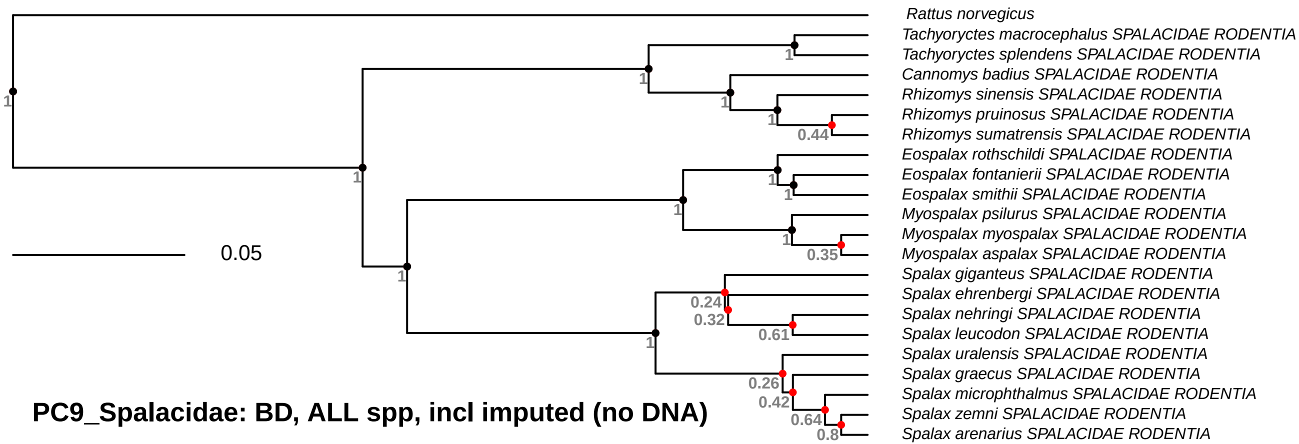

PC9_Spalacidae: BD, ALL spp, incl imputed (no DNA)

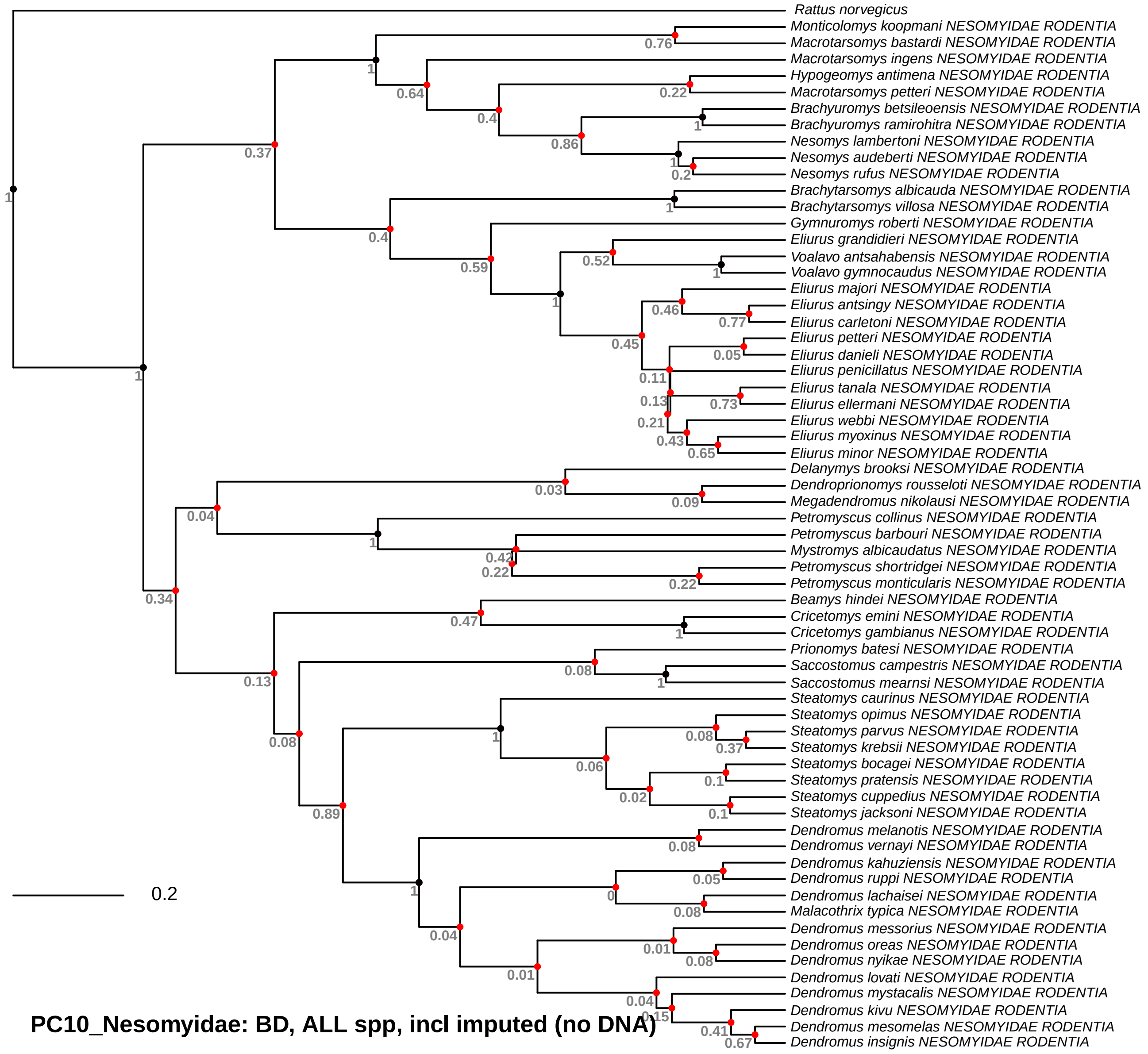
Rattus norvegicus
Monticolomys koopmani NESOMYIDAE RODENTIA
Macrotarsomys bastardi NESOMYIDAE RODENTIA
Macrotarsomys ingens NESOMYIDAE RODENTIA
Hypogeomys antimena NESOMYIDAE RODENTIA
Macrotarsomys petteri NESOMYIDAE RODENTIA
Brachyuromys betsileoensis NESOMYIDAE RODENTIA
Brachyuromys ramirohitra NESOMYIDAE RODENTIA
Nesomys lambertoni NESOMYIDAE RODENTIA
Nesomys audeberti NESOMYIDAE RODENTIA
Nesomys rufus NESOMYIDAE RODENTIA
Brachytarsomys albicauda NESOMYIDAE RODENTIA
Brachytarsomys villosa NESOMYIDAE RODENTIA
Gymnuromys roberti NESOMYIDAE RODENTIA
Eliurus grandidieri NESOMYIDAE RODENTIA
Voalavo antsahabensis NESOMYIDAE RODENTIA
Voalavo gymnocaudus NESOMYIDAE RODENTIA
Eliurus majori NESOMYIDAE RODENTIA
Eliurus antsingy NESOMYIDAE RODENTIA
Eliurus carletoni NESOMYIDAE RODENTIA
Eliurus petteri NESOMYIDAE RODENTIA
Eliurus danieli NESOMYIDAE RODENTIA
Eliurus penicillatus NESOMYIDAE RODENTIA
Eliurus tanala NESOMYIDAE RODENTIA
Eliurus ellermani NESOMYIDAE RODENTIA
Eliurus webbi NESOMYIDAE RODENTIA
Eliurus myoxinus NESOMYIDAE RODENTIA
Eliurus minor NESOMYIDAE RODENTIA
Delanymys brooksi NESOMYIDAE RODENTIA
Dendroprionomys rousseloti NESOMYIDAE RODENTIA
Megadendromus nikolausi NESOMYIDAE RODENTIA
Petromyscus collinus NESOMYIDAE RODENTIA
Petromyscus barbouri NESOMYIDAE RODENTIA
Mystromys albicaudatus NESOMYIDAE RODENTIA
Petromyscus shortridgei NESOMYIDAE RODENTIA
Petromyscus monticularis NESOMYIDAE RODENTIA
Beamys hindei NESOMYIDAE RODENTIA
Cricetomys emini NESOMYIDAE RODENTIA
Cricetomys gambianus NESOMYIDAE RODENTIA
Prionomys batesi NESOMYIDAE RODENTIA
Saccostomus campestris NESOMYIDAE RODENTIA
Saccostomus mearnsi NESOMYIDAE RODENTIA
Steatomys caurinus NESOMYIDAE RODENTIA
Steatomys opimus NESOMYIDAE RODENTIA
Steatomys parvus NESOMYIDAE RODENTIA
Steatomys krebsii NESOMYIDAE RODENTIA
Steatomys bocagei NESOMYIDAE RODENTIA
Steatomys pratensis NESOMYIDAE RODENTIA
Steatomys cuppedius NESOMYIDAE RODENTIA
Steatomys jacksoni NESOMYIDAE RODENTIA
Dendromus melanotis NESOMYIDAE RODENTIA
Dendromus vernayi NESOMYIDAE RODENTIA
Dendromus kahuziensis NESOMYIDAE RODENTIA
Dendromus ruppi NESOMYIDAE RODENTIA
Dendromus lachaisei NESOMYIDAE RODENTIA
Malacothrix typica NESOMYIDAE RODENTIA
Dendromus messorius NESOMYIDAE RODENTIA
Dendromus oreas NESOMYIDAE RODENTIA
Dendromus nyikae NESOMYIDAE RODENTIA
Dendromus lovati NESOMYIDAE RODENTIA
Dendromus mystacalis NESOMYIDAE RODENTIA
Dendromus kivu NESOMYIDAE RODENTIA
Dendromus mesomelas NESOMYIDAE RODENTIA
Dendromus insignis NESOMYIDAE RODENTIA
0.2
PC10_Nesomyidae: BD, ALL spp, incl imputed (no DNA)

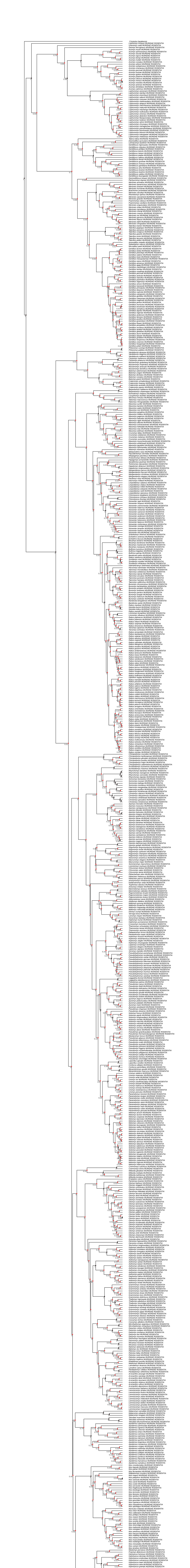

PC11_Muridae: BD, ALL spp, incl imputed (no DNA)

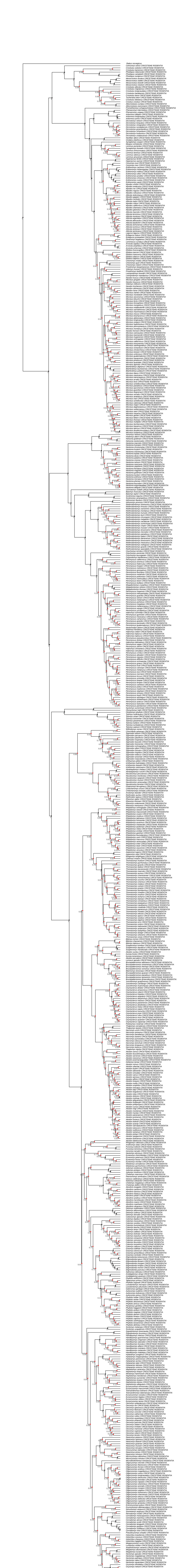

PC12_Cricetidae: BD, ALL spp, incl imputed (no DNA)

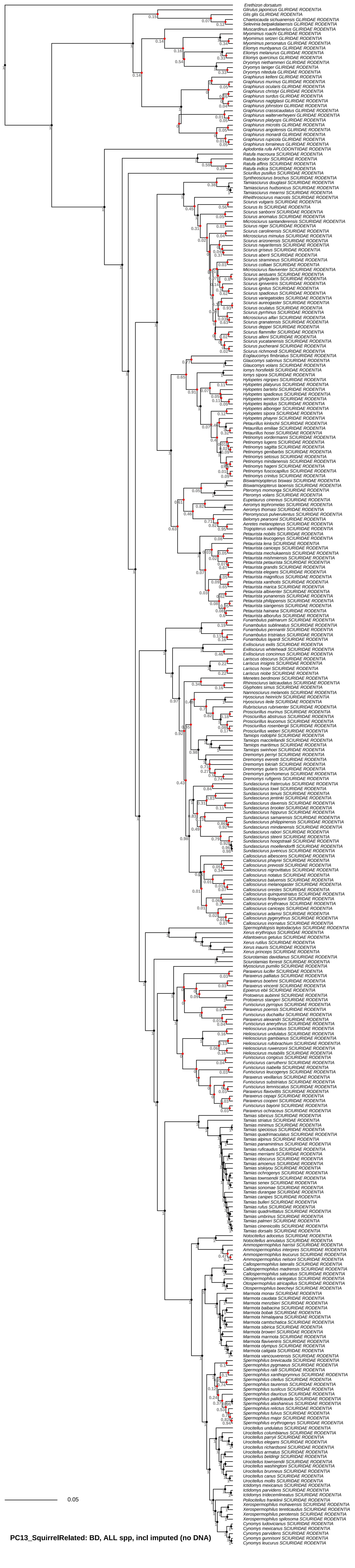

PC13_SquirrelRelated: BD, ALL spp, incl imputed (no DNA)

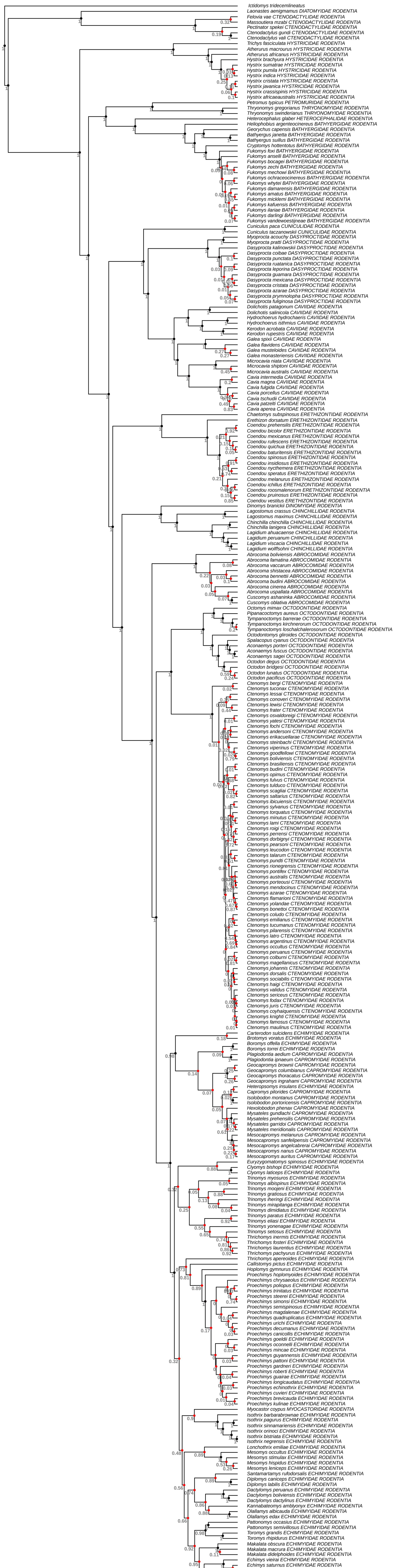

PC14_GuineaPigRelated: BD, ALL spp, incl imputed (no DNA)

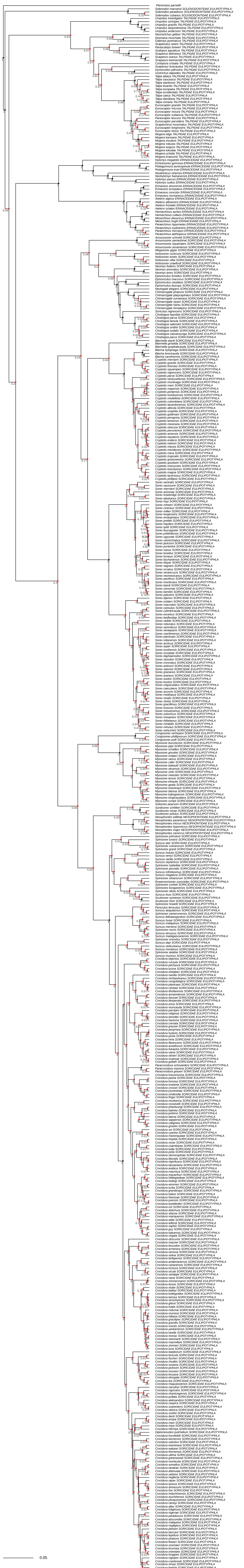

PC15_Eulipotyphla: BD, ALL spp, incl imputed (no DNA)

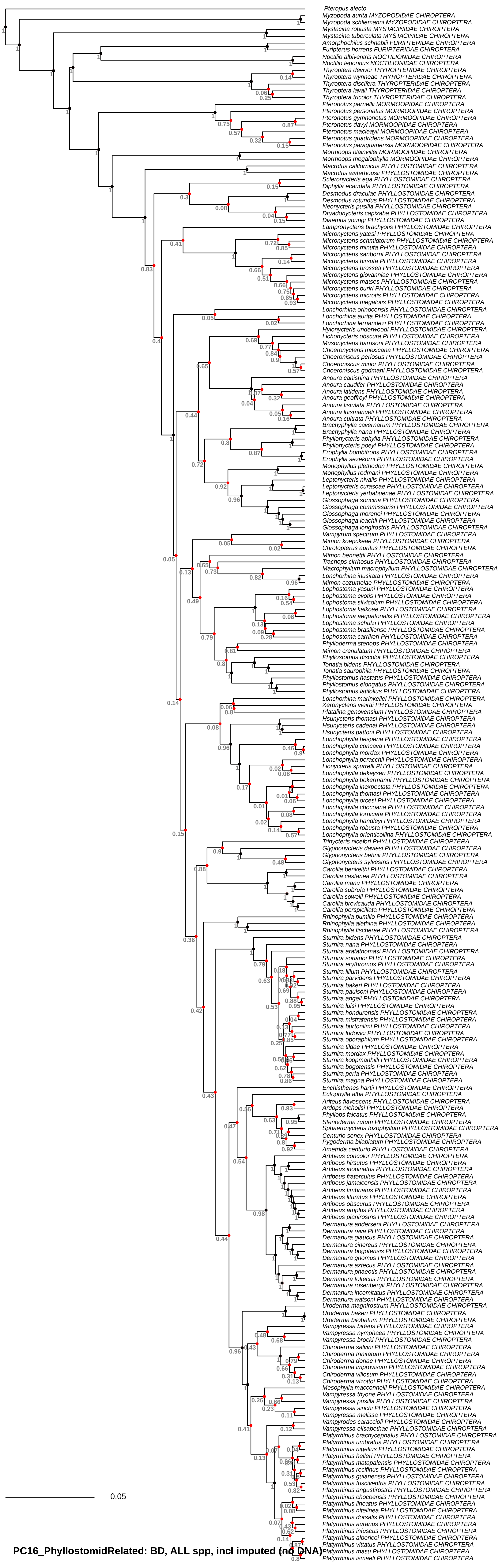

PC16_PhyllostomidRelated: BD, ALL spp, incl imputed (no DNA)

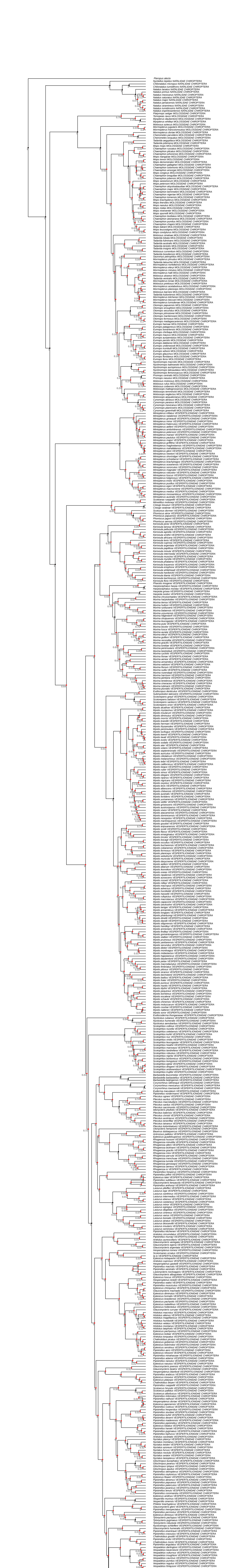

PC17_VespertilionidRelated: BD, ALL spp, incl imputed (no DNA)

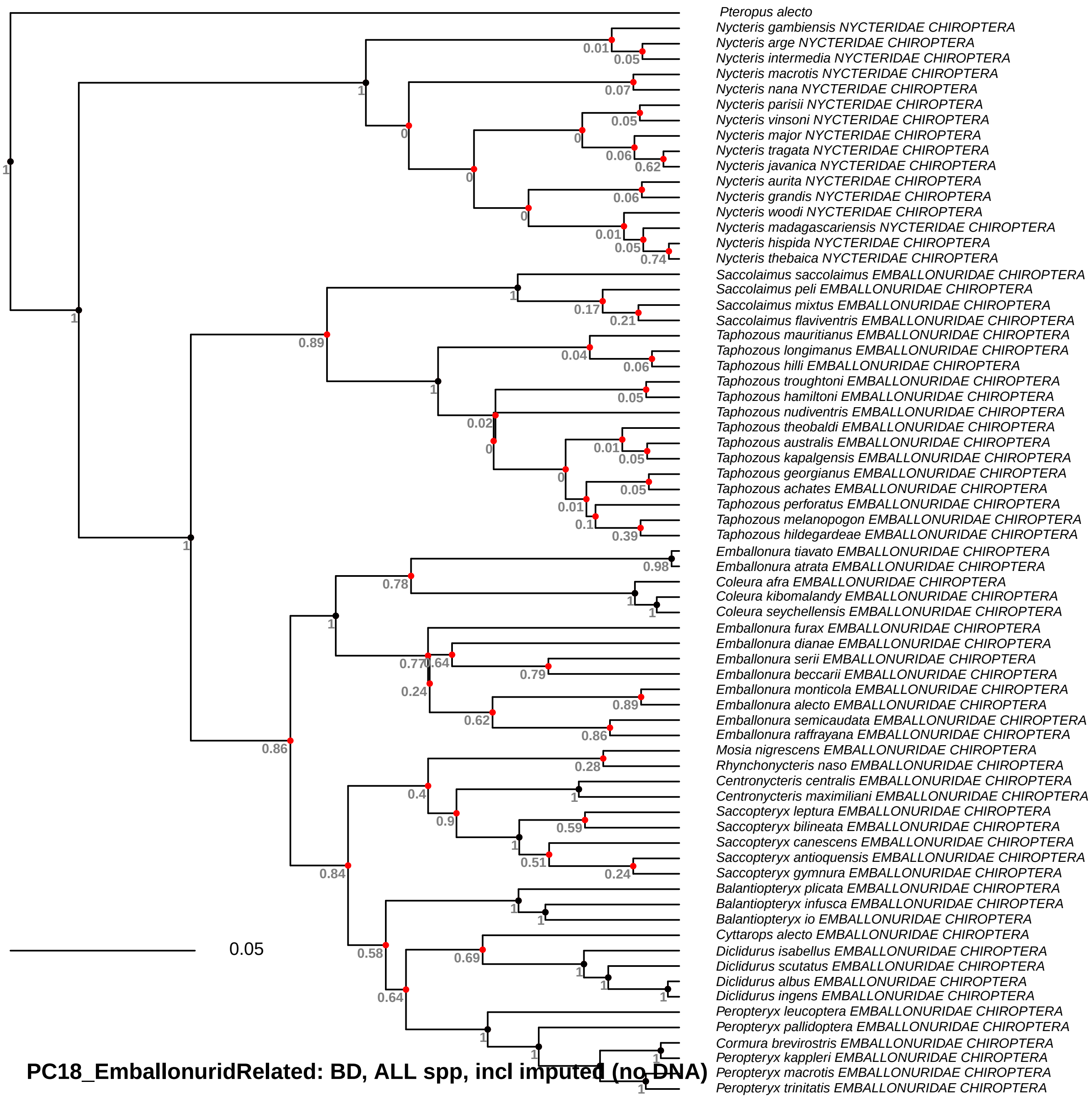

PC18_EmballonuridRelated: BD, ALL spp, incl imputed (no DNA)

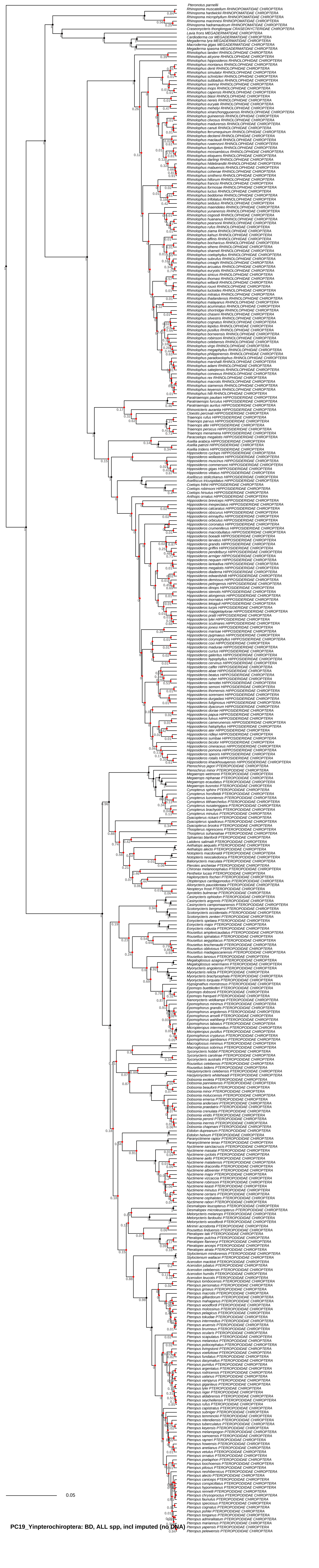

PC19_Yinpterochiroptera: BD, ALL spp, incl imputed (no DNA)

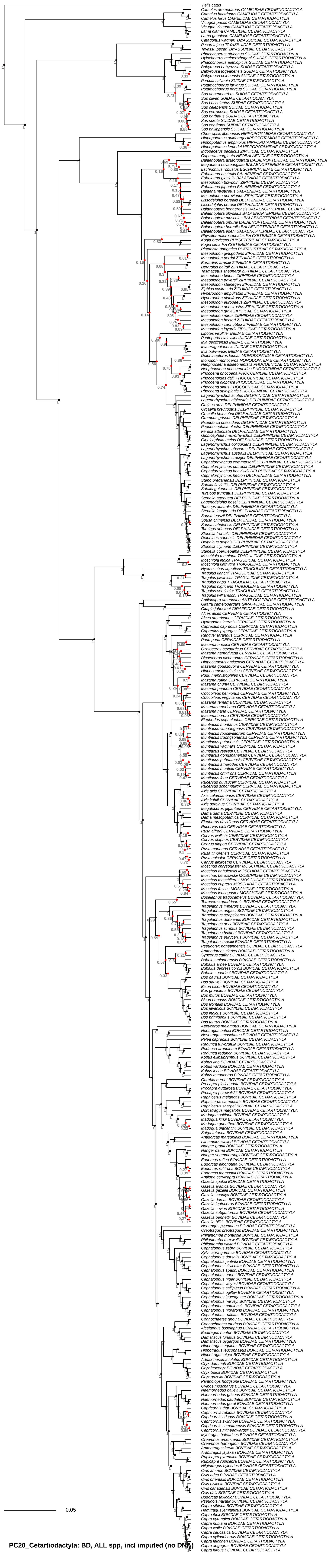

PC20_Cetartiodactyla: BD, ALL spp, incl imputed (no DNA)

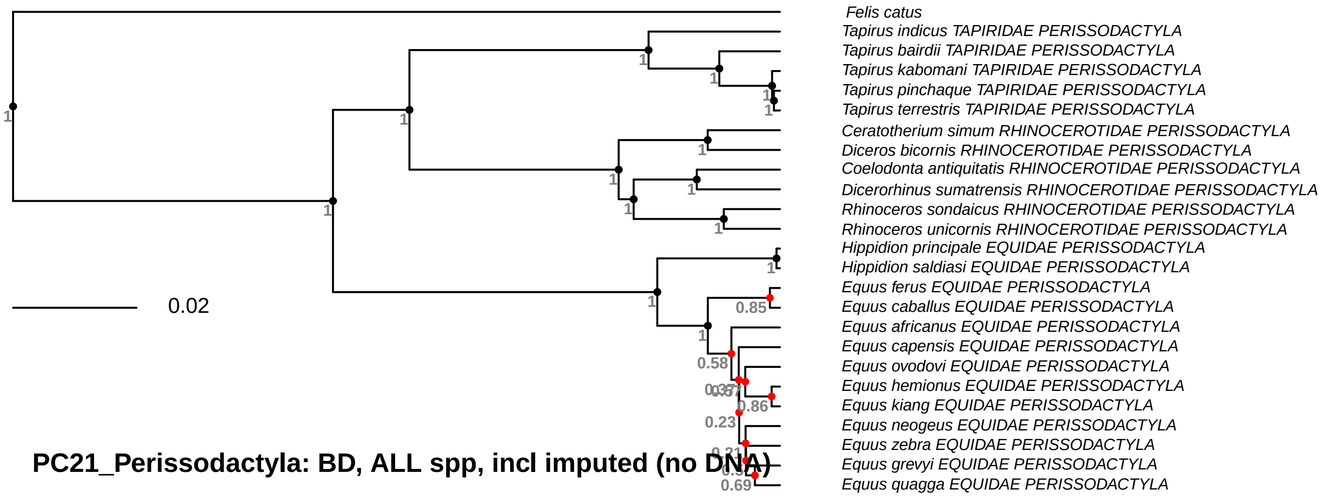

PC21_Perissodactyla: BD, ALL spp, incl imputed (no DNA)

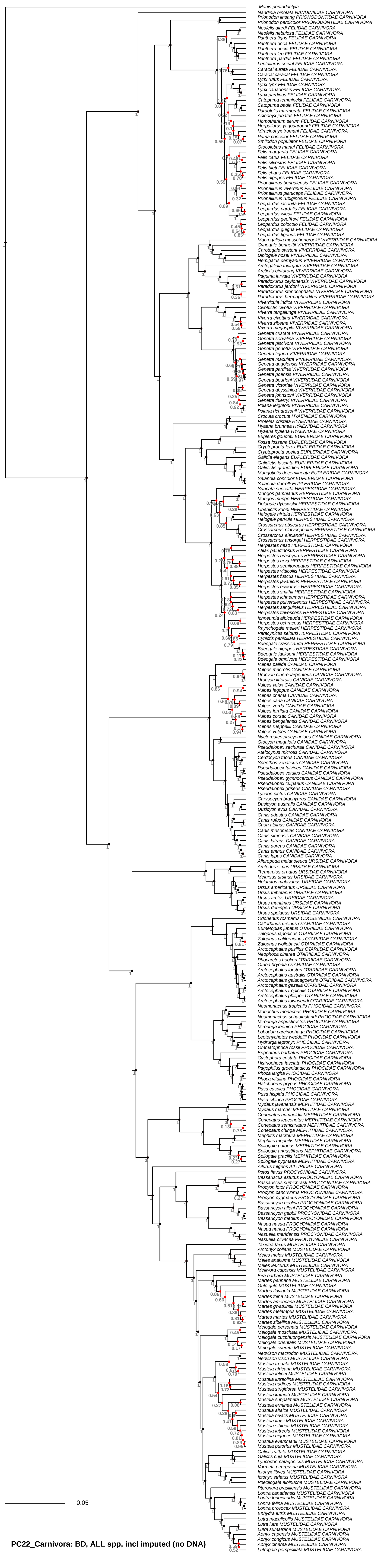

PC22_Carnivora: BD, ALL spp, incl imputed (no DNA)

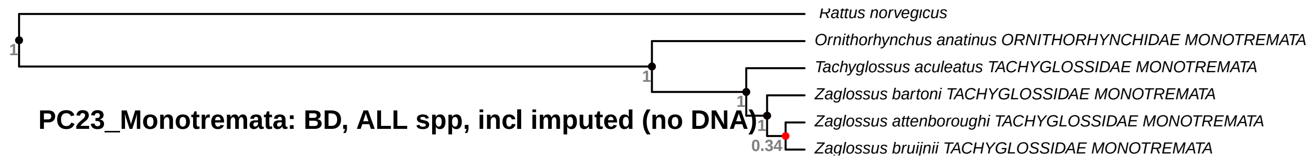
Rattus norvegicus
Ornithorhynchus anatinus ORNITHORHYNCHIDAE MONOTREMATA
Tachyglossus aculeatus TACHYGLOSSIDAE MONOTREMATA
Zaglossus bartoni TACHYGLOSSIDAE MONOTREMATA
Zaglossus attenboroughi TACHYGLOSSIDAE MONOTREMATA
Zaglossus bruijnii TACHYGLOSSIDAE MONOTREMATA
PC23_Monotremata: BD, ALL spp, incl imputed (no DNA)
1
1
1
1
0.34

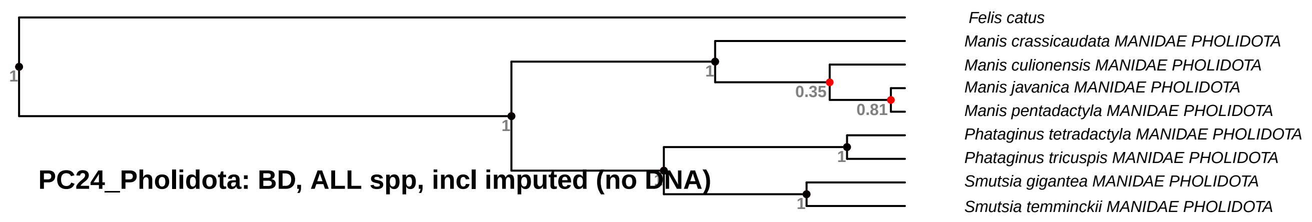
Felis catus
Manis crassicaudata MANIDAE PHOLIDOTA
Manis culionensis MANIDAE PHOLIDOTA
Manis javanica MANIDAE PHOLIDOTA
Manis pentadactyla MANIDAE PHOLIDOTA
Phataginus tetradactyla MANIDAE PHOLIDOTA
Phataginus tricuspis MANIDAE PHOLIDOTA
Smutsia gigantea MANIDAE PHOLIDOTA
Smutsia temminckii MANIDAE PHOLIDOTA
1
1
1
0.35
0.81
1
1
1
PC24_Pholidota: BD, ALL spp, incl imputed (no DNA)

PC25_Dermoptera: BD, ALL spp, incl imputed (no DNA)

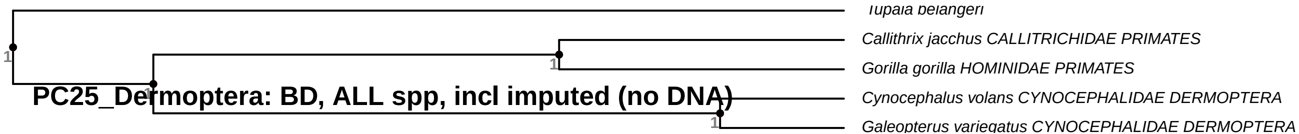

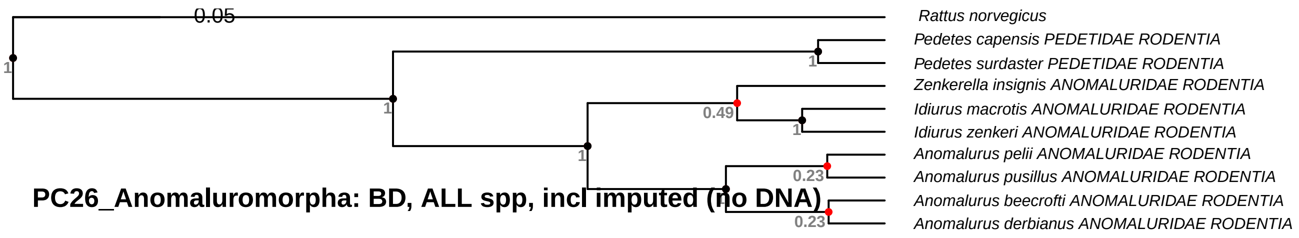
0.05
Rattus norvegicus
Pedetes capensis PEDETIDAE RODENTIA
Pedetes surdaster PEDETIDAE RODENTIA
Zenkerella insignis ANOMALURIDAE RODENTIA
Idiurus macrotis ANOMALURIDAE RODENTIA
Idiurus zenkeri ANOMALURIDAE RODENTIA
Anomalurus pelii ANOMALURIDAE RODENTIA
Anomalurus pusillus ANOMALURIDAE RODENTIA
Anomalurus beecrofti ANOMALURIDAE RODENTIA
Anomalurus derbianus ANOMALURIDAE RODENTIA
1
1
1
1
0.49
1
0.23
0.23
PC26_Anomaluromorpha: BD, ALL spp, incl imputed (no DNA)

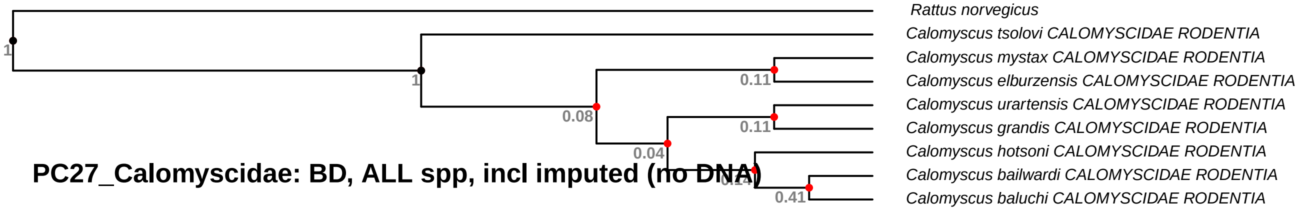
PC27_Calomyscidae: BD, ALL spp, incl imputed (no DNA)
Rattus norvegicus
Calomyscus tsolovi CALOMYSCIDAE RODENTIA
Calomyscus mystax CALOMYSCIDAE RODENTIA
Calomyscus elburzensis CALOMYSCIDAE RODENTIA
Calomyscus urartensis CALOMYSCIDAE RODENTIA
Calomyscus grandis CALOMYSCIDAE RODENTIA
Calomyscus hotsoni CALOMYSCIDAE RODENTIA
Calomyscus bailwardi CALOMYSCIDAE RODENTIA
Calomyscus baluchi CALOMYSCIDAE RODENTIA
1
1
0.08
0.11
0.11
0.04
0.41

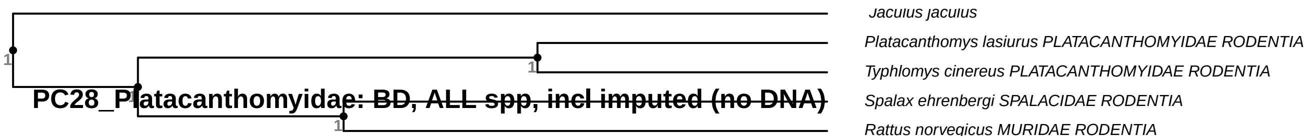
PC28_Platacanthomyidae: BD, ALL spp, incl imputed (no DNA)
Jaculus jaculus
Platacanthomys lasiurus PLATACANTHOMYIDAE RODENTIA
Typhlomys cinereus PLATACANTHOMYIDAE RODENTIA
Spalax ehrenbergi SPALACIDAE RODENTIA
Rattus norvegicus MURIDAE RODENTIA
1
1
1
1
