# Supplementary material for: Inferring the mammal tree: Species-level sets of phylogenies for questions in ecology, evolution, and conservation
Source: PLoS Biol. 2019 Dec 4;17(12):e3000494. doi: 10.1371/journal.pbio.3000494 (PMC6892540; doi:10.1371/journal.pbio.3000494)
Supplement: S4 Data — Relative time results (i.e., not yet time scaled) presented as MCC trees for each of the patch clade runs, both as nexus and PDF plots, and details of the species and gene sampling in each patch clade. Dryad data: https://doi.org/10.5061/dryad.tb03d03. MCC, maximum clade credibility. (ZIP) [file pbio.3000494.s024.zip › S4_Data.zip!/Data_S5_patchClade_results_and_MCC/PatchClades_MCC_DNAonly-TopoFree_mean/postburn10k_BDvr_FREE_MCC_PC18_EmballonuridRelated_plot.pdf]

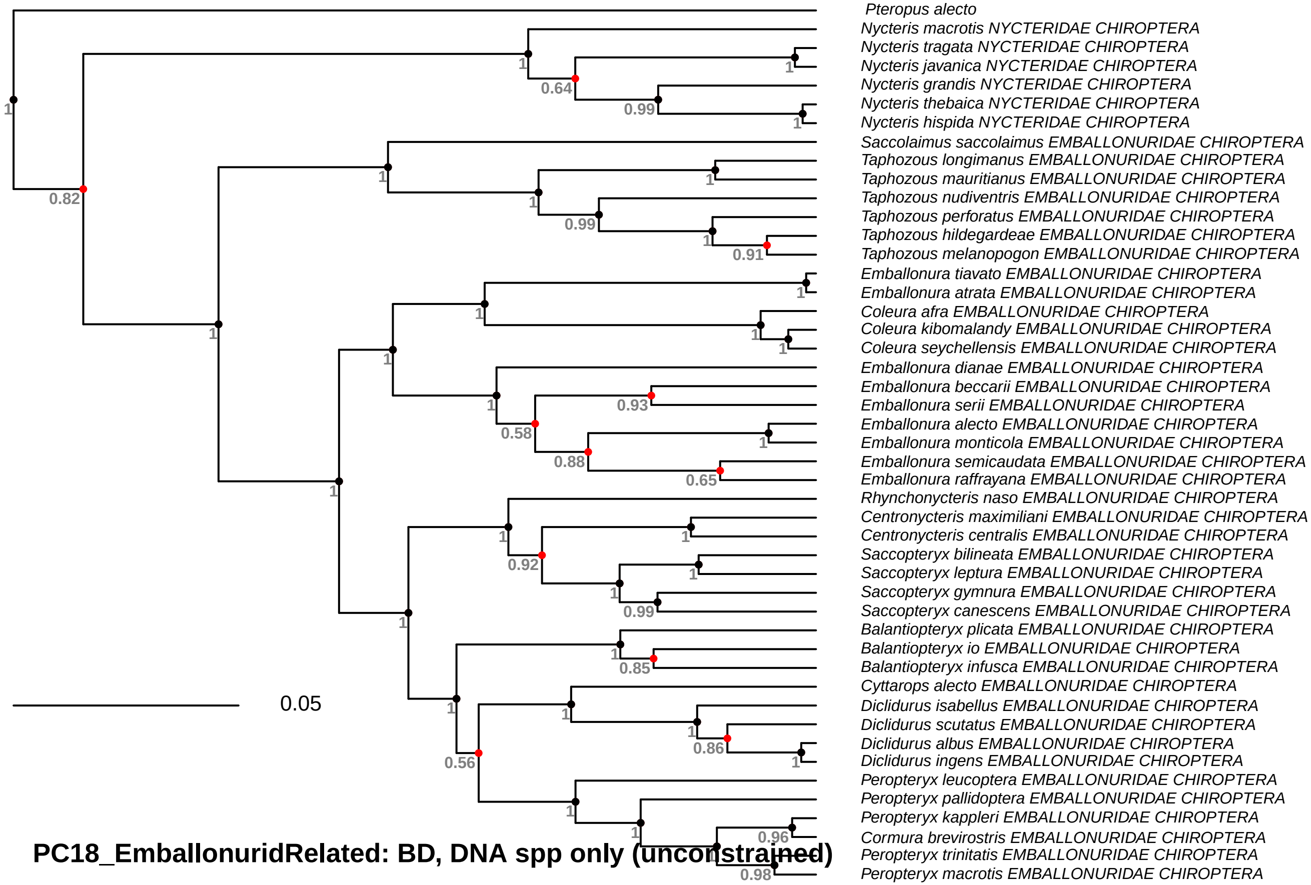
Pteropus alecto
Nycteris macrotis NYCTERIDAE CHIROPTERA
Nycteris tragata NYCTERIDAE CHIROPTERA
Nycteris javanica NYCTERIDAE CHIROPTERA
Nycteris grandis NYCTERIDAE CHIROPTERA
Nycteris thebaica NYCTERIDAE CHIROPTERA
Nycteris hispida NYCTERIDAE CHIROPTERA
Saccolaimus saccolaimus EMBALLONURIDAE CHIROPTERA
Taphozous longimanus EMBALLONURIDAE CHIROPTERA
Taphozous mauritianus EMBALLONURIDAE CHIROPTERA
Taphozous nudiventris EMBALLONURIDAE CHIROPTERA
Taphozous perforatus EMBALLONURIDAE CHIROPTERA
Taphozous hildegardeae EMBALLONURIDAE CHIROPTERA
Taphozous melanopogon EMBALLONURIDAE CHIROPTERA
Emballonura tiavato EMBALLONURIDAE CHIROPTERA
Emballonura atrata EMBALLONURIDAE CHIROPTERA
Coleura afra EMBALLONURIDAE CHIROPTERA
Coleura kibomalandy EMBALLONURIDAE CHIROPTERA
Coleura seychellensis EMBALLONURIDAE CHIROPTERA
Emballonura dianae EMBALLONURIDAE CHIROPTERA
Emballonura beccarii EMBALLONURIDAE CHIROPTERA
Emballonura serii EMBALLONURIDAE CHIROPTERA
Emballonura alecto EMBALLONURIDAE CHIROPTERA
Emballonura monticola EMBALLONURIDAE CHIROPTERA
Emballonura semicaudata EMBALLONURIDAE CHIROPTERA
Emballonura raffrayana EMBALLONURIDAE CHIROPTERA
Rhynchonycteris naso EMBALLONURIDAE CHIROPTERA
Centronycteris maximiliani EMBALLONURIDAE CHIROPTERA
Centronycteris centralis EMBALLONURIDAE CHIROPTERA
Saccopteryx bilineata EMBALLONURIDAE CHIROPTERA
Saccopteryx leptura EMBALLONURIDAE CHIROPTERA
Saccopteryx gymnura EMBALLONURIDAE CHIROPTERA
Saccopteryx canescens EMBALLONURIDAE CHIROPTERA
Balantiopteryx plicata EMBALLONURIDAE CHIROPTERA
Balantiopteryx io EMBALLONURIDAE CHIROPTERA
Balantiopteryx infusca EMBALLONURIDAE CHIROPTERA
Cyttarops alecto EMBALLONURIDAE CHIROPTERA
Diclidurus isabellus EMBALLONURIDAE CHIROPTERA
Diclidurus scutatus EMBALLONURIDAE CHIROPTERA
Diclidurus albus EMBALLONURIDAE CHIROPTERA
Diclidurus ingens EMBALLONURIDAE CHIROPTERA
Peropteryx leucoptera EMBALLONURIDAE CHIROPTERA
Peropteryx pallidoptera EMBALLONURIDAE CHIROPTERA
Peropteryx kappleri EMBALLONURIDAE CHIROPTERA
Cormura brevirostris EMBALLONURIDAE CHIROPTERA
Peropteryx trinitatis EMBALLONURIDAE CHIROPTERA
Peropteryx macrotis EMBALLONURIDAE CHIROPTERA
0.05
PC18_EmballonuridRelated: BD, DNA spp only (unconstrained)
