# Supplementary material for: Inferring the mammal tree: Species-level sets of phylogenies for questions in ecology, evolution, and conservation
Source: PLoS Biol. 2019 Dec 4;17(12):e3000494. doi: 10.1371/journal.pbio.3000494 (PMC6892540; doi:10.1371/journal.pbio.3000494)
Supplement: S4 Data — Relative time results (i.e., not yet time scaled) presented as MCC trees for each of the patch clade runs, both as nexus and PDF plots, and details of the species and gene sampling in each patch clade. Dryad data: https://doi.org/10.5061/dryad.tb03d03. MCC, maximum clade credibility. (ZIP) [file pbio.3000494.s024.zip › S4_Data.zip!/Data_S5_patchClade_results_and_MCC/PatchClades_MCC_DNAonly-TopoFree_mean/postburn10k_BDvr_FREE_MCC_PC6_Lagomorpha_plot.pdf]

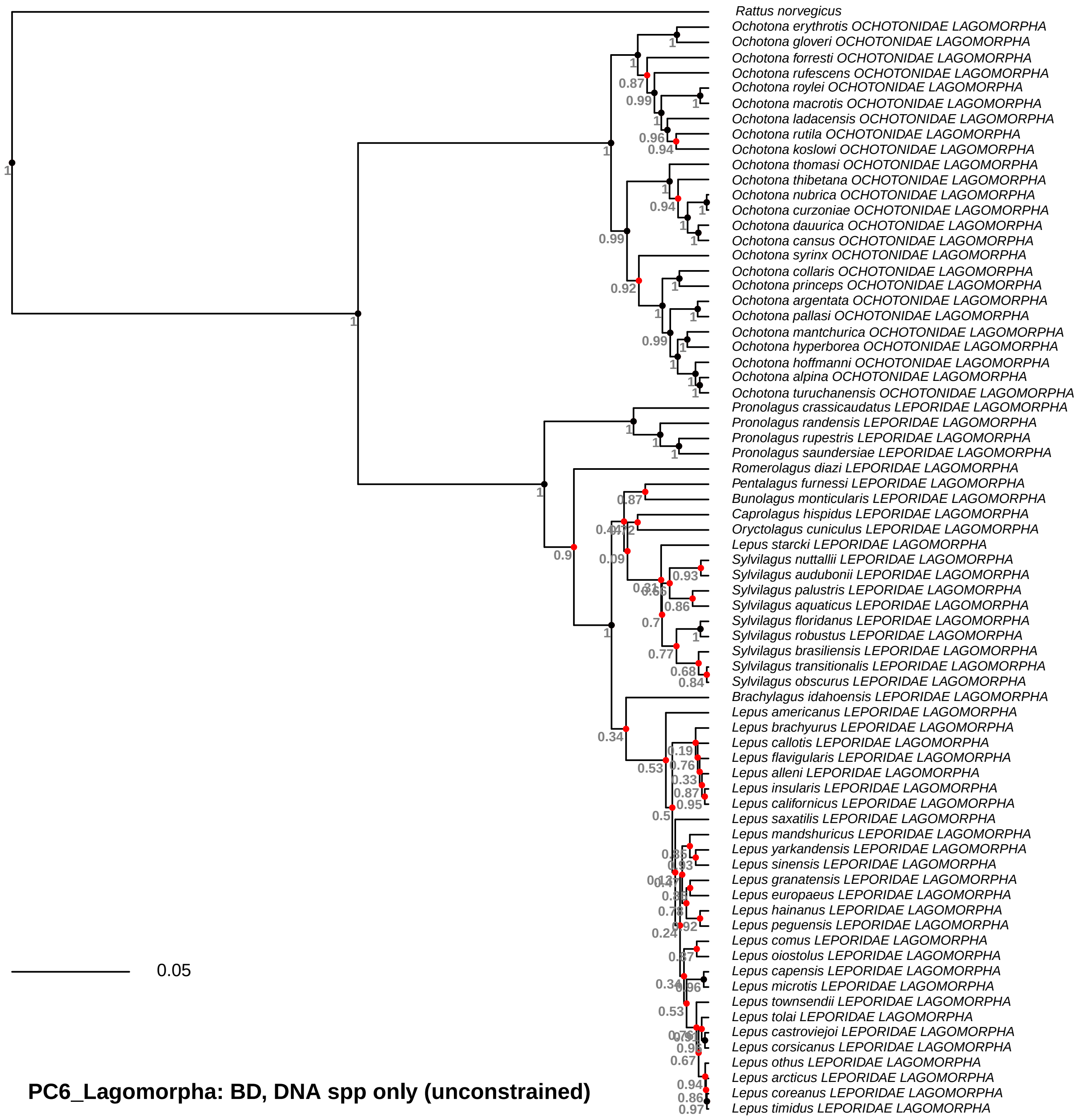
Rattus norvegicus
Ochotona erythrotis OCHOTONIDAE LAGOMORPHA
Ochotona gloveri OCHOTONIDAE LAGOMORPHA
Ochotona forresti OCHOTONIDAE LAGOMORPHA
Ochotona rufescens OCHOTONIDAE LAGOMORPHA
Ochotona roylei OCHOTONIDAE LAGOMORPHA
Ochotona macrotis OCHOTONIDAE LAGOMORPHA
Ochotona ladacensis OCHOTONIDAE LAGOMORPHA
Ochotona rutila OCHOTONIDAE LAGOMORPHA
Ochotona koslowi OCHOTONIDAE LAGOMORPHA
Ochotona thomasi OCHOTONIDAE LAGOMORPHA
Ochotona thibetana OCHOTONIDAE LAGOMORPHA
Ochotona nubrica OCHOTONIDAE LAGOMORPHA
Ochotona curzoniae OCHOTONIDAE LAGOMORPHA
Ochotona dauurica OCHOTONIDAE LAGOMORPHA
Ochotona cansus OCHOTONIDAE LAGOMORPHA
Ochotona syrinx OCHOTONIDAE LAGOMORPHA
Ochotona collaris OCHOTONIDAE LAGOMORPHA
Ochotona princeps OCHOTONIDAE LAGOMORPHA
Ochotona argentata OCHOTONIDAE LAGOMORPHA
Ochotona pallasi OCHOTONIDAE LAGOMORPHA
Ochotona mantchurica OCHOTONIDAE LAGOMORPHA
Ochotona hyperborea OCHOTONIDAE LAGOMORPHA
Ochotona hoffmanni OCHOTONIDAE LAGOMORPHA
Ochotona alpina OCHOTONIDAE LAGOMORPHA
Ochotona turuchanensis OCHOTONIDAE LAGOMORPHA
Pronolagus crassicaudatus LEPORIDAE LAGOMORPHA
Pronolagus randensis LEPORIDAE LAGOMORPHA
Pronolagus rupestris LEPORIDAE LAGOMORPHA
Pronolagus saundersiae LEPORIDAE LAGOMORPHA
Romerolagus diazi LEPORIDAE LAGOMORPHA
Pentalagus furnessi LEPORIDAE LAGOMORPHA
Bunolagus monticularis LEPORIDAE LAGOMORPHA
Caprolagus hispidus LEPORIDAE LAGOMORPHA
Oryctolagus cuniculus LEPORIDAE LAGOMORPHA
Lepus starcki LEPORIDAE LAGOMORPHA
Sylvilagus nuttallii LEPORIDAE LAGOMORPHA
Sylvilagus audubonii LEPORIDAE LAGOMORPHA
Sylvilagus palustris LEPORIDAE LAGOMORPHA
Sylvilagus aquaticus LEPORIDAE LAGOMORPHA
Sylvilagus floridanus LEPORIDAE LAGOMORPHA
Sylvilagus robustus LEPORIDAE LAGOMORPHA
Sylvilagus brasiliensis LEPORIDAE LAGOMORPHA
Sylvilagus transitionalis LEPORIDAE LAGOMORPHA
Sylvilagus obscurus LEPORIDAE LAGOMORPHA
Brachylagus idahoensis LEPORIDAE LAGOMORPHA
Lepus americanus LEPORIDAE LAGOMORPHA
Lepus brachyurus LEPORIDAE LAGOMORPHA
Lepus callotis LEPORIDAE LAGOMORPHA
Lepus flavigularis LEPORIDAE LAGOMORPHA
Lepus alleni LEPORIDAE LAGOMORPHA
Lepus insularis LEPORIDAE LAGOMORPHA
Lepus californicus LEPORIDAE LAGOMORPHA
Lepus saxatilis LEPORIDAE LAGOMORPHA
Lepus mandshuricus LEPORIDAE LAGOMORPHA
Lepus yarkandensis LEPORIDAE LAGOMORPHA
Lepus sinensis LEPORIDAE LAGOMORPHA
Lepus granatensis LEPORIDAE LAGOMORPHA
Lepus europaeus LEPORIDAE LAGOMORPHA
Lepus hainanus LEPORIDAE LAGOMORPHA
Lepus peguensis LEPORIDAE LAGOMORPHA
Lepus comus LEPORIDAE LAGOMORPHA
Lepus oiostolus LEPORIDAE LAGOMORPHA
Lepus capensis LEPORIDAE LAGOMORPHA
Lepus microtis LEPORIDAE LAGOMORPHA
Lepus townsendii LEPORIDAE LAGOMORPHA
Lepus tolai LEPORIDAE LAGOMORPHA
Lepus castroviejoi LEPORIDAE LAGOMORPHA
Lepus corsicanus LEPORIDAE LAGOMORPHA
Lepus othus LEPORIDAE LAGOMORPHA
Lepus arcticus LEPORIDAE LAGOMORPHA
Lepus coreanus LEPORIDAE LAGOMORPHA
Lepus timidus LEPORIDAE LAGOMORPHA
0.05
PC6_Lagomorpha: BD, DNA spp only (unconstrained)
