# Supplementary material for: Inferring the mammal tree: Species-level sets of phylogenies for questions in ecology, evolution, and conservation
Source: PLoS Biol. 2019 Dec 4;17(12):e3000494. doi: 10.1371/journal.pbio.3000494 (PMC6892540; doi:10.1371/journal.pbio.3000494)

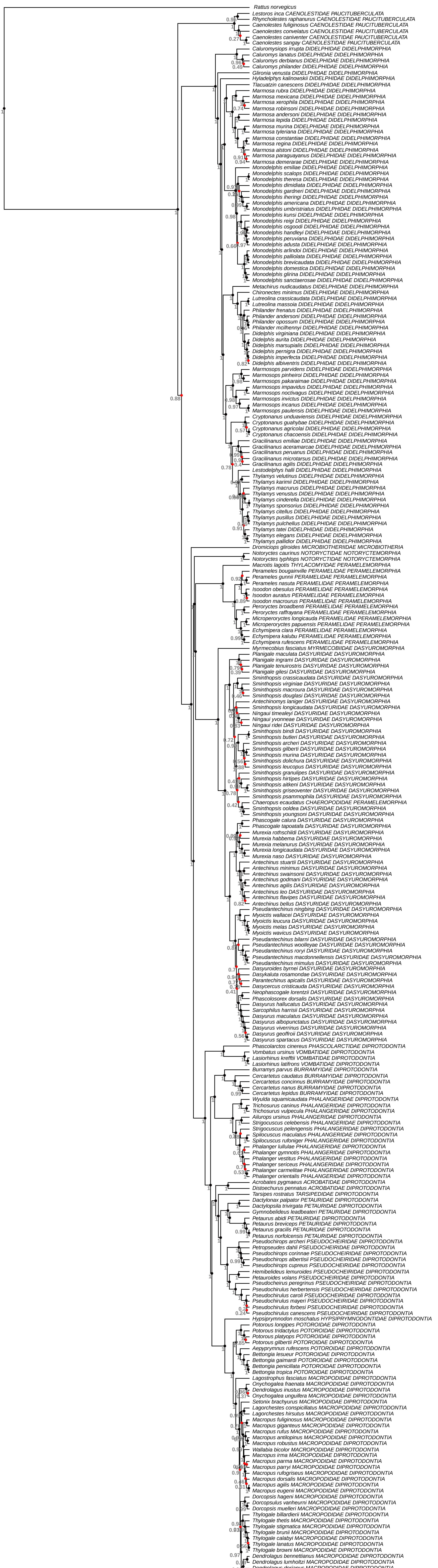

PC1_Marsupials: BD, DNA spp only (unconstrained)

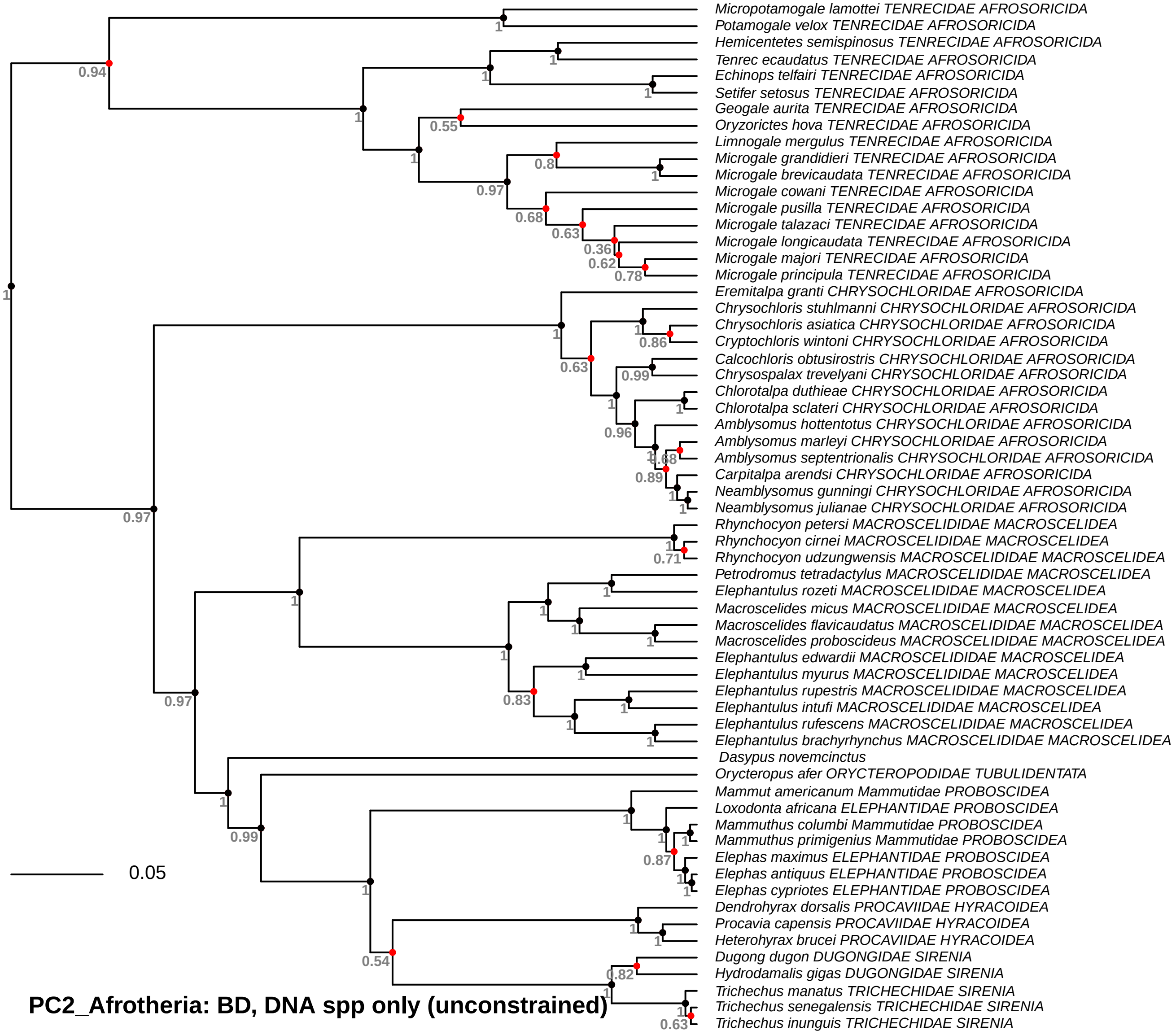
Micropotamogale lamottei TENRECIDAE AFROSORICIDA
Potamogale velox TENRECIDAE AFROSORICIDA
Hemicentetes semispinosus TENRECIDAE AFROSORICIDA
Tenrec ecaudatus TENRECIDAE AFROSORICIDA
Echinops telfairi TENRECIDAE AFROSORICIDA
Setifer setosus TENRECIDAE AFROSORICIDA
Geogale aurita TENRECIDAE AFROSORICIDA
Oryzorictes hova TENRECIDAE AFROSORICIDA
Limnogale mergulus TENRECIDAE AFROSORICIDA
Microgale grandidieri TENRECIDAE AFROSORICIDA
Microgale brevicaudata TENRECIDAE AFROSORICIDA
Microgale cowani TENRECIDAE AFROSORICIDA
Microgale pusilla TENRECIDAE AFROSORICIDA
Microgale talazaci TENRECIDAE AFROSORICIDA
Microgale longicaudata TENRECIDAE AFROSORICIDA
Microgale majori TENRECIDAE AFROSORICIDA
Microgale principula TENRECIDAE AFROSORICIDA
Eremitalpa granti CHRYSOCHLORIDAE AFROSORICIDA
Chrysochloris stuhlmanni CHRYSOCHLORIDAE AFROSORICIDA
Chrysochloris asiatica CHRYSOCHLORIDAE AFROSORICIDA
Cryptochloris wintoni CHRYSOCHLORIDAE AFROSORICIDA
Calcochloris obtusirostris CHRYSOCHLORIDAE AFROSORICIDA
Chrysospalax trevelyani CHRYSOCHLORIDAE AFROSORICIDA
Chlorotalpa duthieae CHRYSOCHLORIDAE AFROSORICIDA
Chlorotalpa sclateri CHRYSOCHLORIDAE AFROSORICIDA
Amblysomus hottentotus CHRYSOCHLORIDAE AFROSORICIDA
Amblysomus marleyi CHRYSOCHLORIDAE AFROSORICIDA
Amblysomus septentrionalis CHRYSOCHLORIDAE AFROSORICIDA
Carpitalpa arendsi CHRYSOCHLORIDAE AFROSORICIDA
Neamblysomus gunningi CHRYSOCHLORIDAE AFROSORICIDA
Neamblysomus julianae CHRYSOCHLORIDAE AFROSORICIDA
Rhynchocyon petersi MACROSCELIDIDAE MACROSCELIDEA
Rhynchocyon cirnei MACROSCELIDIDAE MACROSCELIDEA
Rhynchocyon udzungwensis MACROSCELIDIDAE MACROSCELIDEA
Petrodromus tetradactylus MACROSCELIDIDAE MACROSCELIDEA
Elephantulus rozeti MACROSCELIDIDAE MACROSCELIDEA
Macroscelides micus MACROSCELIDIDAE MACROSCELIDEA
Macroscelides flavicaudatus MACROSCELIDIDAE MACROSCELIDEA
Macroscelides proboscideus MACROSCELIDIDAE MACROSCELIDEA
Elephantulus edwardii MACROSCELIDIDAE MACROSCELIDEA
Elephantulus myurus MACROSCELIDIDAE MACROSCELIDEA
Elephantulus rupestris MACROSCELIDIDAE MACROSCELIDEA
Elephantulus intufi MACROSCELIDIDAE MACROSCELIDEA
Elephantulus rufescens MACROSCELIDIDAE MACROSCELIDEA
Elephantulus brachyrhynchus MACROSCELIDIDAE MACROSCELIDEA
Dasypus novemcinctus
Orycteropus afer ORYCTEROPODIDAE TUBULIDENTATA
Mammut americanum Mammutidae PROBOSCIDEA
Loxodonta africana ELEPHANTIDAE PROBOSCIDEA
Mammuthus columbi Mammutidae PROBOSCIDEA
Mammuthus primigenius Mammutidae PROBOSCIDEA
Elephas maximus ELEPHANTIDAE PROBOSCIDEA
Elephas antiquus ELEPHANTIDAE PROBOSCIDEA
Elephas cypriotes ELEPHANTIDAE PROBOSCIDEA
Dendrohyrax dorsalis PROCAVIIDAE HYRACOIDEA
Procavia capensis PROCAVIIDAE HYRACOIDEA
Heterohyrax brucei PROCAVIIDAE HYRACOIDEA
Dugong dugon DUGONGIDAE SIRENIA
Hydrodamalis gigas DUGONGIDAE SIRENIA
Trichechus manatus TRICHECHIDAE SIRENIA
Trichechus senegalensis TRICHECHIDAE SIRENIA
Trichechus inunguis TRICHECHIDAE SIRENIA
0.05
PC2_Afrotheria: BD, DNA spp only (unconstrained)

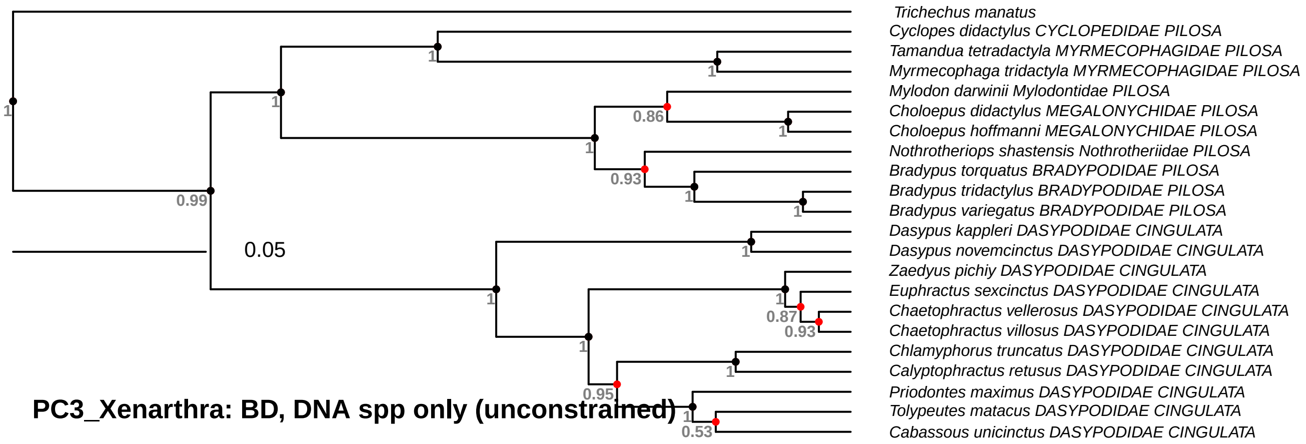

PC3_Xenarthra: BD, DNA spp only (unconstrained)

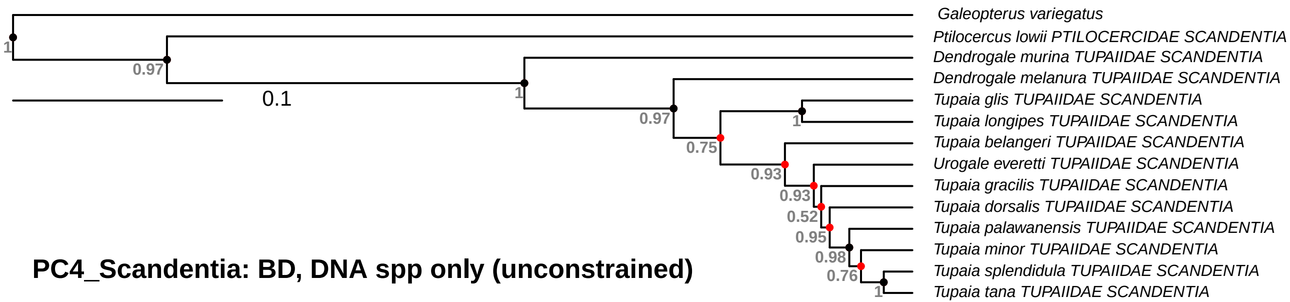

PC4_Scandentia: BD, DNA spp only (unconstrained)

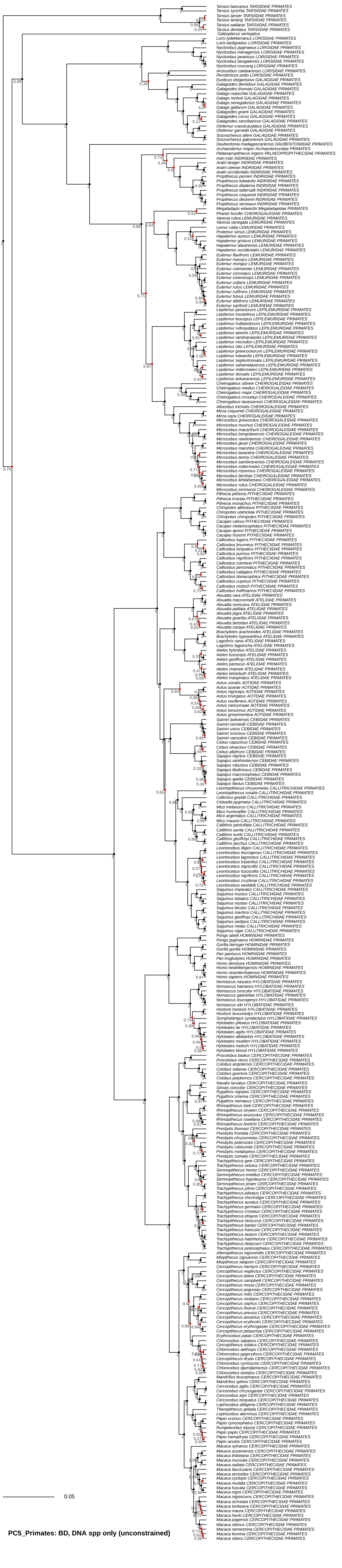

PC5_Primates: BD, DNA spp only (unconstrained)

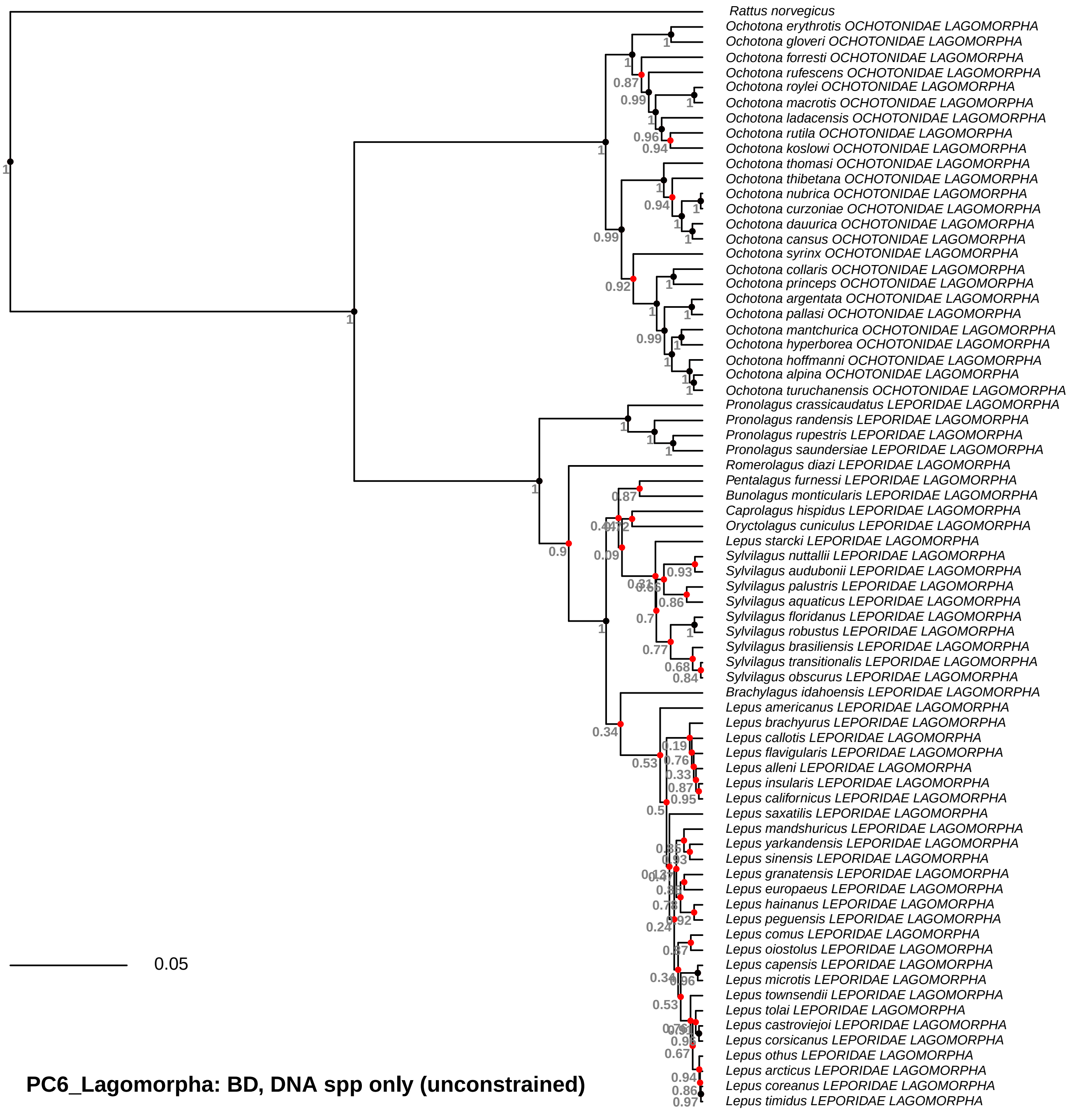

PC6_Lagomorpha: BD, DNA spp only (unconstrained)

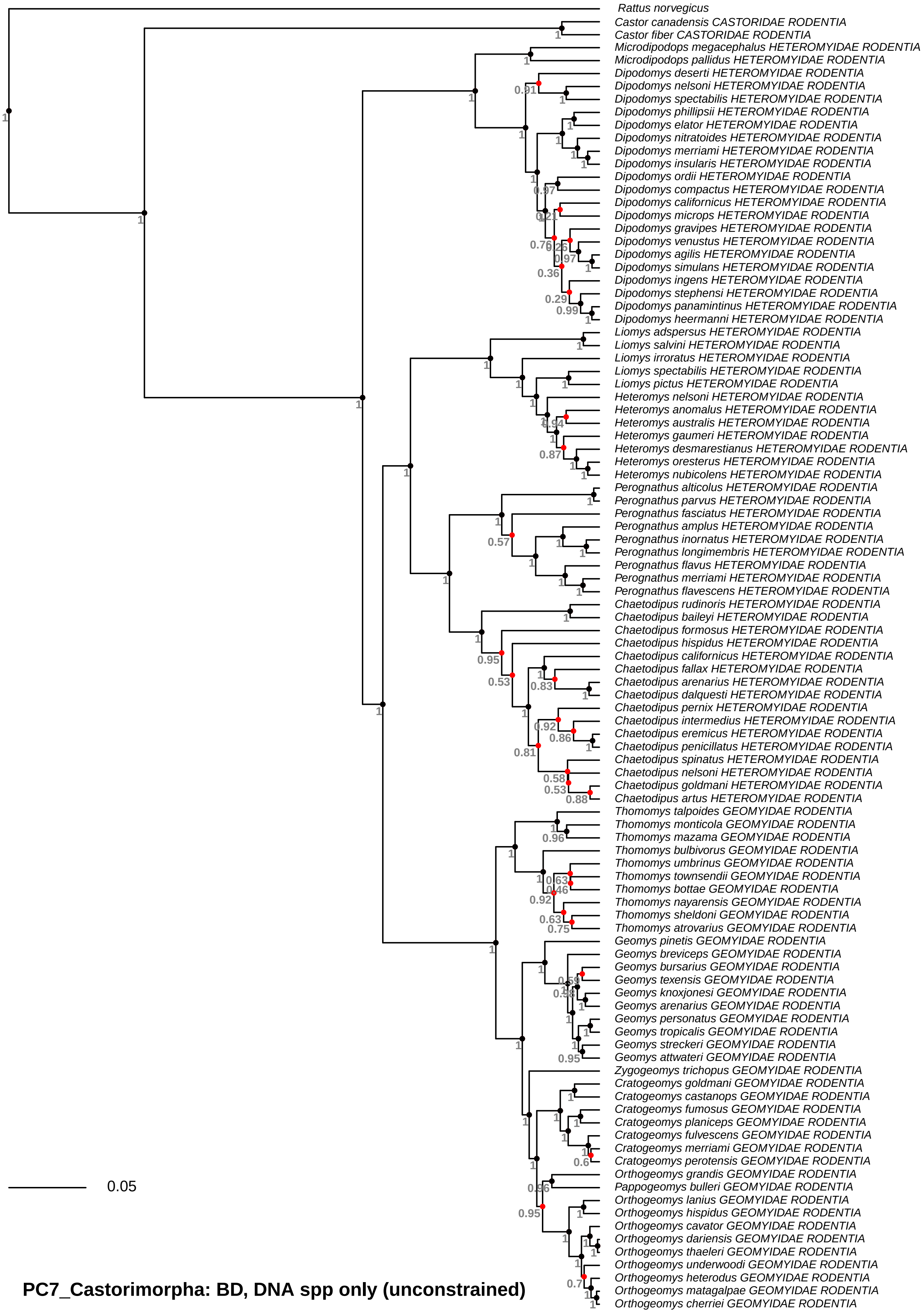

PC7_Castorimorpha: BD, DNA spp only (unconstrained)

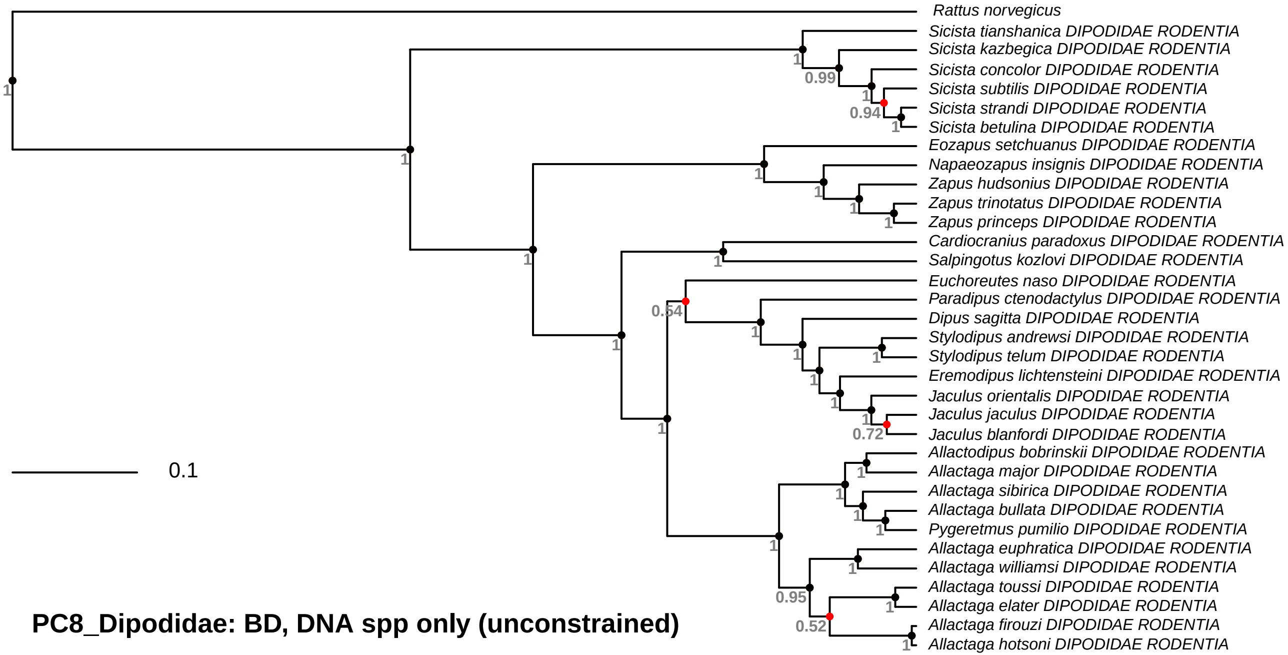

PC8_Dipodidae: BD, DNA spp only (unconstrained)

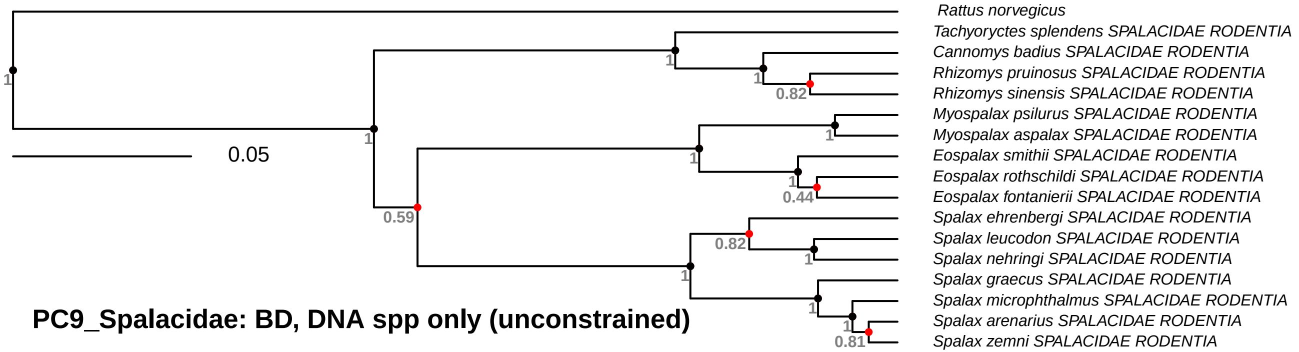

PC9_Spalacidae: BD, DNA spp only (unconstrained)

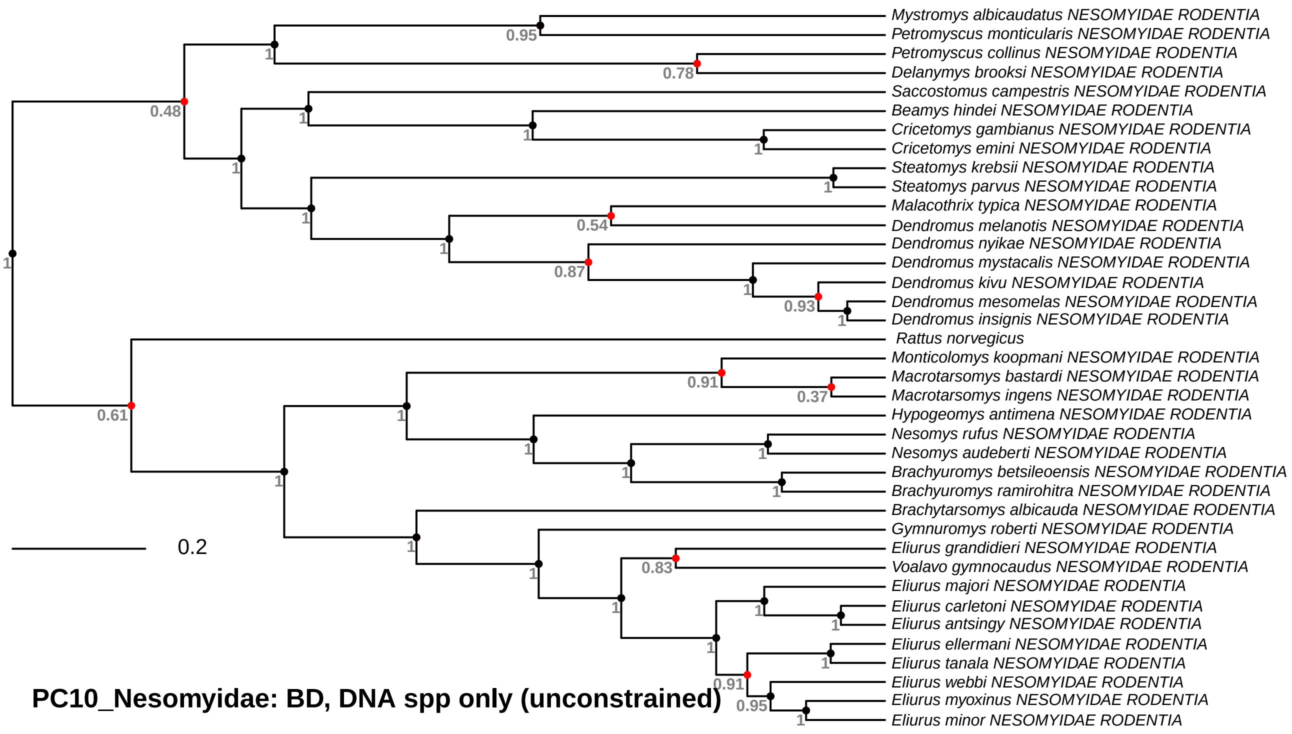

PC10_Nesomyidae: BD, DNA spp only (unconstrained)

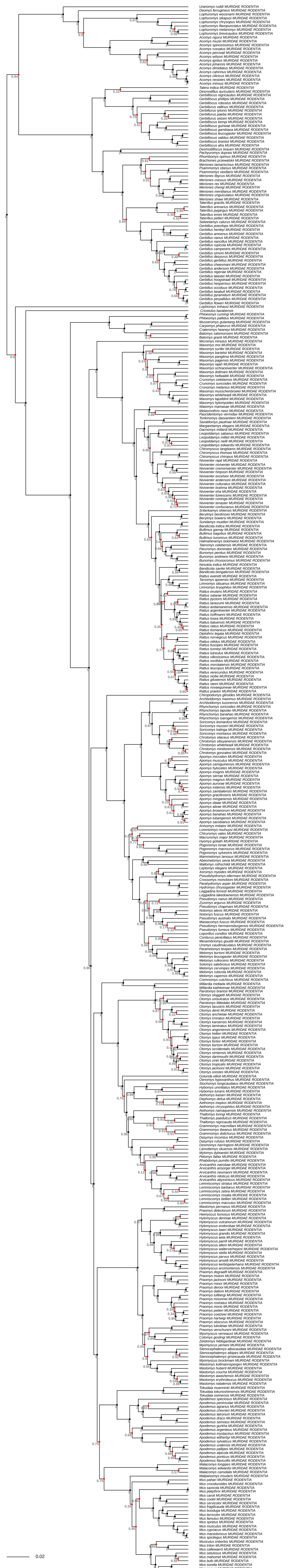

PC11_Muridae: BD, DNA spp only (unconstrained)

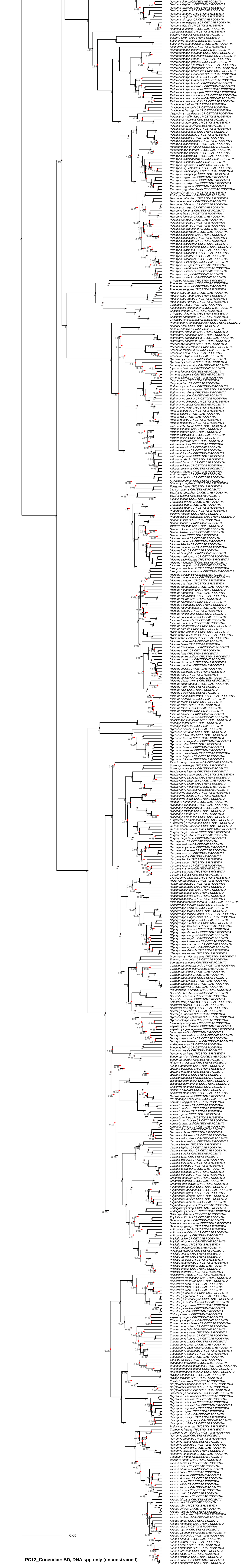

PC12_Cricetidae: BD, DNA spp only (unconstrained)

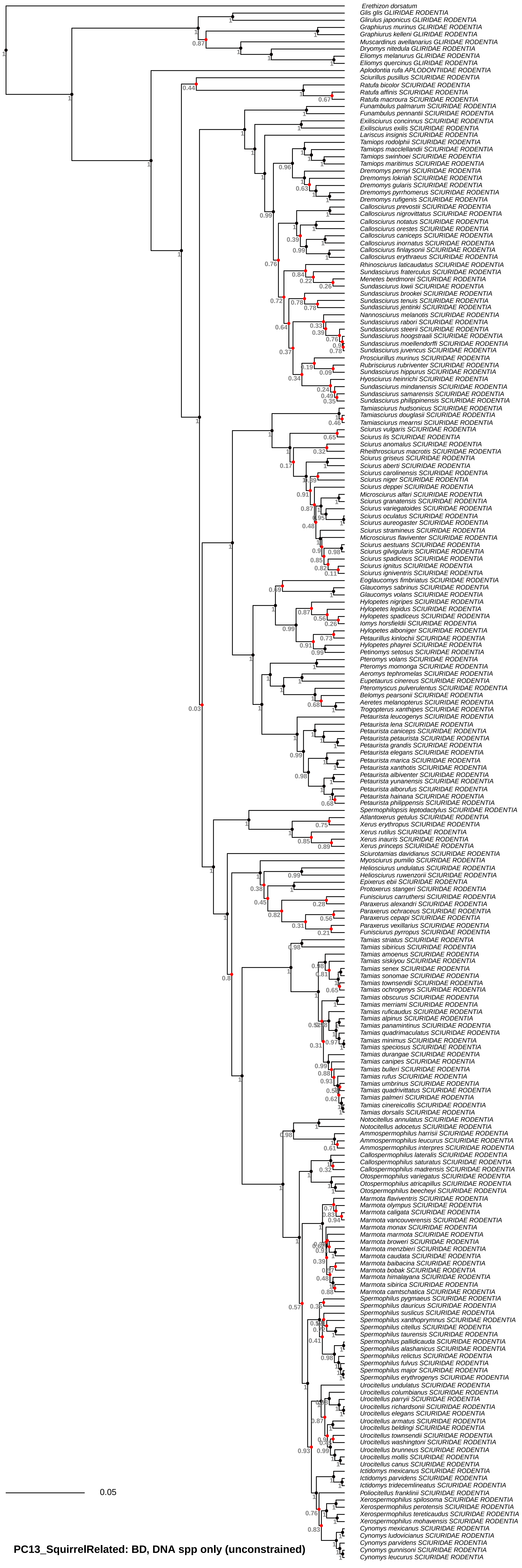

PC13_SquirrelRelated: BD, DNA spp only (unconstrained)

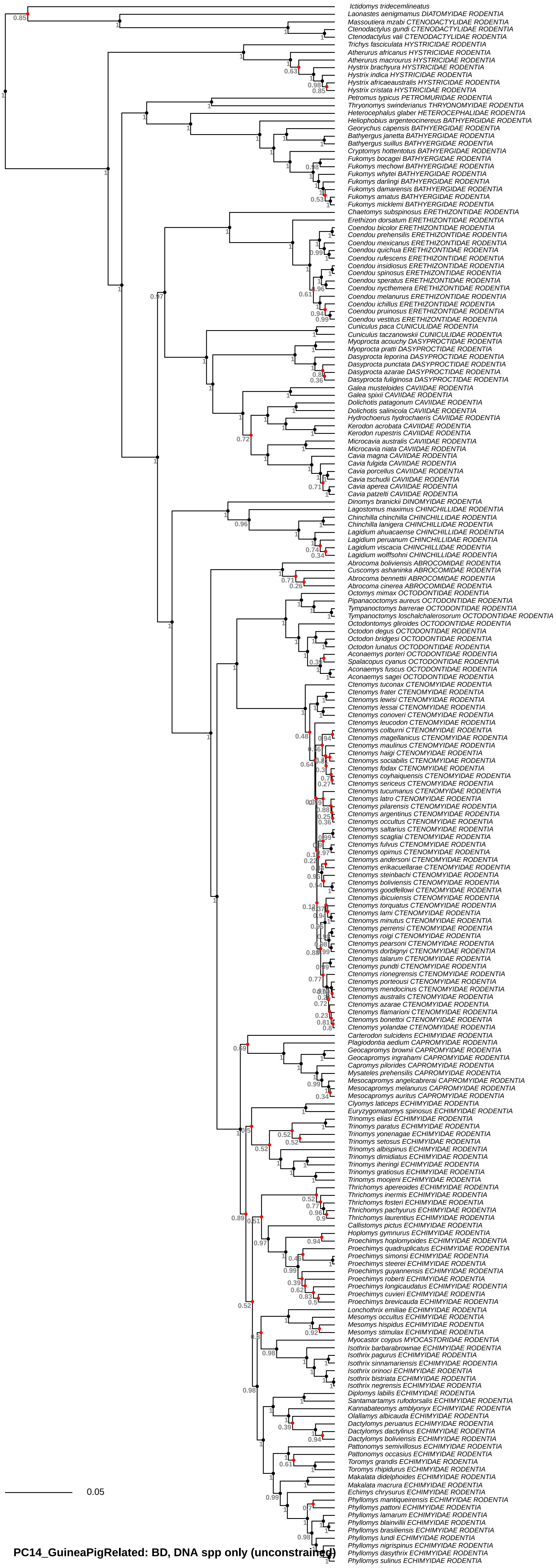

PC14_GuineaPigRelated: BD, DNA spp only (unconstrained)

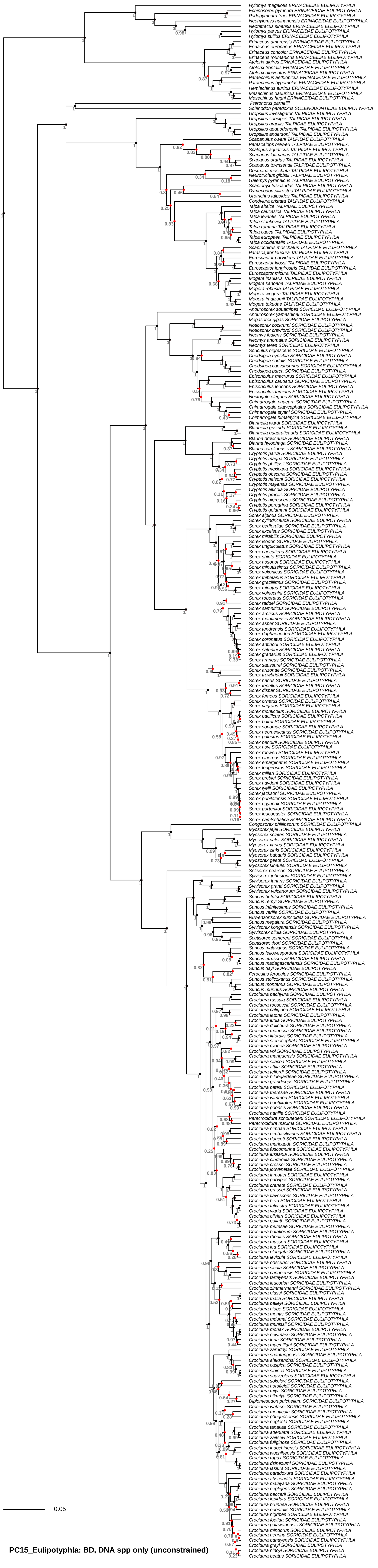

PC15_Eulipotyphla: BD, DNA spp only (unconstrained)

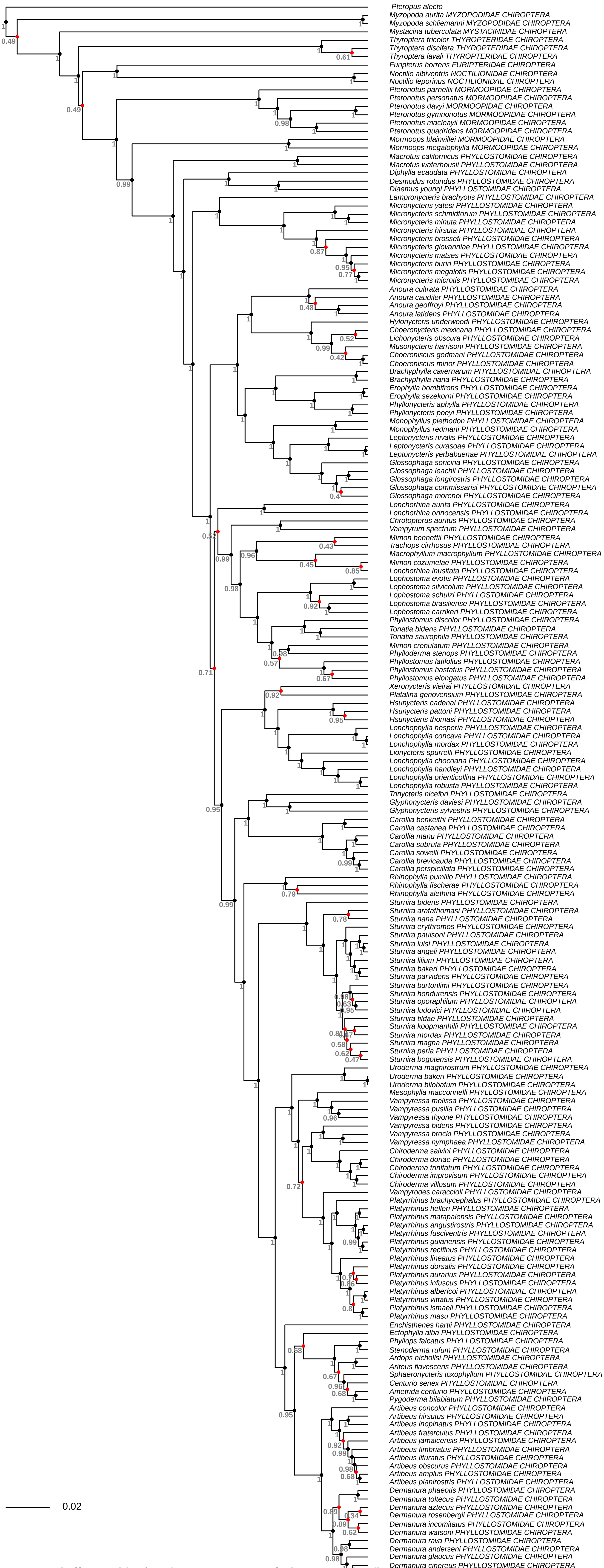

PC16_PhyllostomidRelated: BD, DNA spp only (unconstrained)

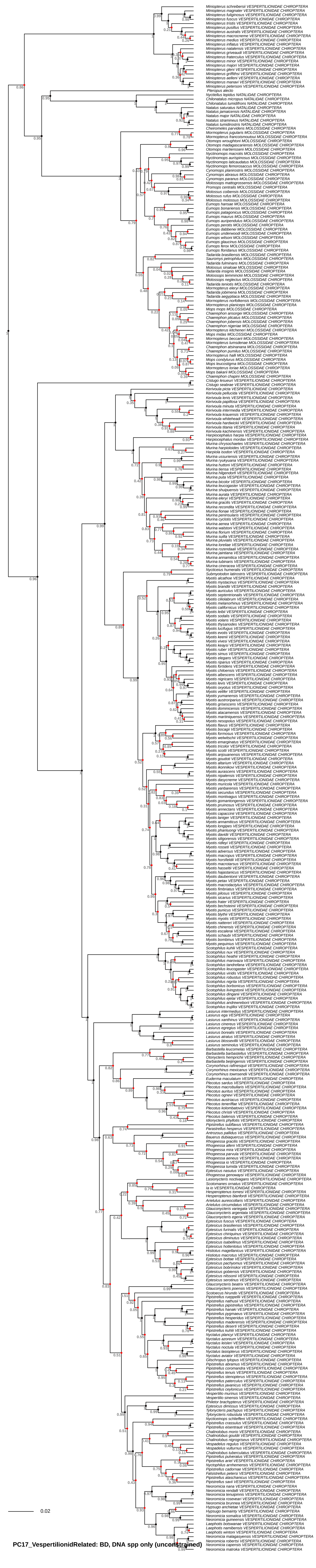

PC17_VespertilionidRelated: BD, DNA spp only (unconstrained)

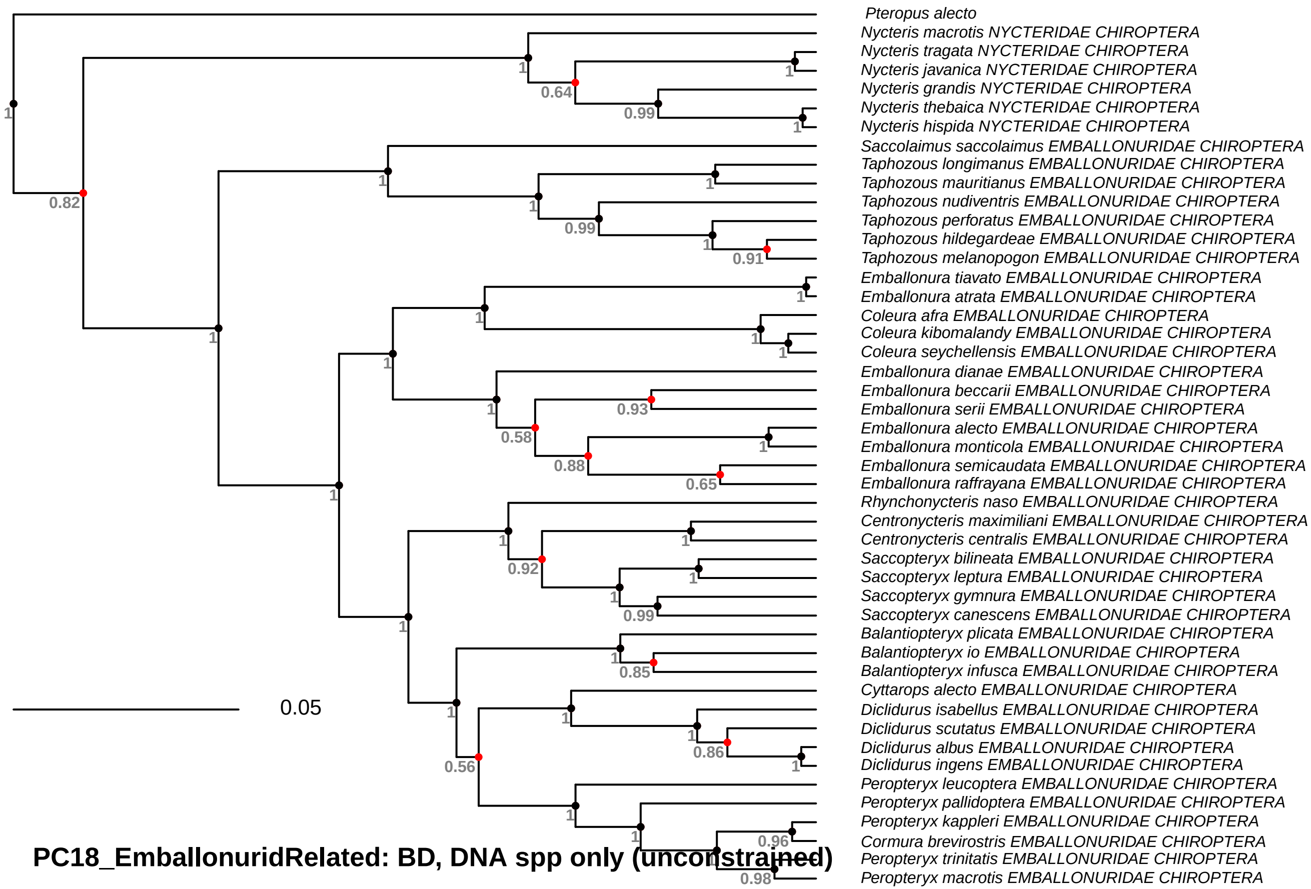

PC18_EmballonuridRelated: BD, DNA spp only (unconstrained)

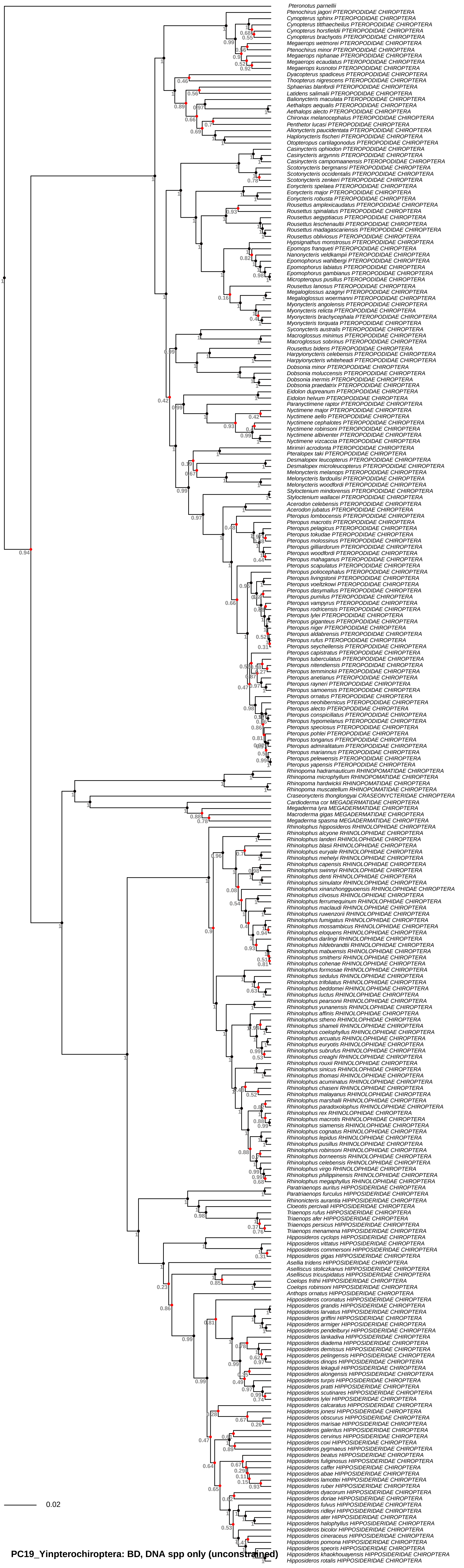

PC19_Yinpterochiroptera: BD, DNA spp only (unconstrained)

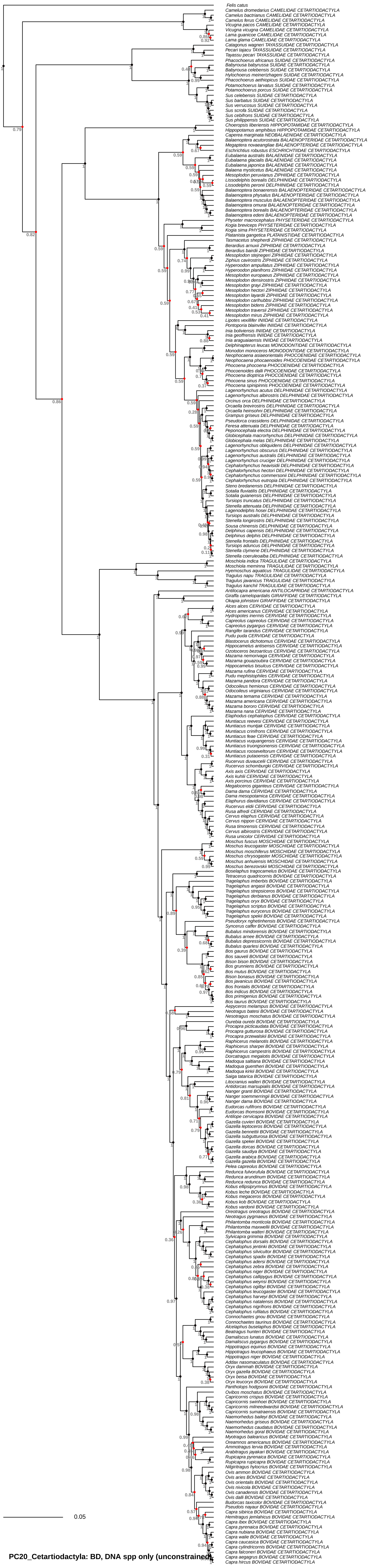

PC20_Cetartiodactyla: BD, DNA spp only (unconstrained)

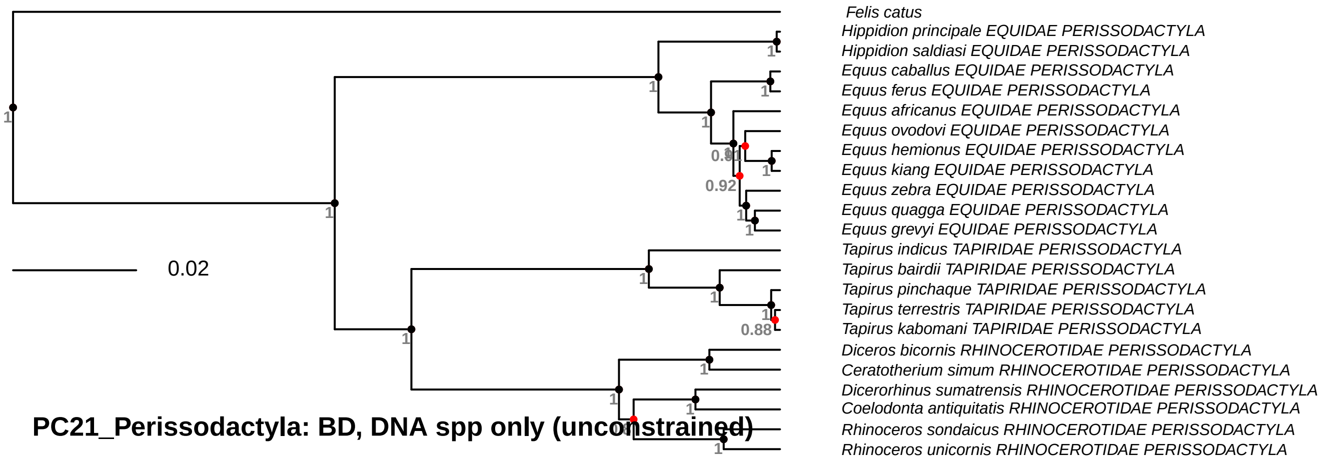

PC21_Perissodactyla: BD, DNA spp only (unconstrained)

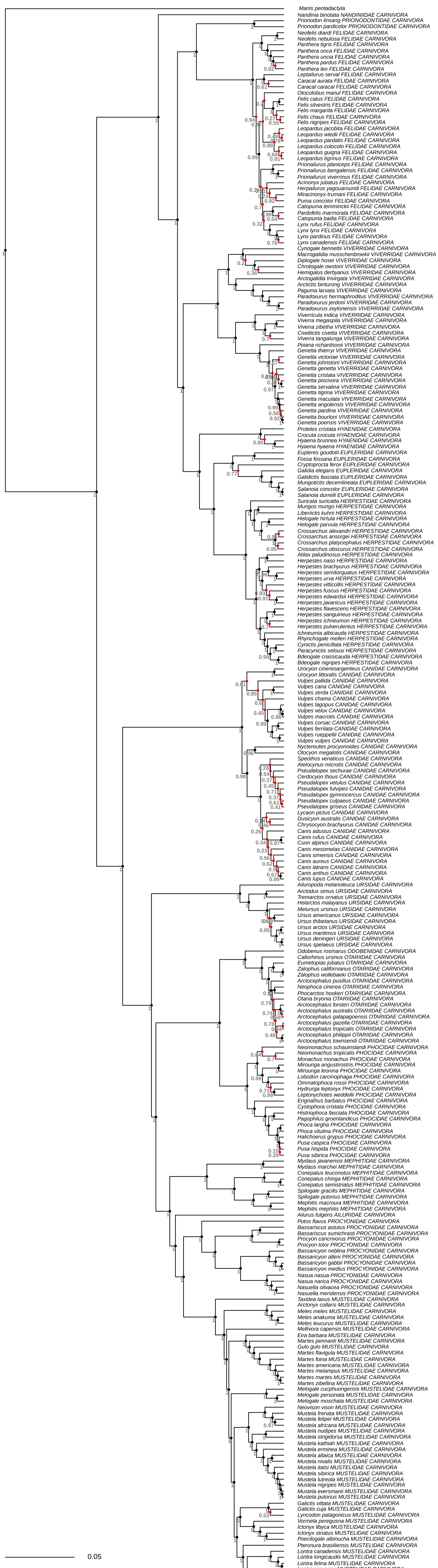

0.05

PC22_Carnivora: BD, DNA spp only (unconstrained)

PC23_Monotremata: BD, DNA spp only (unconstrained)

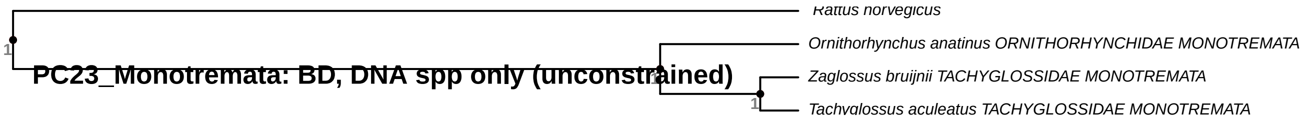

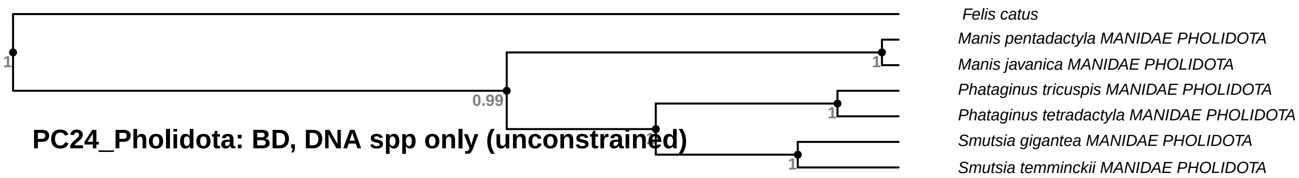
PC24_Pholidota: BD, DNA spp only (unconstrained)
Felis catus
Manis pentadactyla MANIDAE PHOLIDOTA
Manis javanica MANIDAE PHOLIDOTA
Phataginus tricuspis MANIDAE PHOLIDOTA
Phataginus tetradactyla MANIDAE PHOLIDOTA
Smutsia gigantea MANIDAE PHOLIDOTA
Smutsia temminckii MANIDAE PHOLIDOTA
1
0.99
1
1
1

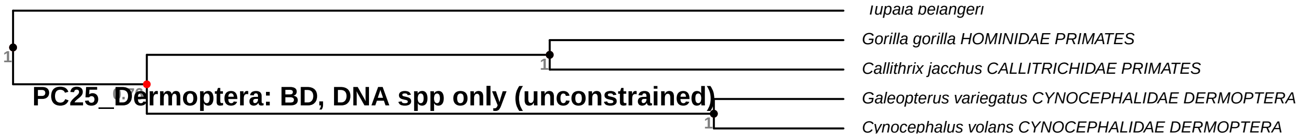
PC25_Dermoptera: BD, DNA spp only (unconstrained)
Tupaia belangeri
Gorilla gorilla HOMINIDAE PRIMATES
Callithrix jacchus CALLITRICHIDAE PRIMATES
Galeopterus variegatus CYNOCEPHALIDAE DERMOPTERA
Cynocephalus volans CYNOCEPHALIDAE DERMOPTERA
1
1
1

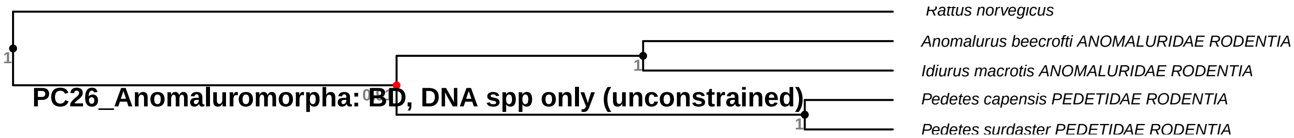

PC26_Anomaluromorpha: BD, DNA spp only (unconstrained)

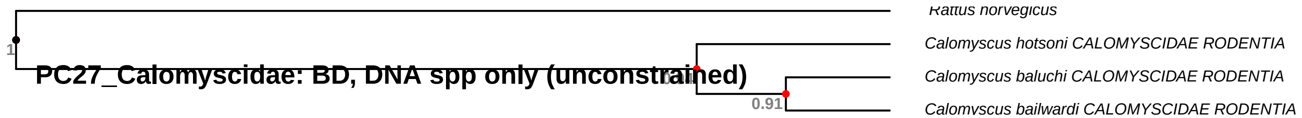
PC27_Calomyscidae: BD, DNA spp only (unconstrained)
Rattus norvegicus
Calomyscus hotsoni CALOMYSCIDAE RODENTIA
Calomyscus baluchi CALOMYSCIDAE RODENTIA
Calomyscus bailwardi CALOMYSCIDAE RODENTIA
1
0.91

PC28_Platacanthomyidae: BD, DNA spp only (unconstrained)

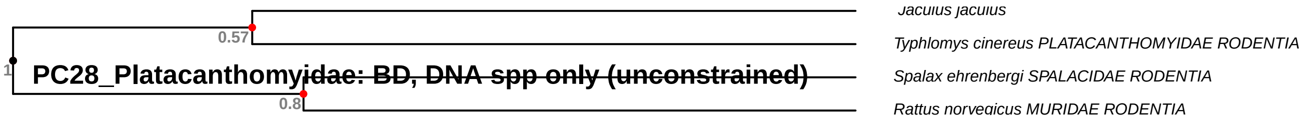

Supplement: S4 Data — Relative time results (i.e., not yet time scaled) presented as MCC trees for each of the patch clade runs, both as nexus and PDF plots, and details of the species and gene sampling in each patch clade. Dryad data: https://doi.org/10.5061/dryad.tb03d03. MCC, maximum clade credibility. (ZIP) [file pbio.3000494.s024.zip › S4_Data.zip!/Data_S5_patchClade_results_and_MCC/PatchClades_MCC_DNAonly-TopoFree_mean/ALL28plots_postburn10k_BDvr_FREE_MCCs.pdf]
