# Supplementary material for: Inferring the mammal tree: Species-level sets of phylogenies for questions in ecology, evolution, and conservation
Source: PLoS Biol. 2019 Dec 4;17(12):e3000494. doi: 10.1371/journal.pbio.3000494 (PMC6892540; doi:10.1371/journal.pbio.3000494)
Supplement: S4 Data — Relative time results (i.e., not yet time scaled) presented as MCC trees for each of the patch clade runs, both as nexus and PDF plots, and details of the species and gene sampling in each patch clade. Dryad data: https://doi.org/10.5061/dryad.tb03d03. MCC, maximum clade credibility. (ZIP) [file pbio.3000494.s024.zip › S4_Data.zip!/Data_S5_patchClade_results_and_MCC/PatchClades_MCC_DNAonly-TopoFree_mean/postburn10k_BDvr_FREE_MCC_PC28_Platacanthomyidae_plot.pdf]

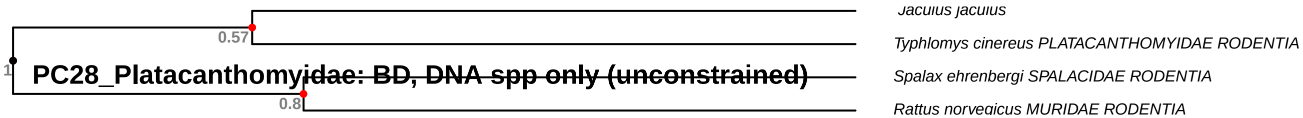
PC28_Platacanthomyidae: BD, DNA spp only (unconstrained)
Jaculus jaculus
Typhlomys cinereus PLATACANTHOMYIDAE RODENTIA
Spalax ehrenbergi SPALACIDAE RODENTIA
Rattus norvegicus MURIDAE RODENTIA
0.57
1
0.8
