# Supplementary material for: Inferring the mammal tree: Species-level sets of phylogenies for questions in ecology, evolution, and conservation
Source: PLoS Biol. 2019 Dec 4;17(12):e3000494. doi: 10.1371/journal.pbio.3000494 (PMC6892540; doi:10.1371/journal.pbio.3000494)
Supplement: S4 Data — Relative time results (i.e., not yet time scaled) presented as MCC trees for each of the patch clade runs, both as nexus and PDF plots, and details of the species and gene sampling in each patch clade. Dryad data: https://doi.org/10.5061/dryad.tb03d03. MCC, maximum clade credibility. (ZIP) [file pbio.3000494.s024.zip › S4_Data.zip!/Data_S5_patchClade_results_and_MCC/PatchClades_MCC_DNAonly-TopoFree_mean/postburn10k_BDvr_FREE_MCC_PC1_Marsupials_plot.pdf]

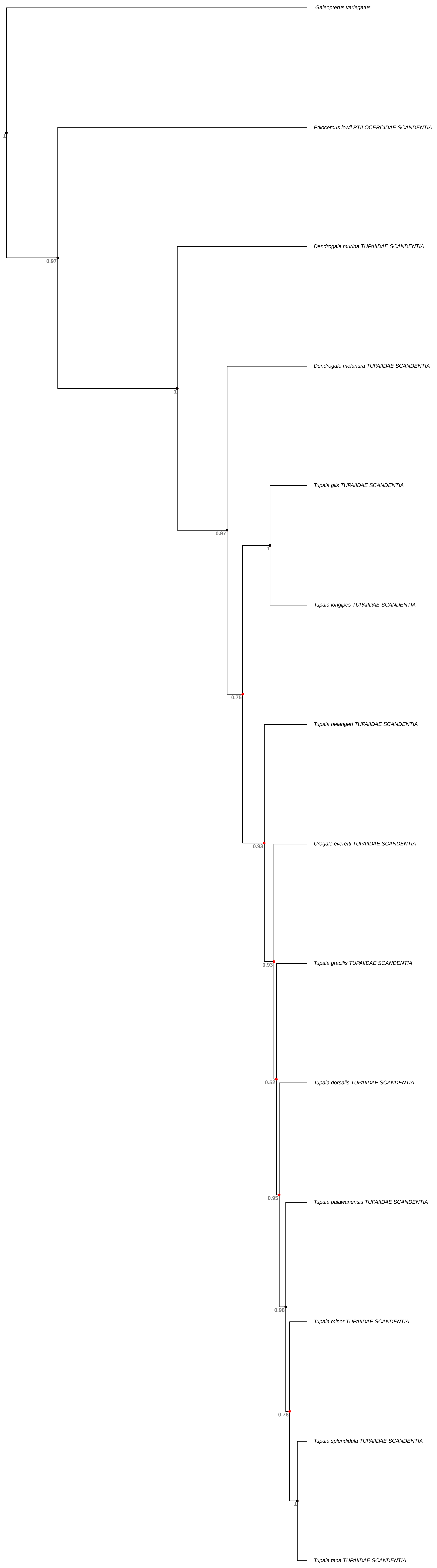

Galeopterus variegatus
Ptilocercus lowii PTILOCERCIDAE SCANDENTIA
Dendrogale murina TUPAIIDAE SCANDENTIA
Dendrogale melanura TUPAIIDAE SCANDENTIA
Tupaia glis TUPAIIDAE SCANDENTIA
Tupaia longipes TUPAIIDAE SCANDENTIA
Tupaia belangeri TUPAIIDAE SCANDENTIA
Urogale everetti TUPAIIDAE SCANDENTIA
Tupaia gracilis TUPAIIDAE SCANDENTIA
Tupaia dorsalis TUPAIIDAE SCANDENTIA
Tupaia palawanensis TUPAIIDAE SCANDENTIA
Tupaia minor TUPAIIDAE SCANDENTIA
Tupaia splendidula TUPAIIDAE SCANDENTIA
Tupaia tana TUPAIIDAE SCANDENTIA
1
0.97
1
0.97
1
0.75
0.93
0.93
0.52
0.95
0.98
0.76
1
